# Loss of aquaporin-4 expression and putative function in non-small cell lung cancer

**DOI:** 10.1186/1471-2407-11-161

**Published:** 2011-05-06

**Authors:** Arne Warth, Thomas Muley, Michael Meister, Esther Herpel, Anita Pathil, Hans Hoffmann, Philipp A Schnabel, Christian Bender, Andreas Buness, Peter Schirmacher, Ruprecht Kuner

**Affiliations:** 1 Institute of Pathology, University Hospital Heidelberg, Germany; 2 Translational Research Unit, Thoraxklinik Heidelberg, Germany; 3 Department of Internal Medicine IV, University Hospital Heidelberg, Germany; 4 Department of Thoracic Surgery, Thoraxklinik Heidelberg, Germany; 5 Division of Molecular Genome Analysis, German Cancer Research Center, Heidelberg, Germany

## Abstract

**Background:**

Aquaporins (AQPs) have been recognized to promote tumor progression, invasion, and metastasis and are therefore recognized as promising targets for novel anti-cancer therapies. Potentially relevant AQPs in distinct cancer entities can be determined by a comprehensive expression analysis of the 13 human AQPs.

**Methods:**

We analyzed the presence of all AQP transcripts in 576 different normal lung and non-small cell lung cancer (NSCLC) samples using microarray data and validated our findings by qRT-PCR and immunohistochemistry.

**Results:**

Variable expression of several AQPs (AQP1, -3, -4, and -5) was found in NSCLC and normal lung tissues. Furthermore, we identified remarkable differences between NSCLC subtypes in regard to AQP1, -3 and -4 expression. Higher transcript and protein levels of AQP4 in well-differentiated lung adenocarcinomas suggested an association with a more favourable prognosis. Beyond water transport, data mining of co-expressed genes indicated an involvement of AQP4 in cell-cell signalling, cellular movement and lipid metabolism, and underlined the association of AQP4 to important physiological functions in benign lung tissue.

**Conclusions:**

Our findings accentuate the need to identify functional differences and redundancies of active AQPs in normal and tumor cells in order to assess their value as promising drug targets.

## Background

The human body consists of about 70% water and the regulation of water and thus ion homeostasis is a basic cell function. The assumption of water freely crossing cell membranes by pure diffusion was changed by detection of water-selective channels which are now known as aquaporins (AQPs). Selective water transport is essential to maintain the cellular electrochemical potential. In mammals, 13 members of the aquaporin gene family (AQP0 through AQP12) have been identified [1]. AQPs have been found to play a pro-tumorigenic role in different tumor types. Besides the transport of water across biological membranes as their main role in normal cells, AQP-expressing cancer cells show enhanced migration *in vitro*, and increased invasiveness, extravasation, and potential to metastasize *in vivo *[2]. In contrast to the pro-tumorigenic function caused by enhanced AQP expression in some tumors, reduced expression of AQPs in hepatocellular carcinoma is associated with increased resistance to apoptosis [3]. However, the pro-tumorigenic and/or anti-apoptotic functions of AQPs are poorly understood. Recently, first data demonstrated the potential usage of AQP inhibition in anti-cancer therapies also in humans [4]. Current theories associate AQP activity with osmotic pressure increase to form cell protrusions essential for migration while aquaglyceroporins may play a key role in tumor energy metabolism [2,5-8]. A further, yet not sufficiently considered possibility for pro-tumorigenic functions of AQPs is the direct or indirect regulation of other genes, for example stabilization of hypoxia-inducible factor, which is facilitated by AQP1 expression [9] or the interaction of AQP5 with the Ras/extracellular signal-regulated kinase/retinoblastoma protein signaling pathway [10]. Since AQPs have been recognized as promising targets for novel anti-cancer therapies [11,12], it is important to elucidate their tumor-specific expression patterns and to reveal their potential functions besides sole water transport in the different organs and tumor entities.

In the present study, we focused on non-small cell lung cancer (NSCLC), the most common malignant lung tumor, in particular its two major histological subtypes, adenocarcinoma (AC) and squamous cell carcinoma (SCC). In normal human lungs expression of AQP1, -3, -4, and -5 has been described [13]. Only little is known about the expression of AQPs in NSCLCs: AQP1 is expressed in AC [14,15] and it was shown that AQP1 expression facilitated tumor cell migration and spread whereas AQP1 inhibition reduced the metastatic potential of tumor cells [16]. AQP3 is also expressed in NSCLCs but more prominent in AC than in SCC [17]. The expression of AQP4 and the potential roles of both AQP3 and AQP4 in lung carcinogenesis are unknown. Overexpression of AQP5 has most recently been reported to promote tumor invasion of human NSCLCs [18,19]. In order to get more insight into the expression patterns of AQPs in human NSCLCs, its relations to patient survival and to other cellular processes besides water transport and to provide a basis for further functional characterization we analyzed independent microarray studies and validated the expression of the most prominent AQPs in matched NSCLC and normal specimens by quantitative real-time PCR analyses. Additionally, AQP expression was investigated in nine NSCLC cell lines to identify potential AQP-related intervention models. The expression of AQP4, yet unknown in NSCLCs, was further analyzed by immunohistochemistry and western blotting.

## Methods

### Patient population and tissue specimens

All tumors were removed in the Department of Thoracic Surgery, University Hospital Heidelberg, and diagnoses were confirmed by at least two experienced pathologist according to the current WHO classification for lung cancer [20]. All patients provided appropriate informed consent. For qRT-PCR analysis, we used a previously described sample collective of 105 NSCLC and normal lung tissues including matched tumors and normal tissues from 45 patients [21]. The usage of all tissues for this study was approved by the local ethics committee (No. 206/2005).

### AQP gene expression analysis by data mining of independent NSCLC microarray studies

Expression of AQP genes was analyzed using five independent microarray studies [22,21,25]. The microarray data was downloaded from NCBI GEO database (GSE10245, GSE8894, GSE3398) or kindly provided upon request [23,22]. We used only data from AC, SCC, and normal lung samples. The datasets comprised 576 different microarray profiles (Additional file 1, Table S1). First, pre-processing and the selection of representative sequences of the AQPs from the four different microarray platforms were performed using Bioconductor and R as previously described [26]. The datasets were separately investigated for AQP isoforms gene expression variation in AC (n = 417), SCC (n = 127), and normal lung tissues (n = 32). Student's t-Test was used to analyze differential expression of distinct AQPs between tumor and normal samples or between the two subtypes AC and SCC (Additional file 2, Table S2). A fold change was calculated by dividing the medians of linear expression values in each comparison. In the case of different features per gene, we used the median of the present expression values.

For AQP4, we determined the overlap of the 100 highest correlated and 100 highest anti-correlated genes across all microarray datasets ranked by Pearson correlation (Additional file 3, Table S3). The gene signatures were further examined using Ingenuity Pathways Knowledge Base (Ingenuity, Mountain View, USA), one of the largest manual curated database, to identify associations with cellular functions and diseases. Hierarchical clustering of genes and samples was based on Manhattan distance measures.

### Quantitative real-time PCR

Total RNA was extracted using the RNeasy Kit (Qiagen, Hilden, Germany) according to the manufacturer's instructions. Reverse transcription was performed with 2 μg total RNA per reaction using RevertAid™ First Strand cDNA Synthesis Kit (Fermentas, Burlington, ON, Canada). The amount of cDNA equivalent to 5 ng total RNA was included in each PCR reaction. Expression analysis of five AQPs (AQP1, -3, -4, -5, -9) and the housekeeping gene esterase D (*ESD*) was performed in 105 different tissue specimens and nine cell lines by quantitative real-time PCR (ABI Prism 7900HT Sequence Detection System; Applied Biosystems, Weiterstadt, Germany). The procedure was previously described in detail [21]. Briefly, we used gene specific primer and probe Taqman assays (ABI) and performed relative quantification by delta-delta Ct method using the housekeeping gene esterase. Raw and processed data are given in Additional file 4, Table S4. Gene expression differences between sample groups were analyzed using t-test or paired t-test. BoxPlot presentation was generated by GraphPad prism, version 2.01 (GraphPad Software, San Diego, CA).

### Tissue microarray construction

A tissue microarray (TMA) containing 125 early stage NSCLC specimens (clinical stage I and II) and the corresponding non-neoplastic lung tissue was constructed as described previously [27]. Briefly, the TMA contained tissue samples of 52 AC, 49 SCC, 17 pleomorphic carcinomas, 3 basaloid carcinomas, 2 adeno-squamous carcinomas, and 2 large cell carcinomas. Prior to TMA construction a HE-stained slide of each block was analyzed in order to select the inappropriate regions (e.g. excluding necrosis, haemorrhage) for the TMA slides. A TMA machine (AlphaMetrix Biotech, Rödermark, Germany) was used to extract a 1.6 mm cylindrical core sample from the tissue donor block.

### Immunohistochemistry and statistical analyses

Immunohistochemical staining was performed according to a standardized protocol using the following primary antibody: rabbit polyclonal anti-AQP4 against the epitope corresponding to amino acids 244-323 of human origin (dilution: 1:100; Santa Cruz Biotechnology, Santa Cruz, CA, USA). The TMA slides were deparaffinized and pre-treated with an antigen retrieval buffer (pH 6.0; DAKO, Hamburg, Germany). Subsequent steps were carried out in an immunostaining device (DAKO Techmate 500plus). The immunostaining protocol was based on the avidin-biotin peroxidase principle using AEC as the chromogen, as well as haematoxilin for counterstaining. As positive controls we used human kidney sections as a common source of AQP4. For negative controls, the primary antibody was omitted. For optimized evaluation and scoring the stained TMAs were scanned with a resolution of 0.25 μm/pixel using a ScanScope CS System and analyzed using ImageScope (both Aperio Technologies, Vista, CA, USA).

A semi-quantitative evaluation of the AQP4 immunoreactivity was done scoring both the staining intensity (no staining = 0, weak = 1, moderate = 2, strong = 3) and the amount of positively stained cells (0 = 0%, 1 = 1-20%, 2 = 21-50%, 3 = 51-80%, 4 = >80%). The multiplied scoring resulted in an immunoreactivity score (IRS) between 0 and 12. All available survival data from the AC patients (n = 46) were used for Kaplan-Meier analyses. Mean post-operative follow-up was 1172 days. At the time point of this study 31 patients were alive (Additional file 5, Table S5).

### Western blotting

For western blotting we selected tumor samples (six adenocarcinomas and one normal lung tissue) with available fresh frozen tissue. Total protein extracts were prepared from 5 μm slices of fresh frozen tissue samples. The slices were crushed for 30 seconds in cell lysis buffer (Cell Signaling Technology, Boston, USA), centrifuged at 14,000 *rpm *at 4°C for 10 minutes, and quantified using the Bradford assay (Bio-Rad Laboratories, München, Germany). 25 μg of cleared supernatant was diluted in 3× sample buffer, heated 5 min. at 95°C, loaded onto a 15% sodium dodecyl sulphate polyacrylamide gel electrophoresis (25 μg/lane), and electro-transferred to a polyvinylidene fluoride membrane. The membrane was blocked in Tris-buffered saline/Tween with 5% milk powder (TBST) for 1 h. We used the same primary anti-AQP4 antibody as for immunohistochemistry (1:200; Santa Cruz Biotechnology, CA, USA), and anti-actin antibody (1:10000; Santa Cruz Biotechnology, CA, USA) diluted in TBST and incubated at 4°C overnight. The appropriate secondary antibody was applied (1:2000; horseradish peroxidase anti-mouse and horseradish peroxidase anti-rabbit) at room temperature for 1 hour. Visualization was performed by enhanced chemiluminescence (PerkinElmer Life and Analytical Sciences, Shelton, USA). Blots were subjected to quantitative analysis using ImageJ software. The AQP4/actin-ratios are given in densitometry units under the blots.

## Results

### Comprehensive AQP expression analyses in NSCLC reveal a predominant expression of AQP1, -3, and -4 in adenocarcinomas

We investigated the AQP expression in normal lung and NSCLC tumor samples and in AC and SCC tumor subtypes. Gene expression values of AQPs 1-12 were available in at least two independent datasets (Additional file 2, Table S2). Considering the relative expression level ranks of AQPs across all present genes in each dataset, we observed a wide intensity range from low (AQP8, -10, -11 and -12) to moderate (AQP4, -5, -6 and -7) and high abundancy (AQP1, -2, -3 and -9). AQP1, -3, -4, -5 and -9 were differentially expressed between tumor and normal or AC and SCC subtype in at least one microarray dataset (p-value < 0.05; Fold change ≥ 2 or ≤ 0.5), and therefore selected for further qRT-PCR analysis (Table 1). The expression of the five AQPs could be measured in nearly all NSCLC and normal lung samples (Additional file 4, Table S4). Widely consistent with the microarray data AQP1, -3 and -4 were expressed in a similar range (median Ct values: 27.8, 26.9, 28.7, respectively), whereas AQP5 and -9 transcripts were less abundant (median Ct values: 31.8, 31.0). Furthermore, AQP1 and -4 expression was highly correlated across the 105 lung cancer and normal samples (Pearson correlation: 0.82) as previously observed in the microarray data.

**Table 1:** Five AQPs were identified to be deregulated (* p-value < 0.05; Fold change ≥ 2 or ≤ 0.5) in tumors versus normal tissues, or in AC versus SCC subtype across different microarray studies

AQP isoform	Microarray Data		qRT-PCR Data		Median Ct value in tissues	Pearson correlation of AQPs			
	**Tumor vs Normal Fold change**	**AC vs SCC Fold change**	**Tumor vs Normal Fold change**	**AC vs SCC Fold change**		**AQP1**	**AQP3**	**AQP4**	**AQP5**

AQP1	0.18*	7.5*	0.22*	2.36*	27.8	X	x	x	x

AQP3	1.00	4.8*	0.93	5.81*	26.9	0.46	x	x	x

AQP4	0.16*	2.8*	0.11*	5.96*	28.7	**0.82**	0.42	x	x

AQP5	0.94	6.9*	0.50*	1.88	31.8	0.31	0.40	0.24	x

AQP9	0.26*	0.91	1.05	0.64	31.0	0.39	0.34	0.30	0.10

The results were mainly confirmed by qRT-PCR including information about expression range in all tissues and Pearson correlation between different AQPs.

AQP: aquaporin; AC: adenocarcinoma; SCC: squamous cell carcinoma; fold change was calculated across microarray datasets (median); fold change in bold and marked (*) indicates statistical significance (p-value < 0.05).

**Figure 1:**
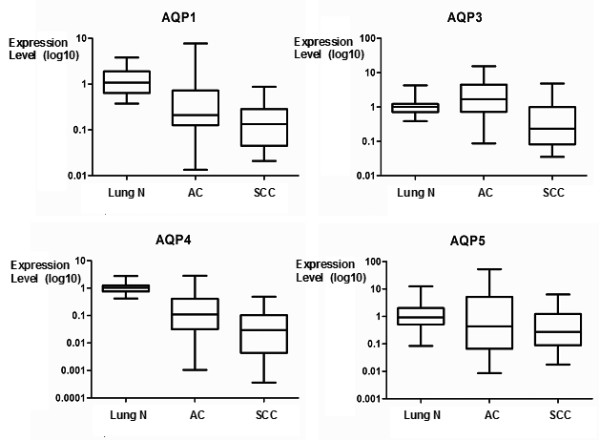
**Differential gene expression of distinct AQPs in NSCLC**. Gene expression of AQP1, -3, -4 and -5 in AC (n = 40), SCC (n = 16) and normal normal lung tissues (n = 49) including matched cases (n = 45) using qRT-PCR data. T-test results and fold changes for all comparisons are given in Table 2.

Although a direct comparison is restricted due to differences in cellular composition between normal lung and NSCLC samples, AQP expression levels showed an overall lower expression of AQP1, -4 and -5 in NSCLC compared to the respective normal lung tissues of 45 patients (Table 2, Figure 1). Only AQP3 was higher expressed in AC compared to the normal lung. Furthermore, the microarray data indicated a higher expression of AQP1, -3, -4 and -5 in AC compared to SCC subtype. The qRT-PCR results were in agreement with the microarray data (except for AQP5) that revealed higher expression of AQP1, -3 and -4 in AC compared to SCC. All other AQPs showed very little or no expression differences in these comparisons.

**Table 2:** AQP1, -3, -4, -5 and -9 gene expression in 105 NSCLC and normal samples by qRT-PCR data

AQP isoform	Tumor vs Normal		AC vs SCC		AC MT vs MN		SCC MT vs MN		Survival in AC: Good vs Bad	
	**p-value**	**Fold change**	**p-value**	**Fold change**	**p-value**	**Fold change**	**p-value**	**Fold change**	**p-value**	**Fold change**

AQP1	**0.0000**	**0.22**	**0.0228**	**2.36**	**0.0000**	**0.26**	**0.0000**	**0.10**	**0.0340**	**2.86**

AQP3	0.7089	0.93	**0.0005**	**5.81**	0.0205	1.76	0.0676	0.38	0.7165	0.81

AQP4	**0.0000**	**0.11**	**0.0144**	**5.96**	**0.0000**	**0.12**	**0.0002**	**0.02**	**0.0344**	**4.42**

AQP5	**0.0253**	**0.50**	0.3255	1.88	0.1640	0.55	0.1956	0.39	0.1937	0.25

AQP9	0.7384	1.05	0.1310	0.64	0.6103	0.91	0.7887	1.12	0.7480	1.15

Student's T-Test comparison between matched tumor (MT) and normal lung tissue (MN) collectives (n = 45), different histology (AC vs SCC) and survival (good >3 y, bad <3 y).

AQP: aquaporin; AC: adenocarcinoma; SCC: squamous cell carcinoma; p-value and fold change in bold indicate statistical significance.

Comparing expression data of the ACs with follow-up data indicated a better prognosis when AQP1 (p = 0.0340) and -4 (p = 0.0344) were higher expressed (Table 2). None of the other AQPs indicated an association to prognostic parameters in AC, SCC or both. Of note, AQP4 showed the strongest transcriptional deregulation between tumor and normal tissues (fold change: 0.11), the subtypes AC and SCC (6.0) and AC patient collectives with good and bad survival (4.4), respectively.

### AQP4-protein is more abundant in well-differentiated adenocarcinomas

**Figure 2:**
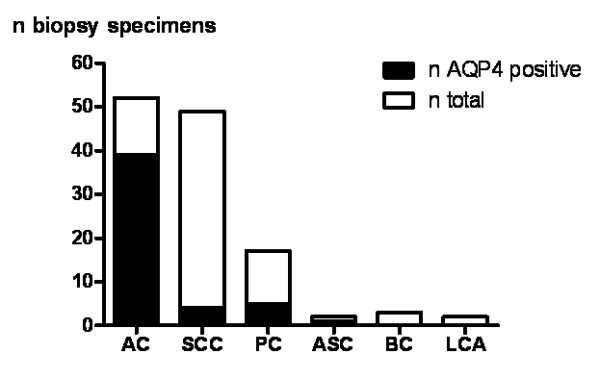
**Immunohistochemical analysis of AQP4 expression in 125 NSCLC specimens**. Adenocarcinoma (AC), Squamous Cell Carcinoma (SCC), Pleomorphic Carcinoma (PC), Adeno-Squamous Carcinoma (ASC), Basaloid Carcinoma (BC), Large Cell Carcinoma (LCA).

**Figure 3:**
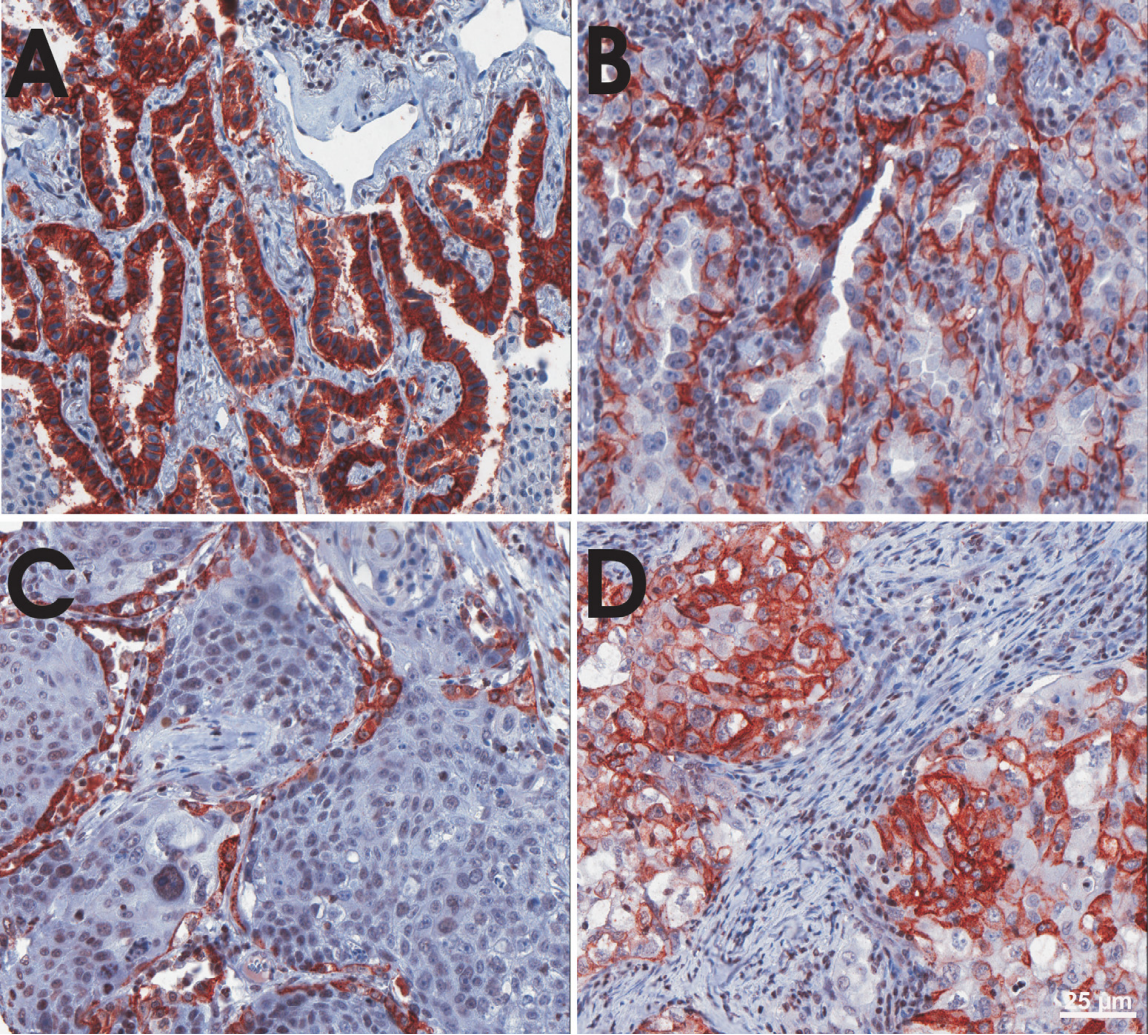
**AQP4-protein expression pattern in NSCLCs**. AQP4 is mainly expressed in well differentiated AC (A), and to a lower extent in moderately differentiated AC (B). SCC are mostly negative for AQP4, but have AQP4-positive intra-tumoral alveolar cells (C). D shows AQP4 expression in a pleomorphic carcinoma. Magnification ×20.

**Figure 4:**
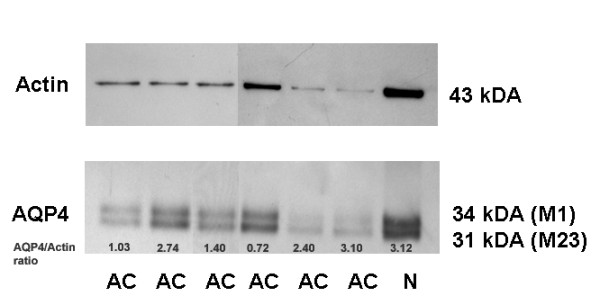
**Western blot analyses of AQP4 in normal lung and adenocarcinomas**. This western blot clearly demonstrates the expression of both the M1 (34 kDa) and the array-forming M23 variant (31 kDa) of AQP4 in normal lung (N) and adenocarcinomas (AC).

Since AQP4 expression has not been described previously in NSCLCs we aimed to further confirm the expression on the protein level. Anti-AQP4 immunoreactivity was found in 49 out of 125 NSCLC samples (39.2%). The analyses confirmed the transcript level that AQP4 protein expression was almost exclusively present in AC (Figure 2). The highest expression levels were seen in well-differentiated ACs, often with bronchioloalveolar and acinar differentiation, whereas poorly differentiated ACs, SCCs and the other investigated NSCLC subtypes showed no or only low-level AQP4 expression (Figure 3). Semi-quantitative analyses of AQP4 expression in ACs resulted in immunoreactivity scores (IRS) between 0 and 12 with a median IRS of 3 (Additional file 5, Table S5). There were no obvious differences between central and peripheral tumor areas. Western blotting displayed a similar expression level of the AQP4 isoforms M1 and M23 in AC and normal lung (Figure 4). AQP4 expression could not be detected in endothelial cells of pulmonary vessels.

Although Kaplan-Meier survival analyses of the anti-AQP4 IRS of the AC patients also indicated that AQP4 expression is associated with a better prognosis, the analyses failed statistical significance (AQP4 absent (IRS 0) vs. AQP4 present (IRS 1-12): p = 0.153; not shown). Notably, at the point of this study all AC patients with high AQP4 expression (IRS 9-12; n = 6) were still alive whereas only ~50% of the patients without AQP4 expression (IRS 0; n = 11) were alive (Additional file 5, Table S5). Thus, both the higher transcript and the protein level of AQP4 indicated a better prognosis in AC patients.

### Analyses of AQP4-coexpressed genes reveal an association to basic lung functions and respiratory disorders

**Figure 5:**
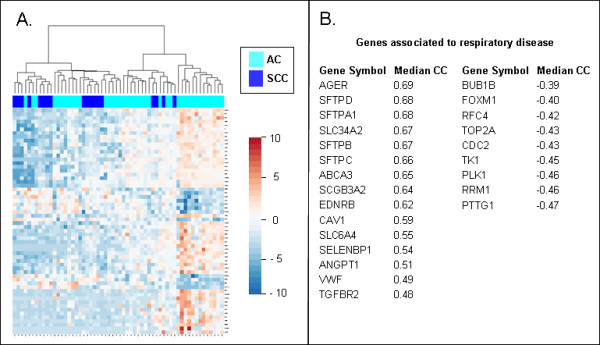
**AQP4-associated gene signature in NSCLC**. Supervised clustering of a gene set commonly correlated or anti-correlated to AQP4 gene expression indicates stronger expression in an AC subset (A). Several genes were annotated to be associated with respiratory disease by using Ingenuity software. Median correlation coefficient (CC) across independent microarray datasets was given for every gene (B).

Since both the expression and functional effects of AQP4 in NSCLC are unknown, we used five independent microarray datasets to identify potential gene-gene interactions and common biological processes correlated to AQP4 expression. The analysis of 593 non-redundant (out of 1000 best ranked) genes resulted in 140 genes (24%), which were present in at least two independent datasets (Additional file 3, Table S3). Concerning the fact that four different microarray platforms and three different specimen groups (AC, SCC, normal) were integrated we concluded a good concordance in this correlation analysis. The visualization of commonly AQP4-associated genes in one dataset [21] revealed upregulation of these genes mainly in an AC subgroup (Figure 5A), which was in agreement with the qRT-PCR results. Data mining of the gene set for the potential relevant functions and correlating diseases revealed major associations of AQP4 to cancer and respiratory disease, as well as links to biological processes like cell cycle regulation, lipid metabolism, cellular movement and cell-to-cell signalling (Table 3). Many of these genes were involved in specific lung functions like the maintenance of pulmonary surfactant, surface tension, and homeostasis (e.g. pulmonary-associated surfactant protein family). A subset of genes co-expressed with AQP4 and associated to respiratory disease were assigned to potential anti-tumorigenic function like CAV1 [28], EDNRB [29], or SELENBP1 [30], whereas genes more related to pro-tumorigenic function like CDK2 [31], FOXM1 [32], PLK1 [33] or RRM1 [34] were found to be anti-correlated with AQP4 (Figure 5B). Thus, data mining of AQP4-coexpressed genes indicated important physiological and anti-tumorigenic functions in both the normal and tumor cells.

**Table 3:** Data mining of AQP4-correlated genes across five independent microarray datasets using commercial pathway database (Ingenuity Pathway Analysis)

Relevant Functions and Diseases	No. of genes	Examples (Gene Symbol)
Cancer	65	CALCRL, CDC2, CDH5, DMBT1, EZH2, FABP4, FOLR1, ROS1, THBS2

Respiratory Disease	25	ANGPT1, BUB1B, CAV1, EDNRB, PLK1, RRM1, TGFBR2, TOP2A, VWF

Cell Cycle	17	BUB1B, CDC45L, CDKN3, CENPF, CITED2, MYBL2, PTTG1, RPS6KA2

Lipid Metabolism	15	CAT, CAV1, CYP27A1, FABP4, LPL, SEPP1, SFTPA1, SFTPB, SFTPC, SFTPD

Molecular Transport	17	ABCA3, AGTR2, AQP1, CFD, CYP27A1 NME1

Small Molecule Biochemistry	30	A2M, AOC3, C7, CAT, DIO2, GPX3, LPL, NPR1, PFN2, SEPP1, TEK, TK1

Cellular Movement	35	CAT, CTSE, DLC1, ESAM, ICAM2, PLA2G1B, S1PR1, SFTPC, SFTPD, VIPR1

Cell-to-Cell Signalling	30	C4BPA, COL4A3, DPYSL2, PTPRB, SLC6A4, SULF1, THY1, TROAP

Annotation of 140 commonly AQP4-associated genes revealed strong associations to cancer and pulmonary functions. All entries obtained significant p-value < 0.05. Examples of genes from the input list were given for each function and disease ontology. Detailed information is given in Supplementary Table 3.

## Discussion

In the present study we provide a comprehensive overview of AQP expression in both normal lung and NSCLC specimens in order to determine potentially relevant AQPs for novel anti-cancer therapies. The analysis of 576 different microarray profiles from normal lung, AC and SCC and immunohistochemical data revealed abundant and most variable expression patterns for AQP1, -3, -4, and -5. Concerning the presence in normal lung tissues, our expression data of distinct AQP isoforms based on transcriptomic data were in agreement with previous protein data [13]. If AQPs were present in NSCLC, the expression was mainly increased in ACs (AQP1, -3 and -4). For AQP1 [14,15] and -3 [17] higher protein expression in AC compared to SCC has already been described and AQP3 was found to be predominantly expressed in better differentiated AC [17].

For the first time, we identified AQP4 expression (isoforms M1 and M23) in NSCLCs, especially in well differentiated ACs, which has not been described before. Expression was almost exclusively derived from AC cells. Minor expression of AQP4 mRNA detected in SCC was at least due to the remaining intra-tumoral alveolar cells as shown by IHC. Of note, higher expression of AQP4 in AC was associated to a better outcome and a similar trend was seen in Kaplan-Meier survival analyses based on the protein expression data. Data mining of co-expressed and anti-correlated genes depicted the association of AQP4 to physiological functions in normal lung tissue which may be progressively lost during tumor dedifferentiation. A subsequent loss of AQP4 during carcinogenesis is also described in gastric cancer and AQP4 was therefore suggested to be used as a marker for normal proliferating gastric cells [35]. In addition, high AQP4 expression levels are also detectable in well-differentiated low-grade gliomas [27] and there is indication that only the expression of AQP1 enhanced cell growth and migration of gliomas, while AQP4 expression enhanced cell adhesion [36]. Thus, also AQP4 expression was investigated in various tumor entities, a significant pro-tumorigenic effect of this channel could not be identified so far. Since AQP4 expression could not be detected in endothelial cells this channel does not seem to play a role in tumor-associated edema formation and resolution. Yet, only AQP1 expression was identified in pulmonary endothelial cells and seems to facilitate hydostatically driven edema but was found not to be required for active near-isosmolar absorption of alveolar fluid under experimental conditions [37].

Previously, AQP1 [15], -3 [17], and -5 [18] have been suggested to be involved in NSCLC pathogenesis and pro-tumorigenic functions have been reported for AQP1 and AQP5 based on *in vitro *overexpression studies [15,18]. In our study, AQP4 expression can be assigned to physiological pulmonary functions and does not negatively correlate with the survival of NSCLC patients. Of note, a strong correlation between AQP1 and AQP4 expression was observed across more than 600 different lung specimens that indicate functional relations between both AQPs. This may also question sole pro-tumorigenic function of AQP1 in the human lung. An explanation may be that the long-term downregulation of AQPs in dedifferentiated tumor cells is the consequence of altered water and ion homeostasis or even an adaption mechanism against apoptosis, which is associated with low AQP expression levels [3], whereas overexpression of single AQPs in tumor cells may promote oncogenic functions such as cell migration, thereby contributing to tumor progression. Few in-vivo studies in other cancer entities demonstrated that targeted gene disruption of AQPs slows down cancer progression [7] or even prevents tumor formation [5]. Therefore, AQPs have been recognized as promising targets for novel anti-cancer therapies [12,38]. For NSCLC, AQP5 may be the most interesting candidate for novel anti-cancer therapies [18,19].

## Conclusions

In summary, we provide a basis to consider specific AQPs as potential drug targets in NSCLC. Notably, high AQP4 expression was found in pulmonary AC with higher differentiation and better prognosis, which challenges the therapeutic usage of this channel in particular. Our findings underline the importance to specify the potential functions of AQPs beyond water transport both in normal tissue and tumor cells.

## Competing interests

The authors declare that they have no competing interests.

## Authors' contributions

AW designed the study, participated in tissue microarray construction, immunohistochemical analyses, drafted the manuscript, read and approved the final version of the manuscript. TM and MM participated in tissue microarray construction, provided clinicopathological data, participated in banking of fresh-frozen specimens, read and approved the final version of the manuscript. EH participated in tissue microarray construction, banking of fresh-frozen specimens, read and approved the final version of the manuscript. AP performed and evaluated western blotting analyses, read and approved the final version of the manuscript. HH performed surgical resection of all used specimens, provided clinical data, read and approved the final version of the manuscript. PAS performed all histopathological diagnoses, participated in immunohistochemical analyses, read and approved the final version of the manuscript. CB and AB participated in statistical analyses, read and approved the final version of the manuscript. PS critically revised, read and approved the final version of the manuscript. RK designed the study, participated in microarray analyses, statistical analyses, drafted the manuscript, read and approved the final version of the manuscript.

## Pre-publication history

The pre-publication history for this paper can be accessed here:

http://www.biomedcentral.com/1471-2407/11/161/prepub

## Supplementary Material

Additional file 1 **Table S1. Integrated microarray datasets**. Overview about all integrated microarray datasets including references, microarray platform information, number of NSCLC (AC, SCC) and normal lung samples, and possible comparisons conducted in the expression analysis of aquaporins. Click here for file

Additional file 2 **Table S2. AQP gene expression patterns across five different microarray datasets**. AQPs were analyzed by comparing different sample sets like tumor (Tu) and normal (No) tissues as well as tumor subtypes adenocarcinoma (AC) and squamous cell carcinoma (SCC) including representative microarray features, p-value (Student's t-Test) and fold change in every dataset. The values in bold indicate significantly differential expression (p-value < 0.05; Fold change > 2 or < 0.5). Fields marked by a cross means that the AQP is not present in the dataset or the comparison could not performed because of absence of distinct sample sets. Relative expression values of all AQP isoforms were ranked in dependence all present genes in each dataset, and the median value across all datasets indicates that a certain AQP is stronger expressed than a defined proportion (%) of all other genes. Click here for file

Additional file 3 **Table S3. AQP4 co-expressed and anti-correlated genes across five independent microarray datasets**. AQP4 co-expressed and anti-correlated genes across five independent microarray datasets measured by Pearson correlation. In total 593 non-redundant genes (out of 200 best ranked features of each microarray dataset) were extracted and ranked by the presence frequency (No. of datasets) and the median correlation coefficiency (CC) across different datasets. Genes included in at least two independent datasets were assigned to prominent cellular functions or diseases by Ingenuity Pathway Database software. Click here for file

Additional file 4 **Table S4. Validation data of AQP expression in normal lung and NSCLC by qRT-PCR**. The qRT-PCR (Taqman) data of normal lung (N) and NSCLC tumor (T) tissues are presented according to the delta-delta Ct method including raw data (Ct values and MAD error), normalized data by ESD housekeeping gene (dCt value and error) and expression values (ddCt, ddCt error, expression level, level error) relative to the median of the normal lung samples. In the case of the different lung cancer cell lines expression values are assigned relative to A549 cell line. Taqman assays of AQP and ESD genes are specified by gene symbol, assay ID and representative sequence. Click here for file

Additional file 5 **Table S5. Clinicopathologic data of the tissue microarray specimens**. Patient data including survival status, follow-up, AQP4 immunoreactivity score (IRS) and tumor staging. Click here for file
