# Supplementary material for: Loss of aquaporin-4 expression and putative function in non-small cell lung cancer
Source: BMC Cancer. 2011 May 6;11:161. doi: 10.1186/1471-2407-11-161 (PMC3098822; doi:10.1186/1471-2407-11-161)
Supplement: Additional file 1 — Table S1. Integrated microarray datasets. Overview about all integrated microarray datasets including references, microarray platform information, number of NSCLC (AC, SCC) and normal lung samples, and possible comparisons conducted in the expression analysis of aquaporins. [file 1471-2407-11-161-S1.PDF]

Supplemental Table 1: Overview about all integrated microarray datasets including references, microarray platform information, number of NSCLC (AC, SCC) and normal lung samples, and possible comparisons conducted in the expression analysis of aquaporins.

**Microarray study**	**Citation; Accession No**	**Platform**	**n (AC)**	**n (SCC)**	**n (Normal)**	**Tumor vs Normal**	**AC vs SCC**
Kuner et al. 2008	[21]; NCBI GSE10245	Affy U133 Plus 2.0	40	18	0	no	yes
Kim et al. 2007	[25]; NCBI GSE8894	Affy U133 Plus 2.0	62	76	0	no	yes
Beer et al. 2002	[22]	Affy HU6800	86	0	10	yes	no
Garber et al. 2001	[24]; NCBI GSE3398	Spotted cDNA	39	13	5	yes	yes
Bhattacharjee et al. 2001	[23]	Affy U95A	190	20	17	yes	yes
**Total**	5 Datasets	4 Platforms	417	127	32	3 Datasets	4 Datasets
