# Supplementary material for: Loss of aquaporin-4 expression and putative function in non-small cell lung cancer
Source: BMC Cancer. 2011 May 6;11:161. doi: 10.1186/1471-2407-11-161 (PMC3098822; doi:10.1186/1471-2407-11-161)
Supplement: Additional file 2 — Table S2. AQP gene expression patterns across five different microarray datasets. AQPs were analyzed by comparing different sample sets like tumor (Tu) and normal (No) tissues as well as tumor subtypes adenocarcinoma (AC) and squamous cell carcinoma (SCC) including representative microarray features, p-value (Student's t-Test) and fold change in every dataset. The values in bold indicate significantly differential expression (p-value < 0.05; Fold change > 2 or < 0.5). Fields marked by a cross means that the AQP is not present in the dataset or the comparison could not performed because of absence of distinct sample sets. Relative expression values of all AQP isoforms were ranked in dependence all present genes in each dataset, and the median value across all datasets indicates that a certain AQP is stronger expressed than a defined proportion (%) of all other genes. [file 1471-2407-11-161-S2.PDF]

Supplemental Table 2: AQP gene expression patterns across five different microarray datasets. AQPs were analyzed by comparing different sample sets like tumor (Tu) and normal (No) tissues as well as tumor subtypes adenocarcinoma (AC) and squamous cell carcinoma (SCC) including representative microarray features, p-value (Student's t-Test) and fold change in every dataset. The values in bold indicate significantly differential expression (p-value < 0.05; Fold change > 2 or < 0.5). Fields marked by a cross means that the AQP is not present in the dataset or the comparison could not performed because of absence of distinct sample sets. Relative expression values of all AQP isoforms were ranked in dependence all present genes in each dataset, and the median value across all datasets indicates that a certain AQP is stronger expressed than a defined proportion (%) of all other genes.

Gene Symbol	Bhattacharjee, 2001				Garber, 2001				Beer, 2002				Kim, 2007				Kuner, 2008				Relative Expression (Median value > % of all measured genes)
	Probe_ID	Test	p-value	Fold change	Probe_ID	Test	p-value	Fold change	Probe_ID	Test	p-value	Fold change	Probe_ID	Test	p-value	Fold change	Probe_ID	Test	p-value	Fold change	
AQP1	36156_at	Tu vs No	***0.0000***	***0.17***	H23036	Tu vs No	***0.0000***	***0.28***	M77829_s_at, U41518_at	Tu vs No	***0.0000***	***0.18***	207542_s_at, 209047_at	Tu vs No	x	x	207542_s_at, 209047_at	Tu vs No	x	x	88
		AC vs SCC	0.0000	1.39		AC vs SCC	***0.0002***	***3.42***		AC vs SCC	x	x		AC vs SCC	***0.0000***	***11.57***		AC vs SCC	0.0087	1.73	
AQP2	39679_at	Tu vs No	0.0029	1.09	AA910952, AI075049	Tu vs No	0.0213	1.51	D31846_at	Tu vs No	0.0006	1.21	236630_at, 206672_at, 240285_at	Tu vs No	x	x	236630_at, 206672_at, 240285_at	Tu vs No	x	x	72
		AC vs SCC	0.1271	1.02		AC vs SCC	0.2658	1.14		AC vs SCC	x	x		AC vs SCC	0.4454	1.00		AC vs SCC	0.1597	1.00	
AQP3	39248_at, 39249_at	Tu vs No	0.2281	1.00	R91904	Tu vs No	0.0155	0.77	AB001325_at	Tu vs No	0.0001	1.11	39248_at, 39249_at, 203747_at	Tu vs No	x	x	39248_at, 39249_at, 203747_at	Tu vs No	x	x	87
		AC vs SCC	0.2765	1.39		AC vs SCC	***0.0005***	***4.79***		AC vs SCC	x	x		AC vs SCC	***0.0000***	***4.32***		AC vs SCC	***0.0000***	***8.18***	
AQP4	40793_s_at	Tu vs No	0.0000	0.63	H09087, N46843	Tu vs No	***0.0000***	***0.10***	U34844_at	Tu vs No	***0.0001***	***0.22***	210068_s_at, 210906_x_at, 226228_at	Tu vs No	x	x	210068_s_at, 210906_x_at, 226228_at	Tu vs No	x	x	58
		AC vs SCC	0.0003	1.09		AC vs SCC	***0.0228***	***2.42***		AC vs SCC	x	x		AC vs SCC	***0.0001***	***6.19***		AC vs SCC	***0.0258***	***2.77***	
AQP5	32909_at	Tu vs No	0.0418	0.94	AA496539	Tu vs No	0.0019	0.80	U46569_at	Tu vs No	0.0016	0.99	213611_at	Tu vs No	x	x	213611_at	Tu vs No	x	x	61
		AC vs SCC	0.0009	1.09		AC vs SCC	0.1574	1.13		AC vs SCC	x	x		AC vs SCC	***0.0000***	***6.93***		AC vs SCC	0.0094	1.00	
AQP6	32423_at	Tu vs No	0.0206	1.04	x	Tu vs No	x	x	U48408_at	Tu vs No	0.8605	0.91	208435_s_at, 216219_at	Tu vs No	x	x	208435_s_at, 216219_at	Tu vs No	x	x	48
		AC vs SCC	0.1672	1.04		AC vs SCC	x	x		AC vs SCC	x	x		AC vs SCC	0.2469	1.01		AC vs SCC	0.4315	1.00	
AQP7	39567_at, 39568_g_at	Tu vs No	0.1167	1.03	AI668576, AI732264, H27752	Tu vs No	0.4270	0.76	AB006190_at	Tu vs No	0.3480	1.01	206955_at	Tu vs No	x	x	206955_at	Tu vs No	x	x	50
		AC vs SCC	0.1704	1.04		AC vs SCC	0.2601	1.32		AC vs SCC	x	x		AC vs SCC	0.0065	1.03		AC vs SCC	0.0080	1.02	
AQP8	33651_at	Tu vs No	0.0889	0.97	x	Tu vs No	x	x	x	Tu vs No	x	x	206784_at	Tu vs No	x	x	206784_at	Tu vs No	x	x	2
		AC vs SCC	0.7616	0.98		AC vs SCC	x	x		AC vs SCC	x	x		AC vs SCC	0.0586	1.00		AC vs SCC	0.8800	1.00	
AQP9	34435_at	Tu vs No	0.0000	0.78	AA621132	Tu vs No	***0.0060***	***0.26***	x	Tu vs No	x	x	205568_at	Tu vs No	x	x	205568_at	Tu vs No	x	x	70
		AC vs SCC	0.3531	1.01		AC vs SCC	0.4136	0.80		AC vs SCC	x	x		AC vs SCC	0.1295	1.46		AC vs SCC	0.5567	0.62	
AQP10	x	Tu vs No	x	x	x	Tu vs No	x	x	x	Tu vs No	x	x	1555338_s_at	Tu vs No	x	x	1555338_s_at	Tu vs No	x	x	8
		AC vs SCC	x	x		AC vs SCC	x	x		AC vs SCC	x	x		AC vs SCC	0.7993	1.00		AC vs SCC	0.1473	0.99	
AQP11	x	Tu vs No	x	x	x	Tu vs No	x	x	x	Tu vs No	x	x	229526_at	Tu vs No	x	x	229526_at	Tu vs No	x	x	18
		AC vs SCC	x	x		AC vs SCC	x	x		AC vs SCC	x	x		AC vs SCC	0.0074	1.01		AC vs SCC	0.4195	1.00	
AQP12A	x	Tu vs No	x	x	x	Tu vs No	x	x	x	Tu vs No	x	x	1554344_s_at	Tu vs No	x	x	1554344_s_at	Tu vs No	x	x	29
		AC vs SCC	x	x		AC vs SCC	x	x		AC vs SCC	x	x		AC vs SCC	0.3008	1.02		AC vs SCC	0.2022	1.01	
AQP12B	x	Tu vs No	x	x	x	Tu vs No	x	x	x	Tu vs No	x	x	1559575_a_at	Tu vs No	x	x	1559575_a_at	Tu vs No	x	x	6
		AC vs SCC	x	x		AC vs SCC	x	x		AC vs SCC	x	x		AC vs SCC	0.5447	1.00		AC vs SCC	0.9883	1.00	
