# Supplementary material for: Loss of aquaporin-4 expression and putative function in non-small cell lung cancer
Source: BMC Cancer. 2011 May 6;11:161. doi: 10.1186/1471-2407-11-161 (PMC3098822; doi:10.1186/1471-2407-11-161)
Supplement: Additional file 3 — Table S3. AQP4 co-expressed and anti-correlated genes across five independent microarray datasets. AQP4 co-expressed and anti-correlated genes across five independent microarray datasets measured by Pearson correlation. In total 593 non-redundant genes (out of 200 best ranked features of each microarray dataset) were extracted and ranked by the presence frequency (No. of datasets) and the median correlation coefficiency (CC) across different datasets. Genes included in at least two independent datasets were assigned to prominent cellular functions or diseases by Ingenuity Pathway Database software. [file 1471-2407-11-161-S3.PDF]

Supplemental Table 3: AQP4 co-expressed and anti-correlated genes across five independent microarray datasets measured by Pearson correlation. In total 593 non-redundant genes (out of 200 best ranked features of each microarray dataset) were extracted and ranked by the presence frequency (No. of datasets) and the median correlation coefficiency (CC) across different datasets. Genes included in at least two independent datasets were assigned to prominent cellular functions or diseases by Ingenuity Pathway Database software.

No.	Gene Symbol	NCBI GeneID	No. Of datasets	Median CC	Beer ProbeID	Beer CC	Bhattacharjee ProbeID	Bhattacharjee CC	Garber ProbeID	Garber CC	Kim ProbeID	Kim CC	Kuner ProbeID	Kuner CC	Cancer	Respiratory Disease	Cell Cycle	Lipid Metabolism	Molecular Transport	Small Molecule Biochemistry	Cellular Movement	Cell-to-Cell Signaling
1	AQP4	361	5	0.94	D63412_at	1.00	40793_s_at	1.00	H09087	0.89	210906_x_at	0.92	210068_s_at	0.94					x			
2	AGER	177	5	0.69	U89336_cds3_at	0.74	35868_at	0.61	W74536	0.80	210081_at	0.64	210081_at	0.69	x	x				x	x	x
3	SFTPD	6441	5	0.68	HG2809-HT2920_s_at	0.68	31775_at	0.68	AI732255	0.61	214199_at	0.75	214199_at	0.78	x	x		x	x	x	x	x
4	SFTPC	6440	5	0.66	J03890_rna1_at	0.66	38691_s_at	0.61	AA487571	0.62	205982_x_at	0.70	214387_x_at	0.77		x		x	x	x	x	
5	ABCA3	21	5	0.65	U78735_at	0.61	35183_at	0.63	H29520	0.65	204343_at	0.70	204343_at	0.78		x		x	x	x		
6	AQP1	358	4	0.68	NA	NA	36156_at	0.46	H23036	0.73	209047_at	0.62	207542_s_at	0.80	x				x			
7	CLIC5	53405	4	0.68	NA	NA	34194_at	0.57	R82801	0.75	213317_at	0.68	213317_at	0.67	x							
8	CLDN18	51208	4	0.65	NA	NA	38239_at	0.57	N70059	0.72	214135_at	0.60	214135_at	0.69								
9	GPR116	221395	4	0.64	NA	NA	34235_at	0.62	N95226	0.65	212951_at	0.61	212951_at	0.71								
10	EDNRB	1910	4	0.62	D13168_at	0.56	1198_at	0.46	H28710	0.72	NA	NA	206701_x_at	0.69	x	x				x	x	
11	ADH1B	125	4	0.62	X03350_at	0.56	35730_at	0.56	N93428	0.77	NA	NA	209614_at	0.69	x							
12	AOC3	8639	4	0.61	U39447_at	0.57	33756_at	0.49	AA036974	0.70	NA	NA	205654_at	0.65	x					x	x	x
13	CLEC3B	7123	4	0.61	X64559_at	0.63	36569_at	0.55	W73889	0.67	205200_at	0.58	NA	NA	x							
14	SLC39A8	64116	3	0.71	NA	NA	40456_at	0.71	NA	NA	209267_s_at	0.63	209267_s_at	0.72								
15	C4orf31	79625	3	0.68	NA	NA	NA	NA	AA010797	0.68	230319_at	0.71	219747_at	0.66								
16	SFTPA1	6435	3	0.68	M13686_s_at	0.57	NA	NA	NA	NA	223678_s_at	0.68	223678_s_at	0.78		x		x	x	x		x
17	SLC34A2	10568	3	0.67	NA	NA	NA	NA	AA459296	0.67	204124_at	0.66	204124_at	0.70	x	x						
18	SFTPB	6439	3	0.67	NA	NA	33383_f_at	0.58	NA	NA	213936_x_at	0.67	214354_x_at	0.69		x		x	x	x		
19	CYP4B1	1580	3	0.65	J02871_s_at	0.65	NA	NA	NA	NA	210096_at	0.58	210096_at	0.68								
20	TNNC1	7134	3	0.64	M37984_rna1_at	0.73	NA	NA	NA	NA	209904_at	0.59	209904_at	0.64								
21	SFTPA2	6436	3	0.63	HG3925-HT4195_s_at	0.52	NA	NA	NA	NA	218835_at	0.63	218835_at	0.72								
22	LMO3	4006	3	0.63	NA	NA	38028_at	0.51	NA	NA	204424_s_at	0.63	204424_s_at	0.64	x							
23	ALPL	249	3	0.62	NA	NA	36623_at	0.52	AA873885	0.62	NA	NA	215783_s_at	0.64	x							
24	EMP2	2013	3	0.61	U52100_at	0.61	39631_at	0.56	T57082	0.69	NA	NA	NA	NA	x							
25	DLC1	10395	3	0.61	NA	NA	37951_at	0.61	NA	NA	210762_s_at	0.59	210762_s_at	0.65	x						x	
26	FOLR1	2348	3	0.61	NA	NA	534_s_at	0.46	R24530	0.64	204437_s_at	0.61	NA	NA	x					x		
27	CYB5A	1528	3	0.61	NA	NA	38459_g_at	0.56	R91950	0.61	NA	NA	215726_s_at	0.63								
28	LPL	4023	3	0.58	NA	NA	41209_at	0.49	AA633835	0.65	203548_s_at	0.58	NA	NA				x	x	x		x
29	PTPRB	5787	3	0.58	X54131_at	0.60	37777_at	0.52	NA	NA	230250_at	0.58	NA	NA								x
30	KIAA0256	9728	3	0.57	NA	NA	41634_at	0.54	AA633872	0.65	212450_at	0.57	NA	NA								
31	RPS6KA2	6196	3	0.56	NA	NA	33325_at	0.49	AA447506	0.64	212912_at	0.56	NA	NA			x					
32	CACNA2D2	9254	3	0.56	NA	NA	37811_at	0.55	N53512	0.62	204811_s_at	0.56	NA	NA	x							
33	CD302	9936	3	0.55	D14664_at	0.48	34760_at	0.55	H60460	0.70	NA	NA	NA	NA								
34	PECAM1	5175	3	0.54	L34657_at	0.51	37397_at	0.54	R22412	0.60	NA	NA	NA	NA	x						x	x
35	S1PR1	1901	3	0.52	M31210_at	0.52	587_at	0.48	R13546	0.70	NA	NA	NA	NA	x		x			x	x	x
36	TEK	7010	3	0.49	L06139_at	0.49	1596_g_at	0.49	H02848	0.65	NA	NA	NA	NA	x			x	x	x	x	x
37	PLA2G1B	5319	3	0.46	M21056_at	0.46	912_s_at	0.46	NA	NA	NA	NA	206311_s_at	0.67							x	
38	TROAP	10024	3	-0.35	U04810_at	-0.35	32702_at	-0.33	H94949	-0.50	NA	NA	NA	NA								x
39	GAPDH	2597	3	-0.40	X01677_f_at	-0.40	35905_s_at	-0.39	NA	NA	NA	NA	M33197_3_at	-0.50		x						
40	PFN2	5217	3	-0.49	NA	NA	NA	NA	AA040703	-0.49	204992_s_at	-0.46	204992_s_at	-0.51						x		
41	LRRK2	120892	2	0.78	NA	NA	NA	NA	NA	NA	229584_at	0.73	229584_at	0.83								
42	PEBP4	157310	2	0.74	NA	NA	NA	NA	NA	NA	227848_at	0.64	227848_at	0.84								
43	NRGN	4900	2	0.74	NA	NA	NA	NA	NA	NA	204081_at	0.66	204081_at	0.81								
44	C4BPA	722	2	0.68	NA	NA	NA	NA	T62036	0.70	205654_at	0.67	NA	NA	x							x
45	LAMP3	27074	2	0.67	NA	NA	37168_at	0.66	NA	NA	NA	NA	205569_at	0.69								
46	AMY1A	276	2	0.67	NA	NA	NA	NA	NA	NA	208498_s_at	0.59	208498_s_at	0.74								
47	CTSE	1510	2	0.66	NA	NA	NA	NA	NA	NA	205927_s_at	0.65	205927_s_at	0.68							x	
48	NAPSA	9476	2	0.66	NA	NA	NA	NA	NA	NA	223806_s_at	0.67	223806_s_at	0.66								
49	CRTAC1	55118	2	0.66	NA	NA	NA	NA	NA	NA	221204_s_at	0.59	221204_s_at	0.73								
50	SUSD2	56241	2	0.66	NA	NA	NA	NA	NA	NA	227480_at	0.58	227480_at	0.73								
51	LOC253970	253970	2	0.65	NA	NA	NA	NA	N80764	0.61	228979_at	0.69	NA	NA								
52	VEPH1	79674	2	0.64	NA	NA	NA	NA	NA	NA	232122_s_at	0.61	232122_s_at	0.68								
53	SCGB3A2	117156	2	0.64	NA	NA	NA	NA	NA	NA	228782_at	0.60	228782_at	0.67		x						
54	ESAM	90952	2	0.63	NA	NA	NA	NA	NA	NA	225369_at	0.58	225369_at	0.68							x	x
55	CLEC14A	161198	2	0.63	NA	NA	NA	NA	NA	NA	226244_at	0.58	226244_at	0.67								
56	ROS1	6098	2	0.63	M34353_s_at	0.50	NA	NA	NA	NA	NA	NA	207569_at	0.75	x							
57	FCN3	8547	2	0.63	NA	NA	34708_at	0.56	NA	NA	NA	NA	205866_at	0.70								
58	LOC286189	286189	2	0.62	NA	NA	NA	NA	NA	NA	1556421_at	0.56	1556421_at	0.69								
59	PAPSS2	9060	2	0.61	NA	NA	NA	NA	R99758	0.66	203060_s_at	0.57	NA	NA	x							
60	AGR3	155465	2	0.61	NA	NA	NA	NA	NA	NA	228241_at	0.57	228241_at	0.66	x							
61	CPM	1368	2	0.61	NA	NA	NA	NA	T49654	0.64	235019_at	0.59	NA	NA								
62	HSD17B6	8630	2	0.61	NA	NA	37512_at	0.53	AA034944	0.69	NA	NA	NA	NA	x							
63	NAPSB	256236	2	0.60	NA	NA	NA	NA	NA	NA	228055_at	0.57	228055_at	0.63								
64	CAV1	857	2	0.59	Z18951_at	0.65	36119_at	0.54	NA	NA	NA	NA	NA	NA	x	x	x	x	x	x	x	x
65	CSPG5	10675	2	0.59	NA	NA	39966_at	0.48	NA	NA	NA	NA	39966_at	0.70								
66	C7	730	2	0.58	NA	NA	37394_at	0.54	NA	NA	NA	NA	202992_at	0.63						x	x	
67	LDB2	9079	2	0.58	NA	NA	36065_at	0.47	H73914	0.70	NA	NA	NA	NA								
68	STEAP4	79689	2	0.58	NA	NA	NA	NA	N30680	0.61	225987_at	0.55	NA	NA								
69	DPYSL2	1808	2	0.57	NA	NA	40607_at	0.54	AA487460	0.61	NA	NA	NA	NA	x						x	x
70	AGTR2	186	2	0.57	NA	NA	33496_at	0.47	NA	NA	NA	NA	207294_at	0.68				x	x	x	x	
71	CYP27A1	1593	2	0.56	X59812_at	0.53	NA	NA	N66957	0.60	NA	NA	NA	NA				x	x	x		
72	CAPN3	825	2	0.56	NA	NA	39301_at	0.47	NA	NA	NA	NA	210944_s_at	0.65	x							
73	RASGRF1	5923	2	0.56	NA	NA	31949_at	0.48	NA	NA	NA	NA	210550_s_at	0.64								
74	CITED2	10370	2	0.55	NA	NA	33113_at	0.54	NA	NA	209357_at	0.57	NA	NA								
75	SPTBN1	6711	2	0.55	M96803_at	0.51	NA	NA	AA018591	0.60	NA	NA	NA	NA	x							
76	LIMCH1	22998	2	0.55	NA	NA	32812_at	0.55	NA	NA	212327_at	0.56	NA	NA								
77	ITGA8	8516	2	0.55	L36531_at	0.47	NA	NA	R87964	0.63	NA	NA	NA	NA								
78	SLC6A4	6532	2	0.55	L05568_at	0.46	NA	NA	AI261489	0.64	NA	NA	NA	NA	x	x			x	x		x
79	FHL1	2273	2	0.55	U60115_at	0.59	32542_at	0.50	NA	NA	NA	NA	NA	NA								
80	VIPR1	7433	2	0.55	X77777_s_at	0.47	NA	NA	H73241	0.62	NA	NA	NA	NA	x						x	x
81	SELENBP1	8991	2	0.54	NA	NA	37405_at	0.48	NA	NA	214433_s_at	0.60	NA	NA		x						
82	FRY	10129	2	0.54	NA	NA	1529_at	0.47	H09111	0.61	NA	NA	NA	NA	x							
83	A2M	2	2	0.54	M11313_s_at	0.45	NA	NA	AA775447	0.62	NA	NA	NA	NA				x	x	x		x
84	FOXF1	2294	2	0.54	U13219_at	0.61	38183_at	0.46	NA	NA	NA	NA	NA	NA	x						x	
85	CPA3	1359	2	0.53	M73720_at	0.56	36749_at	0.50	NA	NA	NA	NA	NA	NA								

No.	Gene Symbol	NCBI GeneID	No. Of datasets	Median CC	Beer ProbeID	Beer CC	Bhattacharjee ProbeID	Bhattacharjee CC	Garber ProbeID	Garber CC	Kim ProbeID	Kim CC	Kuner ProbeID	Kuner CC	Cancer	Respiratory Disease	Cell Cycle	Lipid Metabolism	Molecular Transport	Small Molecule Biochemistry	Cellular Movement	Cell-to-Cell Signaling
86	DMBT1	1755	2	0.53	NA	NA	41382_at	0.48	NA	NA	208250_s_at	0.58	NA	NA	x							
87	COL4A3	1285	2	0.53	NA	NA	33561_at	0.50	NA	NA	222073_at	0.56	NA	NA	x						x	X
88	GPX3	2878	2	0.53	D00632_at	0.57	770_at	0.48	NA	NA	NA	NA	NA	NA	x					x		
89	CALCRL	10203	2	0.53	L76380_at	0.60	34995_at	0.45	NA	NA	NA	NA	NA	NA	x						x	X
90	SEPP1	6414	2	0.52	Z11793_at	0.54	34363_at	0.50	NA	NA	NA	NA	NA	NA				x		x		
91	ANGPT1	284	2	0.51	D13628_at	0.51	1929_at	0.51	NA	NA	NA	NA	NA	NA	x	X	x			x	x	X
92	FBP1	2203	2	0.51	NA	NA	36495_at	0.46	NA	NA	209696_at	0.56	NA	NA								
93	SPARCL1	8404	2	0.50	X86693_at	0.50	36627_at	0.51	NA	NA	NA	NA	NA	NA								
94	CDH5	1003	2	0.50	X79981_at	0.49	37196_at	0.51	NA	NA	NA	NA	NA	NA	x						X	X
95	FABP4	2167	2	0.50	J02874_at	0.53	38430_at	0.47	NA	NA	NA	NA	NA	NA	x			x	x	x	x	
96	CAT	847	2	0.50	X04085_rna1_at	0.46	37009_at	0.54	NA	NA	NA	NA	NA	NA	x			x		x	x	x
97	P2RY14	9934	2	0.49	D13626_at	0.52	33462_at	0.46	NA	NA	NA	NA	NA	NA								
98	VWF	7450	2	0.49	M10321_s_at	0.47	607_s_at	0.51	NA	NA	NA	NA	NA	NA	x	x					x	x
99	TGFBR2	7048	2	0.48	D50683_at	0.46	1814_at	0.50	NA	NA	NA	NA	NA	NA	x	x					X	x
100	CFD	1675	2	0.48	M84526_at	0.49	40282_s_at	0.47	NA	NA	NA	NA	NA	NA	x			x	x	x		
101	ICAM2	3384	2	0.47	M32334_at	0.46	590_at	0.48	NA	NA	NA	NA	NA	NA							x	x
102	NPR1	4881	2	0.46	X15357_at	0.45	32625_at	0.46	NA	NA	NA	NA	NA	NA						X		X
103	TNXA	7146	2	0.45	U24488_s_at	0.45	38508_s_at	0.45	NA	NA	NA	NA	NA	NA								
104	THBS2	7058	2	-0.33	L12350_at	-0.34	659_g_at	-0.32	NA	NA	NA	NA	NA	NA	x						x	x
105	THY1	7070	2	-0.35	M11749_at	-0.37	39395_at	-0.33	NA	NA	NA	NA	NA	NA							x	x
106	NME1	4830	2	-0.36	X17620_at	-0.41	1521_at	-0.32	NA	NA	NA	NA	NA	NA	x				x	x	x	
107	PAFAH1B3	5050	2	-0.38	D63391_at	-0.33	35800_at	-0.42	NA	NA	NA	NA	NA	NA								
108	CDC45L	8318	2	-0.38	NA	NA	37458_at	-0.32	NA	NA	204126_s_at	-0.45	NA	NA	x							
109	BUB1B	701	2	-0.39	NA	NA	35699_at	-0.31	AA488324	-0.46	NA	NA	NA	NA	x	X	x					
110	EZH2	2146	2	-0.39	U61145_at	-0.36	37305_at	-0.42	NA	NA	NA	NA	NA	NA	x		x				x	
111	TRIP13	9319	2	-0.39	NA	NA	36813_at	-0.32	AA630784	-0.46	NA	NA	NA	NA	x							
112	CDKN3	1033	2	-0.39	NA	NA	1599_at	-0.31	AA284072	-0.47	NA	NA	NA	NA	x		x					
113	FOXM1	2305	2	-0.40	NA	NA	34715_at	-0.32	AA129552	-0.47	NA	NA	NA	NA	x	x	x				x	
114	MFAP2	4237	2	-0.40	U19718_at	-0.33	NA	NA	NA	NA	NA	NA	203417_at	-0.47								
115	COL10A1	1300	2	-0.40	NA	NA	38566_at	-0.33	NA	NA	NA	NA	217428_s_at	-0.48								
116	MXRA5	25878	2	-0.40	NA	NA	36861_at	-0.33	NA	NA	NA	NA	209596_at	-0.48	x							
117	TPBG	7162	2	-0.41	Z29083_at	-0.35	NA	NA	NA	NA	NA	NA	203476_at	-0.46							x	
118	SNRPB	6628	2	-0.41	X17567_s_at	-0.32	NA	NA	AA599116	-0.50	NA	NA	NA	NA								
119	RFC4	5984	2	-0.42	NA	NA	1055_g_at	-0.35	N93924	-0.49	NA	NA	NA	NA	x	X						
120	SHMT2	6472	2	-0.43	NA	NA	36178_at	-0.34	AA620477	-0.51	NA	NA	NA	NA								
121	PKMYT1	9088	2	-0.43	NA	NA	37238_s_at	-0.40	AA478066	-0.46	NA	NA	NA	NA	x		x					
122	UCK2	7371	2	-0.43	NA	NA	37193_at	-0.39	W69906	-0.47	NA	NA	NA	NA								
123	TOP2A	7153	2	-0.43	NA	NA	40145_at	-0.33	AA504348	-0.54	NA	NA	NA	NA	x	x	x					
124	CDC2	983	2	-0.43	NA	NA	1803_at	-0.34	AA598974	-0.53	NA	NA	NA	NA	x	X	x				X	
125	SULF1	23213	2	-0.43	NA	NA	35832_at	-0.38	NA	NA	NA	NA	212344_at	-0.49	x		X				X	x
126	MRPS12	6183	2	-0.44	NA	NA	33215_g_at	-0.35	R23752	-0.52	NA	NA	NA	NA								
127	PLOD2	5352	2	-0.44	U84573_at	-0.37	NA	NA	NA	NA	NA	NA	202619_s_at	-0.51	x							
128	CENPF	1063	2	-0.44	NA	NA	37302_at	-0.31	AA701455	-0.57	NA	NA	NA	NA	x		x					
129	DIO2	1734	2	-0.45	NA	NA	31902_at	-0.37	NA	NA	NA	NA	203700_s_at	-0.54						x		
130	NUDT1	4521	2	-0.45	NA	NA	35723_at	-0.36	AA443998	-0.55	NA	NA	NA	NA	x					x		
131	TK1	7083	2	-0.45	NA	NA	41400_at	-0.34	AA778098	-0.57	NA	NA	NA	NA	x	x				X		
132	MYBL2	4605	2	-0.46	NA	NA	1854_at	-0.36	AA456878	-0.55	NA	NA	NA	NA	x		x					X
133	PLK1	5347	2	-0.46	NA	NA	37228_at	-0.40	AA629262	-0.51	NA	NA	NA	NA	x	X	x					
134	RRM1	6240	2	-0.46	NA	NA	NA	NA	AA633549	-0.48	201477_s_at	-0.44	NA	NA	x	X						
135	PTTG1	9232	2	-0.47	NA	NA	40412_at	-0.34	AA430032	-0.59	NA	NA	NA	NA	x	X	x					
136	QSER1	79832	2	-0.47	NA	NA	NA	NA	AA452517	-0.46	NA	NA	226265_at	-0.47								
137	RMI1	80010	2	-0.47	NA	NA	NA	NA	N68390	-0.48	218979_at	-0.45	NA	NA	x		x					
138	MARK1	4139	2	-0.48	NA	NA	NA	NA	NA	NA	226653_at	-0.44	221047_s_at	-0.51								
139	NKIRAS2	28511	2	-0.48	NA	NA	NA	NA	R63172	-0.46	NA	NA	222105_s_at	-0.50								
140	COL11A1	1301	2	-0.50	NA	NA	37892_at	-0.38	NA	NA	NA	NA	229271_x_at	-0.62								
141	ATP13A4	84239	1	0.80	NA	NA	NA	NA	NA	NA	NA	NA	231512_at	0.80								
142	ZNF533	151126	1	0.75	NA	NA	NA	NA	NA	NA	NA	NA	1555801_s_at	0.75								
143	SDPR	8436	1	0.73	NA	NA	NA	NA	R09728	0.73	NA	NA	NA	NA								
144	C20orf133	140733	1	0.71	NA	NA	NA	NA	NA	NA	NA	NA	1563209_a_at	0.71								
145	HBA2	3040	1	0.69	Z84721_cds2_at	0.69	NA	NA	NA	NA	NA	NA	NA	NA								
146	ALOX15B	247	1	0.68	NA	NA	NA	NA	AA228916	0.68	NA	NA	NA	NA								
147	HBB	3043	1	0.67	HG1428-HT1428_s_at	0.67	NA	NA	NA	NA	NA	NA	NA	NA								
148	TTC17	55761	1	0.67	NA	NA	NA	NA	AA487501	0.67	NA	NA	NA	NA								
149	SYT15	83849	1	0.66	NA	NA	NA	NA	NA	NA	NA	NA	1560879_a_at	0.66								
150	PLLP	51090	1	0.66	NA	NA	NA	NA	R16034	0.66	NA	NA	NA	NA								
151	MGC14376	84981	1	0.66	NA	NA	NA	NA	AA454584	0.66	NA	NA	NA	NA								
152	SFTPG	207109	1	0.66	NA	NA	NA	NA	NA	NA	244056_at	0.66	NA	NA								
153	C9orf26	90865	1	0.65	NA	NA	NA	NA	NA	NA	NA	NA	209821_at	0.65								
154	MOSC2	54996	1	0.65	NA	NA	NA	NA	T63490	0.65	NA	NA	NA	NA								
155	AK1	203	1	0.65	NA	NA	NA	NA	NA	NA	NA	NA	202587_s_at	0.65								
156	GIMAP1	170575	1	0.65	NA	NA	NA	NA	NA	NA	NA	NA	1552318_at	0.65								
157	BTNL9	153579	1	0.64	NA	NA	NA	NA	NA	NA	NA	NA	228434_at	0.64								
158	C1orf116	79098	1	0.64	NA	NA	NA	NA	NA	NA	NA	NA	219476_at	0.64								
159	USP44	84101	1	0.64	NA	NA	NA	NA	NA	NA	NA	NA	224048_at	0.64								
160	TMPRSS2	7113	1	0.64	NA	NA	NA	NA	AA225817	0.64	NA	NA	NA	NA								
161	C9orf5	23731	1	0.64	NA	NA	NA	NA	NA	NA	NA	NA	223006_s_at	0.64								
162	SMAD6	4091	1	0.64	NA	NA	NA	NA	AA235597	0.64	NA	NA	NA	NA								
163	CMAH	8418	1	0.64	NA	NA	NA	NA	N29639	0.64	NA	NA	NA	NA								
164	FHL5	9457	1	0.64	NA	NA	NA	NA	NA	NA	NA	NA	220170_at	0.64								
165	KIAA1171	57465	1	0.64	NA	NA	NA	NA	NA	NA	NA	NA	227908_at	0.64								
166	ASAH1	427	1	0.64	NA	NA	NA	NA	AA664155	0.64	NA	NA	NA	NA								
167	IL6R	3570	1	0.64	NA	NA	NA	NA	NA	NA	NA	NA	205945_at	0.64								
168	TITF1	7080	1	0.64	NA	NA	NA	NA	NA	NA	231315_at	0.64	NA	NA								
169	DKFZP586A0	NA	1	0.63	NA	NA	NA	NA	T49984	0.63	NA	NA	NA	NA								
170	HSD17B4	3295	1	0.63	NA	NA	NA	NA	AA487914	0.63	NA	NA	NA	NA								

No.	Gene Symbol	NCBI GeneID	No. Of datasets	Median CC	Beer ProbeID	Beer CC	Bhattacharjee ProbeID	Bhattacharjee CC	Garber ProbeID	Garber CC	Kim ProbeID	Kim CC	Kuner ProbeID	Kuner CC	Cancer	Respiratory Disease	Cell Cycle	Lipid Metabolism	Molecular Transport	Small Molecule Biochemistry	Cellular Movement	Cell-to-Cell Signaling
171	FMO2	2327	1	0.63	Y09267_at	0.63	NA	NA	NA	NA	NA	NA	NA	NA								
172	MS4A7	58475	1	0.63	NA	NA	NA	NA	T62699	0.63	NA	NA	NA	NA								
173	MGP	4256	1	0.63	NA	NA	NA	NA	AA155913	0.63	NA	NA	NA	NA								
174	FCGRT	2217	1	0.63	NA	NA	NA	NA	T53508	0.63	NA	NA	NA	NA								
175	CXCL2	2920	1	0.63	NA	NA	NA	NA	R47771	0.63	NA	NA	NA	NA								
176	SLC5A9	200010	1	0.63	NA	NA	NA	NA	W78843	0.63	NA	NA	NA	NA								
177	TMEM100	55273	1	0.63	NA	NA	NA	NA	AA446661	0.63	NA	NA	NA	NA								
178	KIAA1462	57608	1	0.63	NA	NA	NA	NA	AA418988	0.63	NA	NA	NA	NA								
179	SPOCK2	9806	1	0.62	NA	NA	NA	NA	AA398230	0.62	NA	NA	NA	NA								
180	ERG	2078	1	0.62	NA	NA	NA	NA	R01192	0.62	NA	NA	NA	NA								
181	XLKD1	10894	1	0.62	NA	NA	NA	NA	H02823	0.62	NA	NA	NA	NA								
182	COLEC12	81035	1	0.62	NA	NA	NA	NA	N53421	0.62	NA	NA	NA	NA								
183	GKN2	200504	1	0.62	NA	NA	NA	NA	NA	NA	238222_at	0.62	NA	NA								
184	CXorf30	645090	1	0.62	NA	NA	NA	NA	AI822083	0.62	NA	NA	NA	NA								
185	IRF8	3394	1	0.62	NA	NA	NA	NA	N62269	0.62	NA	NA	NA	NA								
186	PRKCE	5581	1	0.62	NA	NA	NA	NA	H23225	0.62	NA	NA	NA	NA								
187	SCN7A	6332	1	0.61	NA	NA	NA	NA	AA453182	0.61	NA	NA	NA	NA								
188	EPAS1	2034	1	0.61	NA	NA	NA	NA	AA680300	0.61	NA	NA	NA	NA								
189	LOC285141	285141	1	0.61	NA	NA	NA	NA	AA887245	0.61	NA	NA	NA	NA								
190	FLRT3	23767	1	0.60	NA	NA	NA	NA	NA	NA	219250_s_at	0.60	NA	NA								
191	ALOX5	240	1	0.60	NA	NA	NA	NA	H50910	0.60	NA	NA	NA	NA								
192	FLJ14525	84886	1	0.60	NA	NA	NA	NA	N59373	0.60	NA	NA	NA	NA								
193	MSLN	10232	1	0.59	NA	NA	NA	NA	NA	NA	204885_s_at	0.59	NA	NA								
194	TMEM125	128218	1	0.59	NA	NA	NA	NA	NA	NA	225822_at	0.59	NA	NA								
195	DTR	1839	1	0.59	M60278_at	0.59	NA	NA	NA	NA	NA	NA	NA	NA								
196	TCF21	6943	1	0.59	NA	NA	37247_at	0.59	NA	NA	NA	NA	NA	NA								
197	FABP5	2171	1	0.58	M94856_at	0.58	NA	NA	NA	NA	NA	NA	NA	NA								
198	APM2	10974	1	0.58	D45370_at	0.58	NA	NA	NA	NA	NA	NA	NA	NA								
199	TGFBR3	7049	1	0.58	L07594_at	0.58	NA	NA	NA	NA	NA	NA	NA	NA								
200	MGC45438	146556	1	0.58	NA	NA	NA	NA	NA	NA	229177_at	0.58	NA	NA								
201	SYNE1	23345	1	0.57	NA	NA	38113_at	0.57	NA	NA	NA	NA	NA	NA								
202	ACADL	33	1	0.57	M74096_at	0.57	NA	NA	NA	NA	NA	NA	NA	NA								
203	MUC1	4582	1	0.57	NA	NA	NA	NA	NA	NA	213693_s_at	0.57	NA	NA								
204	HIGD1B	51751	1	0.57	NA	NA	NA	NA	NA	NA	219719_at	0.57	NA	NA								
205	NTN4	59277	1	0.56	NA	NA	NA	NA	NA	NA	223315_at	0.56	NA	NA								
206	GPM6A	2823	1	0.56	D49958_at	0.56	NA	NA	NA	NA	NA	NA	NA	NA								
207	GPRC5A	9052	1	0.56	NA	NA	NA	NA	NA	NA	203108_at	0.56	NA	NA								
208	KIAA0828	23382	1	0.56	NA	NA	NA	NA	NA	NA	212814_at	0.56	NA	NA								
209	KIAA0672	9912	1	0.56	NA	NA	NA	NA	NA	NA	205414_s_at	0.56	NA	NA								
210	ALDH3B1	221	1	0.56	NA	NA	NA	NA	NA	NA	205640_at	0.56	NA	NA								
211	FY	2532	1	0.56	X85785_rna1_at	0.56	NA	NA	NA	NA	NA	NA	NA	NA								
212	ST3GAL5	8869	1	0.55	NA	NA	NA	NA	NA	NA	203217_s_at	0.55	NA	NA								
213	SLC16A4	9122	1	0.55	NA	NA	NA	NA	NA	NA	205234_at	0.55	NA	NA								
214	KAL1	3730	1	0.55	M97252_at	0.55	NA	NA	NA	NA	NA	NA	NA	NA								
215	CADM1	23705	1	0.55	NA	NA	NA	NA	NA	NA	209032_s_at	0.55	NA	NA								
216	DKFZP564O0823	25849	1	0.55	NA	NA	NA	NA	NA	NA	225809_at	0.55	NA	NA								
217	PRKACA	5566	1	0.55	M80335_at	0.55	NA	NA	NA	NA	NA	NA	NA	NA								
218	DHCR24	1718	1	0.55	NA	NA	36658_at	0.55	NA	NA	NA	NA	NA	NA								
219	FGFR2	2263	1	0.54	M87770_at	0.54	NA	NA	NA	NA	NA	NA	NA	NA								
220	CTSH	1512	1	0.54	NA	NA	37021_at	0.54	NA	NA	NA	NA	NA	NA								
221	COX7A1	1346	1	0.53	M83186_at	0.53	NA	NA	NA	NA	NA	NA	NA	NA								
222	IL3RA	3563	1	0.52	D49410_at	0.52	NA	NA	NA	NA	NA	NA	NA	NA								
223	GNAQ	2776	1	0.52	U40038_at	0.52	NA	NA	NA	NA	NA	NA	NA	NA								
224	TNFSF13	8741	1	0.52	NA	NA	35287_at	0.52	NA	NA	NA	NA	NA	NA								
225	FGR	2268	1	0.52	M19722_at	0.52	NA	NA	NA	NA	NA	NA	NA	NA								
226	C7orf23	79161	1	0.51	NA	NA	41809_at	0.51	NA	NA	NA	NA	NA	NA								
227	DSCR1	1827	1	0.51	U28833_at	0.51	NA	NA	NA	NA	NA	NA	NA	NA								
228	STOM	2040	1	0.51	NA	NA	40419_at	0.51	NA	NA	NA	NA	NA	NA								
229	PDZD2	23037	1	0.50	NA	NA	40834_at	0.50	NA	NA	NA	NA	NA	NA								
230	GPA33	10223	1	0.50	U79725_at	0.50	NA	NA	NA	NA	NA	NA	NA	NA								
231	FIGF	2277	1	0.50	NA	NA	1958_at	0.50	NA	NA	NA	NA	NA	NA								
232	DEFA1	1667	1	0.50	M26602_at	0.50	NA	NA	NA	NA	NA	NA	NA	NA								
233	CD34	947	1	0.49	S53911_at	0.49	NA	NA	NA	NA	NA	NA	NA	NA								
234	TPSB1	7177	1	0.49	M33493_s_at	0.49	NA	NA	NA	NA	NA	NA	NA	NA								
235	SLPI	6590	1	0.49	X04470_s_at	0.49	NA	NA	NA	NA	NA	NA	NA	NA								
236	TIE1	7075	1	0.49	NA	NA	1001_at	0.49	NA	NA	NA	NA	NA	NA								
237	CLDN5	7122	1	0.49	NA	NA	38995_at	0.49	NA	NA	NA	NA	NA	NA								
238	DUSP1	1843	1	0.49	NA	NA	1005_at	0.49	NA	NA	NA	NA	NA	NA								
239	C9orf61	9413	1	0.49	NA	NA	32971_at	0.49	NA	NA	NA	NA	NA	NA								
240	NDST1	3340	1	0.49	U18932_at	0.49	NA	NA	NA	NA	NA	NA	NA	NA								
241	CD36	948	1	0.49	Z32765_at	0.49	NA	NA	NA	NA	NA	NA	NA	NA								
242	MFAP4	4239	1	0.48	NA	NA	39066_at	0.48	NA	NA	NA	NA	NA	NA								
243	F8	2157	1	0.48	M14113_at	0.48	NA	NA	NA	NA	NA	NA	NA	NA								
244	GPD1L	23171	1	0.48	NA	NA	38394_at	0.48	NA	NA	NA	NA	NA	NA								
245	HOXA5	3202	1	0.48	M26679_at	0.48	NA	NA	NA	NA	NA	NA	NA	NA								
246	CDW52	1043	1	0.47	X62466_at	0.47	NA	NA	NA	NA	NA	NA	NA	NA								
247	MS4A1	931	1	0.47	M89796_rna1_at	0.47	NA	NA	NA	NA	NA	NA	NA	NA								
248	FEZ1	9638	1	0.47	U60060_at	0.47	NA	NA	NA	NA	NA	NA	NA	NA								
249	GPRK5	2869	1	0.47	L15388_at	0.47	NA	NA	NA	NA	NA	NA	NA	NA								
250	GPC3	2719	1	0.47	Z37987_s_at	0.47	NA	NA	NA	NA	NA	NA	NA	NA								
251	MT1G	4495	1	0.47	K01383_at	0.47	NA	NA	NA	NA	NA	NA	NA	NA								
252	ADRB2	154	1	0.47	NA	NA	610_at	0.47	NA	NA	NA	NA	NA	NA								
253	SATB1	6304	1	0.47	M97287_at	0.47	NA	NA	NA	NA	NA	NA	NA	NA								
254	GNG11	2791	1	0.47	U31384_at	0.47	NA	NA	NA	NA	NA	NA	NA	NA								
255	CD97	976	1	0.47	U76764_s_at	0.47	NA	NA	NA	NA	NA	NA	NA	NA								

No.	Gene Symbol	NCBI GeneID	No. Of datasets	Median CC	Beer ProbeID	Beer CC	Bhattacharjee ProbeID	Bhattacharjee CC	Garber ProbeID	Garber CC	Kim ProbeID	Kim CC	Kuner ProbeID	Kuner CC	Cancer	Respiratory Disease	Cell Cycle	Lipid Metabolism	Molecular Transport	Small Molecule Biochemistry	Cellular Movement	Cell-to-Cell Signaling
256	AADAC	13	1	0.47	L32179_at	0.47	NA	NA	NA	NA	NA	NA	NA	NA								
257	FBLN5	10516	1	0.47	NA	NA	39038_at	0.47	NA	NA	NA	NA	NA	NA								
258	LBC	3928	1	0.46	HG2167-HT2237_at	0.46	NA	NA	NA	NA	NA	NA	NA	NA								
259	ADARB1	104	1	0.46	U76421_at	0.46	NA	NA	NA	NA	NA	NA	NA	NA								
260	MNDA	4332	1	0.46	M81750_at	0.46	NA	NA	NA	NA	NA	NA	NA	NA								
261	ATP8A1	10396	1	0.46	NA	NA	33267_at	0.46	NA	NA	NA	NA	NA	NA								
262	HYAL2	8692	1	0.46	NA	NA	37042_at	0.46	NA	NA	NA	NA	NA	NA								
263	METTL7A	25840	1	0.46	NA	NA	38717_at	0.46	NA	NA	NA	NA	NA	NA								
264	FRMD4B	23150	1	0.46	NA	NA	38336_at	0.46	NA	NA	NA	NA	NA	NA								
265	BCHE	590	1	0.46	M16474_s_at	0.46	NA	NA	NA	NA	NA	NA	NA	NA								
266	ABCA8	10351	1	0.46	NA	NA	35717_at	0.46	NA	NA	NA	NA	NA	NA								
267	ABCB1	5243	1	0.46	M14758_at	0.46	NA	NA	NA	NA	NA	NA	NA	NA								
268	GYPC	2995	1	0.45	M36284_s_at	0.45	NA	NA	NA	NA	NA	NA	NA	NA								
269	SCYA14	6358	1	0.45	Z49269_at	0.45	NA	NA	NA	NA	NA	NA	NA	NA								
270	PPAP2B	8613	1	0.45	U79294_at	0.45	NA	NA	NA	NA	NA	NA	NA	NA								
271	THBD	7056	1	0.45	J02973_rna1_at	0.45	NA	NA	NA	NA	NA	NA	NA	NA								
272	TLE4	7091	1	0.45	M99439_at	0.45	NA	NA	NA	NA	NA	NA	NA	NA								
273	LMNB1	4001	1	-0.31	NA	NA	37985_at	-0.31	NA	NA	NA	NA	NA	NA								
274	FBL	2091	1	-0.31	NA	NA	39173_at	-0.31	NA	NA	NA	NA	NA	NA								
275	SNRPD2	6633	1	-0.31	NA	NA	35270_at	-0.31	NA	NA	NA	NA	NA	NA								
276	LSM2	57819	1	-0.32	NA	NA	41375_at	-0.32	NA	NA	NA	NA	NA	NA								
277	LEPREL2	10536	1	-0.32	NA	NA	39973_at	-0.32	NA	NA	NA	NA	NA	NA								
278	MCM5	4174	1	-0.32	NA	NA	982_at	-0.32	NA	NA	NA	NA	NA	NA								
279	COL5A2	1290	1	-0.32	NA	NA	38420_at	-0.32	NA	NA	NA	NA	NA	NA								
280	AURKA	6790	1	-0.32	NA	NA	34851_at	-0.32	NA	NA	NA	NA	NA	NA								
281	AK3L1	205	1	-0.32	NA	NA	32331_at	-0.32	NA	NA	NA	NA	NA	NA								
282	UNG	7374	1	-0.32	NA	NA	37686_s_at	-0.32	NA	NA	NA	NA	NA	NA								
283	CAD	790	1	-0.32	NA	NA	32031_at	-0.32	NA	NA	NA	NA	NA	NA								
284	CENPA	1058	1	-0.32	NA	NA	527_at	-0.32	NA	NA	NA	NA	NA	NA								
285	CCNB2	9133	1	-0.32	NA	NA	32263_at	-0.32	NA	NA	NA	NA	NA	NA								
286	KIAA0159	9918	1	-0.32	D63880_at	-0.32	NA	NA	NA	NA	NA	NA	NA	NA								
287	HSPCA	3320	1	-0.32	X15183_at	-0.32	NA	NA	NA	NA	NA	NA	NA	NA								
288	TIMELESS	8914	1	-0.32	NA	NA	41626_at	-0.32	NA	NA	NA	NA	NA	NA								
289	CCNB1	891	1	-0.32	NA	NA	34736_at	-0.32	NA	NA	NA	NA	NA	NA								
290	COL6A3	1293	1	-0.32	X52022_at	-0.32	NA	NA	NA	NA	NA	NA	NA	NA								
291	CIB2	10518	1	-0.32	NA	NA	39903_at	-0.32	NA	NA	NA	NA	NA	NA								
292	FUBP1	8880	1	-0.32	U05040_at	-0.32	NA	NA	NA	NA	NA	NA	NA	NA								
293	AK3	50808	1	-0.32	X60673_rna1_at	-0.32	NA	NA	NA	NA	NA	NA	NA	NA								
294	IRAK1	3654	1	-0.32	L76191_at	-0.32	NA	NA	NA	NA	NA	NA	NA	NA								
295	MTHFD2	10797	1	-0.33	X16396_at	-0.33	NA	NA	NA	NA	NA	NA	NA	NA								
296	RPL39L	116832	1	-0.33	NA	NA	35549_at	-0.33	NA	NA	NA	NA	NA	NA								
297	TYMS	7298	1	-0.33	NA	NA	37899_at	-0.33	NA	NA	NA	NA	NA	NA								
298	ORC1L	4998	1	-0.33	U40152_s_at	-0.33	NA	NA	NA	NA	NA	NA	NA	NA								
299	NRF1	4899	1	-0.33	L22454_at	-0.33	NA	NA	NA	NA	NA	NA	NA	NA								
300	YARS	8565	1	-0.33	U40714_at	-0.33	NA	NA	NA	NA	NA	NA	NA	NA								
301	ITGAV	3685	1	-0.33	M14648_at	-0.33	NA	NA	NA	NA	NA	NA	NA	NA								
302	ITGB5	3693	1	-0.33	J05633_at	-0.33	NA	NA	NA	NA	NA	NA	NA	NA								
303	TUBB5	203068	1	-0.33	HG4322-HT4592_at	-0.33	NA	NA	NA	NA	NA	NA	NA	NA								
304	UBE2I	7329	1	-0.33	U45328_s_at	-0.33	NA	NA	NA	NA	NA	NA	NA	NA								
305	SKIV2L	6499	1	-0.33	U09877_at	-0.33	NA	NA	NA	NA	NA	NA	NA	NA								
306	MMP1	4312	1	-0.33	NA	NA	38428_at	-0.33	NA	NA	NA	NA	NA	NA								
307	AHCY	191	1	-0.33	NA	NA	40821_at	-0.33	NA	NA	NA	NA	NA	NA								
308	CBX1	10951	1	-0.33	U35451_at	-0.33	NA	NA	NA	NA	NA	NA	NA	NA								
309	MAZ	4150	1	-0.33	NA	NA	32553_at	-0.33	NA	NA	NA	NA	NA	NA								
310	KPNB1	3837	1	-0.33	L38951_at	-0.33	NA	NA	NA	NA	NA	NA	NA	NA								
311	TWIST1	7291	1	-0.33	NA	NA	40328_at	-0.33	NA	NA	NA	NA	NA	NA								
312	D13S106E	10208	1	-0.33	X59131_at	-0.33	NA	NA	NA	NA	NA	NA	NA	NA								
313	PPIA	5478	1	-0.33	X52851_rna1_at	-0.33	NA	NA	NA	NA	NA	NA	NA	NA								
314	HSPD1	3329	1	-0.33	M22382_at	-0.33	NA	NA	NA	NA	NA	NA	NA	NA								
315	MMP15	4324	1	-0.33	D86331_s_at	-0.33	NA	NA	NA	NA	NA	NA	NA	NA								
316	PAICS	10606	1	-0.33	X53793_at	-0.33	NA	NA	NA	NA	NA	NA	NA	NA								
317	CTPS	1503	1	-0.33	NA	NA	32441_at	-0.33	NA	NA	NA	NA	NA	NA								
318	FADD	8772	1	-0.33	X84709_at	-0.33	NA	NA	NA	NA	NA	NA	NA	NA								
319	ACTN1	87	1	-0.33	M95178_at	-0.33	NA	NA	NA	NA	NA	NA	NA	NA								
320	TRAP1	10131	1	-0.33	NA	NA	1468_at	-0.33	NA	NA	NA	NA	NA	NA								
321	EPRS	2058	1	-0.34	X54326_at	-0.34	NA	NA	NA	NA	NA	NA	NA	NA								
322	MCM7	4176	1	-0.34	NA	NA	947_at	-0.34	NA	NA	NA	NA	NA	NA								
323	EYA1	2138	1	-0.34	Y10260_at	-0.34	NA	NA	NA	NA	NA	NA	NA	NA								
324	H2AFX	3014	1	-0.34	X14850_at	-0.34	NA	NA	NA	NA	NA	NA	NA	NA								
325	GINS1	9837	1	-0.34	NA	NA	39677_at	-0.34	NA	NA	NA	NA	NA	NA								
326	SOX4	6659	1	-0.34	NA	NA	33131_at	-0.34	NA	NA	NA	NA	NA	NA								
327	ELAVL1	1994	1	-0.34	U38175_at	-0.34	NA	NA	NA	NA	NA	NA	NA	NA								
328	MYL6B	140465	1	-0.34	NA	NA	38251_at	-0.34	NA	NA	NA	NA	NA	NA								
329	ACTG1	71	1	-0.34	M19283_at	-0.34	NA	NA	NA	NA	NA	NA	NA	NA								
330	YWHAQ	10971	1	-0.34	X56468_at	-0.34	NA	NA	NA	NA	NA	NA	NA	NA								
331	DYRK2	8445	1	-0.34	Y09216_at	-0.34	NA	NA	NA	NA	NA	NA	NA	NA								
332	KPNA2	3838	1	-0.34	U28386_at	-0.34	NA	NA	NA	NA	NA	NA	NA	NA								
333	NDC80	10403	1	-0.34	NA	NA	40041_at	-0.34	NA	NA	NA	NA	NA	NA								
334	IL13RA1	3597	1	-0.34	Y10659_at	-0.34	NA	NA	NA	NA	NA	NA	NA	NA								
335	CPZ	8532	1	-0.34	U83411_at	-0.34	NA	NA	NA	NA	NA	NA	NA	NA								
336	TACSTD1	4072	1	-0.34	M93036_at	-0.34	NA	NA	NA	NA	NA	NA	NA	NA								
337	D6S51E	7916	1	-0.34	M33518_at	-0.34	NA	NA	NA	NA	NA	NA	NA	NA								
338	PMAIP1	5366	1	-0.34	D90070_s_at	-0.34	NA	NA	NA	NA	NA	NA	NA	NA								
339	SNRPE	6635	1	-0.34	M21259_at	-0.34	NA	NA	NA	NA	NA	NA	NA	NA								
340	NDUFS6	4726	1	-0.34	NA	NA	34865_at	-0.34	NA	NA	NA	NA	NA	NA								

No.	Gene Symbol	NCBI GeneID	No. Of datasets	Median CC	Beer ProbeID	Beer CC	Bhattacharjee ProbeID	Bhattacharjee CC	Garber ProbeID	Garber CC	Kim ProbeID	Kim CC	Kuner ProbeID	Kuner CC	Cancer	Respiratory Disease	Cell Cycle	Lipid Metabolism	Molecular Transport	Small Molecule Biochemistry	Cellular Movement	Cell-to-Cell Signaling
341	RASA2	5922	1	-0.34	D78156_at	-0.34	NA	NA	NA	NA	NA	NA	NA	NA								
342	JTV1	7965	1	-0.34	U24169_at	-0.34	NA	NA	NA	NA	NA	NA	NA	NA								
343	IMMT	10989	1	-0.34	L42572_at	-0.34	NA	NA	NA	NA	NA	NA	NA	NA								
344	AURKB	9212	1	-0.34	NA	NA	33266_at	-0.34	NA	NA	NA	NA	NA	NA								
345	PTK2	5747	1	-0.34	HG3075-HT3236_s_at	-0.34	NA	NA	NA	NA	NA	NA	NA	NA								
346	WDR43	23160	1	-0.35	NA	NA	38250_at	-0.35	NA	NA	NA	NA	NA	NA								
347	ARD1A	8260	1	-0.35	NA	NA	31873_at	-0.35	NA	NA	NA	NA	NA	NA								
348	TRA1	7184	1	-0.35	X15187_at	-0.35	NA	NA	NA	NA	NA	NA	NA	NA								
349	ADPRT	142	1	-0.35	J03473_at	-0.35	NA	NA	NA	NA	NA	NA	NA	NA								
350	PABPC1	26986	1	-0.35	Z48501_s_at	-0.35	NA	NA	NA	NA	NA	NA	NA	NA								
351	GFPT1	2673	1	-0.35	M90516_at	-0.35	NA	NA	NA	NA	NA	NA	NA	NA								
352	MEF2D	4209	1	-0.35	HG4668-HT5083_s_at	-0.35	NA	NA	NA	NA	NA	NA	NA	NA								
353	CCT3	7203	1	-0.35	X74801_at	-0.35	NA	NA	NA	NA	NA	NA	NA	NA								
354	CDC25C	995	1	-0.35	M34065_at	-0.35	NA	NA	NA	NA	NA	NA	NA	NA								
355	RNASEH2A	10535	1	-0.35	NA	NA	35141_at	-0.35	NA	NA	NA	NA	NA	NA								
356	PCTK1	5127	1	-0.35	NA	NA	39183_at	-0.35	NA	NA	NA	NA	NA	NA								
357	MDK	4192	1	-0.35	M94250_at	-0.35	NA	NA	NA	NA	NA	NA	NA	NA								
358	MYO1C	4641	1	-0.35	U14391_at	-0.35	NA	NA	NA	NA	NA	NA	NA	NA								
359	MMP9	4318	1	-0.35	NA	NA	31859_at	-0.35	NA	NA	NA	NA	NA	NA								
360	GLG1	2734	1	-0.35	U28811_at	-0.35	NA	NA	NA	NA	NA	NA	NA	NA								
361	TSSC3	7262	1	-0.35	AF001294_at	-0.35	NA	NA	NA	NA	NA	NA	NA	NA								
362	HMGA1	3159	1	-0.35	NA	NA	39704_s_at	-0.35	NA	NA	NA	NA	NA	NA								
363	PHB	5245	1	-0.36	S85655_at	-0.36	NA	NA	NA	NA	NA	NA	NA	NA								
364	PKM2	5315	1	-0.36	X56494_at	-0.36	NA	NA	NA	NA	NA	NA	NA	NA								
365	DDX8	1659	1	-0.36	D50487_at	-0.36	NA	NA	NA	NA	NA	NA	NA	NA								
366	CDC20	991	1	-0.36	NA	NA	38414_at	-0.36	NA	NA	NA	NA	NA	NA								
367	SNRPA	6626	1	-0.36	NA	NA	40842_at	-0.36	NA	NA	NA	NA	NA	NA								
368	KPNA1	3836	1	-0.36	S75295_at	-0.36	NA	NA	NA	NA	NA	NA	NA	NA								
369	CDT1	81620	1	-0.36	NA	NA	34510_at	-0.36	NA	NA	NA	NA	NA	NA								
370	BGN	633	1	-0.36	J04599_at	-0.36	NA	NA	NA	NA	NA	NA	NA	NA								
371	SLC20A1	6574	1	-0.36	L20859_at	-0.36	NA	NA	NA	NA	NA	NA	NA	NA								
372	UBE2M	9040	1	-0.36	NA	NA	33782_r_at	-0.36	NA	NA	NA	NA	NA	NA								
373	MIF	4282	1	-0.36	NA	NA	895_at	-0.36	NA	NA	NA	NA	NA	NA								
374	NMT1	4836	1	-0.36	M86707_at	-0.36	NA	NA	NA	NA	NA	NA	NA	NA								
375	PSMD1	5707	1	-0.36	D44466_at	-0.36	NA	NA	NA	NA	NA	NA	NA	NA								
376	NOL5A	10528	1	-0.37	NA	NA	34882_at	-0.37	NA	NA	NA	NA	NA	NA								
377	KRT10	3858	1	-0.37	J04029_s_at	-0.37	NA	NA	NA	NA	NA	NA	NA	NA								
378	MARCKSL1	65108	1	-0.37	NA	NA	36174_at	-0.37	NA	NA	NA	NA	NA	NA								
379	CSPG2	1462	1	-0.37	U16306_at	-0.37	NA	NA	NA	NA	NA	NA	NA	NA								
380	ENO1	2023	1	-0.37	M14328_s_at	-0.37	NA	NA	NA	NA	NA	NA	NA	NA								
381	CARM1	10498	1	-0.37	NA	NA	40182_s_at	-0.37	NA	NA	NA	NA	NA	NA								
382	GTSE1	51512	1	-0.37	NA	NA	39872_at	-0.37	NA	NA	NA	NA	NA	NA								
383	UBE2S	27338	1	-0.37	NA	NA	894_g_at	-0.37	NA	NA	NA	NA	NA	NA								
384	TCOF1	6949	1	-0.37	NA	NA	40597_g_at	-0.37	NA	NA	NA	NA	NA	NA								
385	COL1A1	1277	1	-0.37	Z74615_at	-0.37	NA	NA	NA	NA	NA	NA	NA	NA								
386	TIAL1	7073	1	-0.37	M96954_s_at	-0.37	NA	NA	NA	NA	NA	NA	NA	NA								
387	SLC6A8	6535	1	-0.38	NA	NA	40926_at	-0.38	NA	NA	NA	NA	NA	NA								
388	SPAG5	10615	1	-0.38	NA	NA	32120_at	-0.38	NA	NA	NA	NA	NA	NA								
389	CNP	1267	1	-0.38	M19650_s_at	-0.38	NA	NA	NA	NA	NA	NA	NA	NA								
390	ACLY	47	1	-0.38	X64330_at	-0.38	NA	NA	NA	NA	NA	NA	NA	NA								
391	ESPL1	9700	1	-0.38	NA	NA	38158_at	-0.38	NA	NA	NA	NA	NA	NA								
392	FAP	2191	1	-0.38	U09278_at	-0.38	NA	NA	NA	NA	NA	NA	NA	NA								
393	TOMM40	10452	1	-0.38	NA	NA	35620_at	-0.38	NA	NA	NA	NA	NA	NA								
394	OSF-2	10631	1	-0.38	D13666_s_at	-0.38	NA	NA	NA	NA	NA	NA	NA	NA								
395	USP14	9097	1	-0.38	U30888_at	-0.38	NA	NA	NA	NA	NA	NA	NA	NA								
396	DDXL	10212	1	-0.38	U90426_at	-0.38	NA	NA	NA	NA	NA	NA	NA	NA								
397	ATP2A2	488	1	-0.39	M23114_at	-0.39	NA	NA	NA	NA	NA	NA	NA	NA								
398	SLC5A5	6528	1	-0.39	U66088_at	-0.39	NA	NA	NA	NA	NA	NA	NA	NA								
399	COL1A2	1278	1	-0.39	Z74616_s_at	-0.39	NA	NA	NA	NA	NA	NA	NA	NA								
400	P4HB	5034	1	-0.39	X05130_s_at	-0.39	NA	NA	NA	NA	NA	NA	NA	NA								
401	NIPSNAP1	8508	1	-0.39	NA	NA	40866_at	-0.39	NA	NA	NA	NA	NA	NA								
402	CSNK1D	1453	1	-0.40	U29171_at	-0.40	NA	NA	NA	NA	NA	NA	NA	NA								
403	UGTREL1	10237	1	-0.40	D87989_at	-0.40	NA	NA	NA	NA	NA	NA	NA	NA								
404	SRPK1	6732	1	-0.40	U09564_at	-0.40	NA	NA	NA	NA	NA	NA	NA	NA								
405	ILF3	3609	1	-0.40	U10324_at	-0.40	NA	NA	NA	NA	NA	NA	NA	NA								
406	KIF2C	11004	1	-0.40	NA	NA	36837_at	-0.40	NA	NA	NA	NA	NA	NA								
407	BOLA2	552900	1	-0.41	NA	NA	41732_at	-0.41	NA	NA	NA	NA	NA	NA								
408	EIF4EBP1	1978	1	-0.41	NA	NA	31597_r_at	-0.41	NA	NA	NA	NA	NA	NA								
409	APBA2	321	1	-0.41	U79255_at	-0.41	NA	NA	NA	NA	NA	NA	NA	NA								
410	DTYMK	1841	1	-0.41	L16991_at	-0.41	NA	NA	NA	NA	NA	NA	NA	NA								
411	LDHA	3939	1	-0.42	X02152_at	-0.42	NA	NA	NA	NA	NA	NA	NA	NA								
412	NDUFA8	4702	1	-0.43	NA	NA	NA	NA	NA	NA	218160_at	-0.43	NA	NA								
413	TPX2	22974	1	-0.43	NA	NA	39109_at	-0.43	NA	NA	NA	NA	NA	NA								
414	LOC344887	344887	1	-0.43	NA	NA	NA	NA	NA	NA	241418_at	-0.43	NA	NA								
415	ATG4D	84971	1	-0.43	NA	NA	NA	NA	NA	NA	226871_s_at	-0.43	NA	NA								
416	PITPNB	23760	1	-0.43	NA	NA	NA	NA	NA	NA	202522_at	-0.43	NA	NA								
417	C6orf117	112609	1	-0.43	NA	NA	NA	NA	NA	NA	227226_at	-0.43	NA	NA								
418	ZNF367	195828	1	-0.43	NA	NA	NA	NA	NA	NA	229551_x_at	-0.43	NA	NA								
419	PMS2L11	5389	1	-0.43	NA	NA	NA	NA	NA	NA	210707_x_at	-0.43	NA	NA								
420	STRBP	55342	1	-0.43	NA	NA	NA	NA	NA	NA	223246_s_at	-0.43	NA	NA								
421	POLQ	10721	1	-0.43	NA	NA	NA	NA	NA	NA	219510_at	-0.43	NA	NA								
422	KIAA0888	26049	1	-0.43	NA	NA	NA	NA	NA	NA	235048_at	-0.43	NA	NA								
423	YEATS2	55689	1	-0.44	NA	NA	NA	NA	NA	NA	221203_s_at	-0.44	NA	NA								
424	GPR87	53836	1	-0.44	NA	NA	NA	NA	NA	NA	219936_s_at	-0.44	NA	NA								
425	SLC1A4	6509	1	-0.44	NA	NA	NA	NA	NA	NA	212811_x_at	-0.44	NA	NA								

No.	Gene Symbol	NCBI GeneID	No. Of datasets	Median CC	Beer ProbeID	Beer CC	Bhattacharjee ProbeID	Bhattacharjee CC	Garber ProbeID	Garber CC	Kim ProbeID	Kim CC	Kuner ProbeID	Kuner CC	Cancer	Respiratory Disease	Cell Cycle	Lipid Metabolism	Molecular Transport	Small Molecule Biochemistry	Cellular Movement	Cell-to-Cell Signaling
426	ABCC1	4363	1	-0.44	NA	NA	NA	NA	NA	NA	202805_s_at	-0.44	NA	NA								
427	ARNTL2	56938	1	-0.44	NA	NA	NA	NA	NA	NA	223586_at	-0.44	NA	NA								
428	SMCR7L	54471	1	-0.44	NA	NA	NA	NA	NA	NA	224319_s_at	-0.44	NA	NA								
429	TMEM117	84216	1	-0.44	NA	NA	NA	NA	NA	NA	223594_at	-0.44	NA	NA								
430	MICALL1	85377	1	-0.44	NA	NA	NA	NA	NA	NA	221779_at	-0.44	NA	NA								
431	CDK5R1	8851	1	-0.44	NA	NA	NA	NA	NA	NA	204995_at	-0.44	NA	NA								
432	HR	3264	1	-0.44	NA	NA	NA	NA	NA	NA	241355_at	-0.44	NA	NA								
433	GRHL3	57822	1	-0.44	NA	NA	NA	NA	NA	NA	232116_at	-0.44	NA	NA								
434	CRSP8	9442	1	-0.44	NA	NA	NA	NA	NA	NA	51176_at	-0.44	NA	NA								
435	TH1L	51497	1	-0.44	NA	NA	NA	NA	NA	NA	220607_x_at	-0.44	NA	NA								
436	FAM20B	9917	1	-0.44	NA	NA	NA	NA	NA	NA	202916_s_at	-0.44	NA	NA								
437	MGC61598	441478	1	-0.44	NA	NA	NA	NA	NA	NA	226499_at	-0.44	NA	NA								
438	WDR72	256764	1	-0.44	NA	NA	NA	NA	NA	NA	236741_at	-0.44	NA	NA								
439	CAMSAP1	157922	1	-0.44	NA	NA	NA	NA	NA	NA	212711_at	-0.44	NA	NA								
440	FXR1	8087	1	-0.44	NA	NA	NA	NA	NA	NA	201636_at	-0.44	NA	NA								
441	UPF3B	65109	1	-0.44	NA	NA	NA	NA	NA	NA	218757_s_at	-0.44	NA	NA								
442	GINS3	64785	1	-0.44	NA	NA	NA	NA	NA	NA	45633_at	-0.44	NA	NA								
443	C21orf91	54149	1	-0.44	NA	NA	NA	NA	NA	NA	220941_s_at	-0.44	NA	NA								
444	CELSR2	1952	1	-0.44	NA	NA	NA	NA	NA	NA	204029_at	-0.44	NA	NA								
445	FZD7	8324	1	-0.44	NA	NA	NA	NA	NA	NA	203705_s_at	-0.44	NA	NA								
446	SFRS2	6427	1	-0.45	NA	NA	NA	NA	NA	NA	200754_x_at	-0.45	NA	NA								
447	PTGFRN	5738	1	-0.45	NA	NA	NA	NA	NA	NA	224950_at	-0.45	NA	NA								
448	NAT5	51126	1	-0.45	NA	NA	NA	NA	NA	NA	223040_at	-0.45	NA	NA								
449	IPPK	64768	1	-0.45	NA	NA	NA	NA	NA	NA	219092_s_at	-0.45	NA	NA								
450	ZNF639	51193	1	-0.45	NA	NA	NA	NA	NA	NA	222623_s_at	-0.45	NA	NA								
451	DSC3	1825	1	-0.45	NA	NA	NA	NA	NA	NA	206033_s_at	-0.45	NA	NA								
452	LSG1	55341	1	-0.45	NA	NA	NA	NA	NA	NA	221535_at	-0.45	NA	NA								
453	MRPS22	56945	1	-0.45	NA	NA	NA	NA	NA	NA	228059_x_at	-0.45	NA	NA								
454	TP73L	8626	1	-0.45	NA	NA	NA	NA	NA	NA	207382_at	-0.45	NA	NA								
455	PLD1	5337	1	-0.45	NA	NA	NA	NA	NA	NA	177_at	-0.45	NA	NA								
456	VSNL1	7447	1	-0.45	NA	NA	NA	NA	NA	NA	203797_at	-0.45	NA	NA								
457	ADAM23	8745	1	-0.45	NA	NA	NA	NA	NA	NA	206046_at	-0.45	NA	NA								
458	PKP1	5317	1	-0.45	NA	NA	NA	NA	NA	NA	205724_at	-0.45	NA	NA								
459	CYP4F3	4051	1	-0.45	NA	NA	NA	NA	NA	NA	206515_at	-0.45	NA	NA								
460	TBCCD1	55171	1	-0.45	NA	NA	NA	NA	NA	NA	206451_at	-0.45	NA	NA								
461	TEX10	54881	1	-0.45	NA	NA	NA	NA	NA	NA	218104_at	-0.45	NA	NA								
462	TRIM59	286827	1	-0.46	NA	NA	NA	NA	NA	NA	227801_at	-0.46	NA	NA								
463	SRXN1	140809	1	-0.46	NA	NA	NA	NA	NA	NA	225252_at	-0.46	NA	NA								
464	BTBD11	121551	1	-0.46	NA	NA	NA	NA	NA	NA	238692_at	-0.46	NA	NA								
465	TRIM29	23650	1	-0.46	NA	NA	NA	NA	NA	NA	211002_s_at	-0.46	NA	NA								
466	SLC9A3R1	9368	1	-0.46	NA	NA	NA	NA	NA	NA	201349_at	-0.46	NA	NA								
467	DIP2B	57609	1	-0.46	NA	NA	NA	NA	NA	NA	224872_at	-0.46	NA	NA								
468	PUS7	54517	1	-0.46	NA	NA	NA	NA	AA434411	-0.46	NA	NA	NA	NA								
469	MRPL47	57129	1	-0.46	NA	NA	NA	NA	NA	NA	223480_s_at	-0.46	NA	NA								
470	PSMD11	5717	1	-0.46	NA	NA	NA	NA	NA	NA	208777_s_at	-0.46	NA	NA								
471	PRPF4	9128	1	-0.46	NA	NA	NA	NA	NA	NA	209161_at	-0.46	NA	NA								
472	TRAF7	84231	1	-0.46	NA	NA	NA	NA	AA410468	-0.46	NA	NA	NA	NA								
473	PPP2R2C	5522	1	-0.46	NA	NA	NA	NA	N40919	-0.46	NA	NA	NA	NA								
474	DUSP14	11072	1	-0.46	NA	NA	NA	NA	NA	NA	203367_at	-0.46	NA	NA								
475	FTSJ1	24140	1	-0.46	NA	NA	NA	NA	NA	NA	NA	NA	213937_s_at	-0.46								
476	MGC17337	91283	1	-0.46	NA	NA	NA	NA	NA	NA	NA	NA	1552277_a_at	-0.46								
477	GREM1	26585	1	-0.46	NA	NA	NA	NA	NA	NA	NA	NA	218469_at	-0.46								
478	HSPA1B	3304	1	-0.46	NA	NA	NA	NA	NA	NA	NA	NA	202581_at	-0.46								
479	C3orf37	56941	1	-0.46	NA	NA	NA	NA	NA	NA	201678_s_at	-0.46	NA	NA								
480	LOC440160	440160	1	-0.47	NA	NA	NA	NA	NA	NA	NA	NA	242881_x_at	-0.47								
481	ZNF286	57335	1	-0.47	NA	NA	NA	NA	AA464019	-0.47	NA	NA	NA	NA								
482	ZBTB33	10009	1	-0.47	NA	NA	NA	NA	NA	NA	226255_at	-0.47	NA	NA								
483	HOXA1	3198	1	-0.47	NA	NA	NA	NA	NA	NA	NA	NA	214639_s_at	-0.47								
484	FEN1	2237	1	-0.47	NA	NA	NA	NA	AA620553	-0.47	NA	NA	NA	NA								
485	C1orf131	128061	1	-0.47	NA	NA	NA	NA	NA	NA	226242_at	-0.47	NA	NA								
486	DKC1	1736	1	-0.47	NA	NA	NA	NA	NA	NA	NA	NA	216212_s_at	-0.47								
487	DOCK7	85440	1	-0.47	NA	NA	NA	NA	NA	NA	NA	NA	225384_at	-0.47								
488	CDC6	990	1	-0.47	NA	NA	NA	NA	H59203	-0.47	NA	NA	NA	NA								
489	NDRG4	65009	1	-0.47	NA	NA	NA	NA	NA	NA	209159_s_at	-0.47	NA	NA								
490	FLJ32549	144577	1	-0.47	NA	NA	NA	NA	NA	NA	NA	NA	235026_at	-0.47								
491	DUT	1854	1	-0.47	NA	NA	NA	NA	AA433910	-0.47	NA	NA	NA	NA								
492	LOXL2	4017	1	-0.47	NA	NA	NA	NA	NA	NA	NA	NA	202998_s_at	-0.47								
493	E2F1	1869	1	-0.47	NA	NA	NA	NA	H61303	-0.47	NA	NA	NA	NA								
494	SELI	85465	1	-0.47	NA	NA	NA	NA	NA	NA	224888_at	-0.47	NA	NA								
495	SERPINB7	8710	1	-0.47	NA	NA	NA	NA	NA	NA	NA	NA	206421_s_at	-0.47								
496	MCM6	4175	1	-0.47	NA	NA	NA	NA	AA663995	-0.47	NA	NA	NA	NA								
497	KDELC1	79070	1	-0.47	NA	NA	NA	NA	NA	NA	NA	NA	219479_at	-0.47								
498	HOXB3	3213	1	-0.47	NA	NA	NA	NA	NA	NA	NA	NA	228904_at	-0.47								
499	HUS1	3364	1	-0.47	NA	NA	NA	NA	NA	NA	NA	NA	217618_x_at	-0.47								
500	FBXO45	200933	1	-0.47	NA	NA	NA	NA	NA	NA	NA	NA	225100_at	-0.47								
501	BUB1	699	1	-0.48	NA	NA	NA	NA	AA430092	-0.48	NA	NA	NA	NA								
502	BRCA1	672	1	-0.48	NA	NA	NA	NA	H90415	-0.48	NA	NA	NA	NA								
503	E2F7	144455	1	-0.48	NA	NA	NA	NA	NA	NA	228033_at	-0.48	NA	NA								
504	KRT6B	3854	1	-0.48	NA	NA	NA	NA	AA026418	-0.48	NA	NA	NA	NA								
505	CENPJ	55835	1	-0.48	NA	NA	NA	NA	NA	NA	NA	NA	223513_at	-0.48								
506	DDIT3	1649	1	-0.48	NA	NA	NA	NA	NA	NA	NA	NA	209383_at	-0.48								
507	FAM72A	389835	1	-0.48	NA	NA	NA	NA	R22949	-0.48	NA	NA	NA	NA								
508	GANAB	2594	1	-0.48	NA	NA	NA	NA	NA	NA	NA	NA	214626_s_at	-0.48								
509	MGC13102	84283	1	-0.48	NA	NA	NA	NA	NA	NA	NA	NA	223544_at	-0.48								
510	WDR45L	56270	1	-0.48	NA	NA	NA	NA	NA	NA	NA	NA	209076_s_at	-0.48								

No.	Gene Symbol	NCBI GeneID	No. Of datasets	Median CC	Beer ProbeID	Beer CC	Bhattacharjee ProbeID	Bhattacharjee CC	Garber ProbeID	Garber CC	Kim ProbeID	Kim CC	Kuner ProbeID	Kuner CC	Cancer	Respiratory Disease	Cell Cycle	Lipid Metabolism	Molecular Transport	Small Molecule Biochemistry	Cellular Movement	Cell-to-Cell Signaling
511	HOMER3	9454	1	-0.48	NA	NA	NA	NA	NA	NA	NA	NA	204647_at	-0.48								
512	AKR1B10	57016	1	-0.48	NA	NA	NA	NA	NA	NA	206561_s_at	-0.48	NA	NA								
513	WDR5	11091	1	-0.48	NA	NA	NA	NA	AA421266	-0.48	NA	NA	NA	NA								
514	HSPA4L	22824	1	-0.48	NA	NA	NA	NA	NA	NA	205543_at	-0.48	NA	NA								
515	ANTXR1	84168	1	-0.48	NA	NA	NA	NA	NA	NA	NA	NA	220092_s_at	-0.48								
516	FANCG	2189	1	-0.48	NA	NA	NA	NA	AA427484	-0.48	NA	NA	NA	NA								
517	TRA16	126382	1	-0.48	NA	NA	NA	NA	NA	NA	NA	NA	226839_at	-0.48								
518	SIAH2	6478	1	-0.48	NA	NA	NA	NA	AA029041	-0.48	NA	NA	NA	NA								
519	INHBA	3624	1	-0.48	NA	NA	NA	NA	NA	NA	NA	NA	210511_s_at	-0.48								
520	LOC391426	391426	1	-0.48	NA	NA	NA	NA	AA431571	-0.48	NA	NA	NA	NA								
521	TCP11L1	55346	1	-0.48	NA	NA	NA	NA	R35500	-0.48	NA	NA	NA	NA								
522	GMPS	8833	1	-0.48	NA	NA	NA	NA	NA	NA	214431_at	-0.48	NA	NA								
523	BXDC2	55299	1	-0.48	NA	NA	NA	NA	AA454607	-0.48	NA	NA	NA	NA								
524	EYA2	2139	1	-0.49	NA	NA	NA	NA	NA	NA	NA	NA	209692_at	-0.49								
525	FAM11A	84548	1	-0.49	NA	NA	NA	NA	NA	NA	NA	NA	227880_s_at	-0.49								
526	RIS1	25907	1	-0.49	NA	NA	NA	NA	NA	NA	NA	NA	213338_at	-0.49								
527	ATAD2	29028	1	-0.49	NA	NA	NA	NA	H58234	-0.49	NA	NA	NA	NA								
528	FBXO32	114907	1	-0.49	NA	NA	NA	NA	NA	NA	NA	NA	225328_at	-0.49								
529	RBM30	83759	1	-0.49	NA	NA	NA	NA	NA	NA	NA	NA	209497_s_at	-0.49								
530	PSF1	5665	1	-0.49	NA	NA	NA	NA	NA	NA	NA	NA	206102_at	-0.49								
531	HS3ST3A1	9955	1	-0.49	NA	NA	NA	NA	NA	NA	NA	NA	219985_at	-0.49								
532	GMNN	51053	1	-0.49	NA	NA	NA	NA	AA447662	-0.49	NA	NA	NA	NA								
533	MLLT11	10962	1	-0.49	NA	NA	NA	NA	AA456008	-0.49	NA	NA	NA	NA								
534	DHX36	170506	1	-0.49	NA	NA	NA	NA	NA	NA	223139_s_at	-0.49	NA	NA								
535	KIF23	9493	1	-0.49	NA	NA	NA	NA	AA452513	-0.49	NA	NA	NA	NA								
536	HN1	51155	1	-0.49	NA	NA	NA	NA	AA459865	-0.49	NA	NA	NA	NA								
537	MGC42105	167359	1	-0.49	NA	NA	NA	NA	AA416627	-0.49	NA	NA	NA	NA								
538	NUFIP1	26747	1	-0.49	NA	NA	NA	NA	NA	NA	NA	NA	205134_s_at	-0.49								
539	FLJ20272	55622	1	-0.49	NA	NA	NA	NA	NA	NA	NA	NA	218710_at	-0.49								
540	UBE2D3	7323	1	-0.49	NA	NA	NA	NA	AA017199	-0.49	NA	NA	NA	NA								
541	TMEM65	157378	1	-0.49	NA	NA	NA	NA	NA	NA	NA	NA	238045_at	-0.49								
542	MGC5352	192111	1	-0.49	NA	NA	NA	NA	AA455482	-0.49	NA	NA	NA	NA								
543	DLG7	9787	1	-0.49	NA	NA	NA	NA	W93568	-0.49	NA	NA	NA	NA								
544	HSPA1A	3303	1	-0.50	NA	NA	NA	NA	NA	NA	NA	NA	200800_s_at	-0.50								
545	PRAME	23532	1	-0.50	NA	NA	NA	NA	NA	NA	NA	NA	204086_at	-0.50								
546	ASNS	440	1	-0.50	NA	NA	NA	NA	AA894927	-0.50	NA	NA	NA	NA								
547	C9orf140	89958	1	-0.50	NA	NA	NA	NA	AA088457	-0.50	NA	NA	NA	NA								
548	KIF11	3832	1	-0.50	NA	NA	NA	NA	AA504625	-0.50	NA	NA	NA	NA								
549	NY-REN-41	91057	1	-0.50	NA	NA	NA	NA	NA	NA	NA	NA	226287_at	-0.50								
550	ZC3H8	84524	1	-0.50	NA	NA	NA	NA	NA	NA	223506_at	-0.50	NA	NA								
551	LRRC15	131578	1	-0.50	NA	NA	NA	NA	NA	NA	NA	NA	213909_at	-0.50								
552	PPAPDC1A	196051	1	-0.50	NA	NA	NA	NA	NA	NA	NA	NA	236044_at	-0.50								
553	FOLH1	2346	1	-0.50	NA	NA	NA	NA	NA	NA	NA	NA	215363_x_at	-0.50								
554	CHEK1	1111	1	-0.50	NA	NA	NA	NA	N53057	-0.50	NA	NA	NA	NA								
555	IL1RAP	3556	1	-0.50	NA	NA	NA	NA	NA	NA	NA	NA	210233_at	-0.50								
556	C20orf39	79953	1	-0.51	NA	NA	NA	NA	NA	NA	NA	NA	219310_at	-0.51								
557	KNTC1	9735	1	-0.51	NA	NA	NA	NA	AA157787	-0.51	NA	NA	NA	NA								
558	EIF5A2	56648	1	-0.51	NA	NA	NA	NA	NA	NA	NA	NA	235289_at	-0.51								
559	LOC440156	440156	1	-0.51	NA	NA	NA	NA	NA	NA	NA	NA	242546_at	-0.51								
560	DDEF1	50807	1	-0.51	NA	NA	NA	NA	NA	NA	NA	NA	224791_at	-0.51								
561	CDCA8	55143	1	-0.51	NA	NA	NA	NA	N63744	-0.51	NA	NA	NA	NA								
562	HSPH1	10808	1	-0.51	NA	NA	NA	NA	NA	NA	NA	NA	206976_s_at	-0.51								
563	MLF1IP	79682	1	-0.51	NA	NA	NA	NA	AA431741	-0.51	NA	NA	NA	NA								
564	SAC3D1	29901	1	-0.52	NA	NA	NA	NA	W95346	-0.52	NA	NA	NA	NA								
565	RASEF	158158	1	-0.52	NA	NA	NA	NA	NA	NA	NA	NA	1553185_at	-0.52								
566	C18orf24	220134	1	-0.52	NA	NA	NA	NA	AA121042	-0.52	NA	NA	NA	NA								
567	LMNB2	3999	1	-0.52	NA	NA	NA	NA	AA456868	-0.52	NA	NA	NA	NA								
568	MGC23985	389336	1	-0.52	NA	NA	NA	NA	NA	NA	NA	NA	1554195_a_at	-0.52								
569	CDCA1	83540	1	-0.53	NA	NA	NA	NA	AA421171	-0.53	NA	NA	NA	NA								
570	SLC2A1	6513	1	-0.53	NA	NA	NA	NA	H58872	-0.53	NA	NA	NA	NA								
571	BRWD3	254065	1	-0.53	NA	NA	NA	NA	NA	NA	NA	NA	235156_at	-0.53								
572	KIAA0101	9768	1	-0.53	NA	NA	NA	NA	W68219	-0.53	NA	NA	NA	NA								
573	CDCA5	113130	1	-0.53	NA	NA	NA	NA	N70010	-0.53	NA	NA	NA	NA								
574	NUSAP1	51203	1	-0.53	NA	NA	NA	NA	AA620485	-0.53	NA	NA	NA	NA								
575	MMP11	4320	1	-0.54	NA	NA	NA	NA	NA	NA	NA	NA	203876_s_at	-0.54								
576	SPOCK	6695	1	-0.54	NA	NA	NA	NA	NA	NA	NA	NA	202363_at	-0.54								
577	SLC25A14	9016	1	-0.54	NA	NA	NA	NA	NA	NA	NA	NA	204587_at	-0.54								
578	CD276	80381	1	-0.54	NA	NA	NA	NA	NA	NA	NA	NA	224859_at	-0.54								
579	HOXB7	3217	1	-0.54	NA	NA	NA	NA	NA	NA	NA	NA	216973_s_at	-0.54								
580	UBE2T	29089	1	-0.54	NA	NA	NA	NA	AA460431	-0.54	NA	NA	NA	NA								
581	HSPA5BP1	54972	1	-0.55	NA	NA	NA	NA	NA	NA	NA	NA	218834_s_at	-0.55								
582	LUZP5	54892	1	-0.55	NA	NA	NA	NA	T99311	-0.55	NA	NA	NA	NA								
583	TSN	7247	1	-0.55	NA	NA	NA	NA	AA460927	-0.55	NA	NA	NA	NA								
584	E2-230K	63893	1	-0.55	NA	NA	NA	NA	NA	NA	NA	NA	218141_at	-0.55								
585	UBE2C	11065	1	-0.57	NA	NA	NA	NA	AA430504	-0.57	NA	NA	NA	NA								
586	ASF1B	55723	1	-0.57	NA	NA	NA	NA	N72697	-0.57	NA	NA	NA	NA								
587	C15orf23	90417	1	-0.57	NA	NA	NA	NA	T84975	-0.57	NA	NA	NA	NA								
588	COVA1	10495	1	-0.58	NA	NA	NA	NA	NA	NA	NA	NA	32042_at	-0.58								
589	CEP55	55165	1	-0.59	NA	NA	NA	NA	AA131908	-0.59	NA	NA	NA	NA								
590	COL12A1	1303	1	-0.60	NA	NA	NA	NA	NA	NA	NA	NA	231766_s_at	-0.60								
591	NOX4	50507	1	-0.63	NA	NA	NA	NA	NA	NA	NA	NA	219773_at	-0.63								
592	IGFBP3	3486	1	-0.63	NA	NA	NA	NA	NA	NA	NA	NA	210095_s_at	-0.63								
593	LOC440462	440462	1	-0.64	NA	NA	NA	NA	NA	NA	NA	NA	227452_at	-0.64								
