# Supplementary material for: Loss of aquaporin-4 expression and putative function in non-small cell lung cancer
Source: BMC Cancer. 2011 May 6;11:161. doi: 10.1186/1471-2407-11-161 (PMC3098822; doi:10.1186/1471-2407-11-161)
Supplement: Additional file 4 — Table S4. Validation data of AQP expression in normal lung and NSCLC by qRT-PCR. The qRT-PCR (Taqman) data of normal lung (N) and NSCLC tumor (T) tissues are presented according to the delta-delta Ct method including raw data (Ct values and MAD error), normalized data by ESD housekeeping gene (dCt value and error) and expression values (ddCt, ddCt error, expression level, level error) relative to the median of the normal lung samples. In the case of the different lung cancer cell lines expression values are assigned relative to A549 cell line. Taqman assays of AQP and ESD genes are specified by gene symbol, assay ID and representative sequence. [file 1471-2407-11-161-S4.PDF]

Supplemental Table 4: The qRT-PCR (Taqman) data of normal lung (N) and NSCLC tumor (T) tissues are presented according to the delta-delta Ct method including raw data (Ct values and MAD error), normalized data by ESD housekeeping gene (dCt value and error) and expression values (ddCt, ddCt error, expression level, level error) relative to the median of the normal lung samples. In the case of the different lung cancer cell lines expression values are assigned relative to A549 cell line. Taqman assays of AQP and ESD genes are specified by gene symbol, assay ID and representative sequence.

Gene	Assay_ID Applied Biosystems	Representative sequence	Sample_ID	Ct value	Ct value error	dCt value	dCt value error	ddCt value	ddCt value error	Expression level	Level Error
AQP1	Hs00166067_m1	GCCGCAATGACCTGGCTGATGGTGT	10N	25.0609	0.094416399	-0.325653	0.094450042	-1.065840455	0.093469614	2.093389046	0.135626907
AQP1	Hs00166067_m1	GCCGCAATGACCTGGCTGATGGTGT	10T	27.599087	0.07343549	1.168919	0.073474718	0.428731545	0.074965218	0.742914688	0.038603281
AQP1	Hs00166067_m1	GCCGCAATGACCTGGCTGATGGTGT	113N	28.223402	0.071001381	1.452212	0.103480884	0.712024545	0.102190031	0.61046287	0.043240753
AQP1	Hs00166067_m1	GCCGCAATGACCTGGCTGATGGTGT	113T	31.403582	0.075178511	2.962284	0.075447793	2.222096545	0.07690006	0.214329666	0.011424427
AQP1	Hs00166067_m1	GCCGCAATGACCTGGCTGATGGTGT	136N	28.822012	0.098748835	1.759789	0.101992438	1.019601545	0.100751517	0.493252564	0.034446603
AQP1	Hs00166067_m1	GCCGCAATGACCTGGCTGATGGTGT	136T	29.719463	0.006933399	3.567386	0.068777345	2.827198545	0.070367413	0.140905659	0.00687267
AQP1	Hs00166067_m1	GCCGCAATGACCTGGCTGATGGTGT	137N	27.632318	0.022123485	1.396911	0.023064078	0.656723545	0.027000403	0.634317238	0.011871408
AQP1	Hs00166067_m1	GCCGCAATGACCTGGCTGATGGTGT	137T	31.098694	0.010839174	3.036207	0.024810948	2.296019545	0.028928063	0.203624132	0.00408295
AQP1	Hs00166067_m1	GCCGCAATGACCTGGCTGATGGTGT	147N	27.763155	0.05051122	1.906969	0.050697125	1.166781545	0.051716738	0.44541389	0.01596689
AQP1	Hs00166067_m1	GCCGCAATGACCTGGCTGATGGTGT	147T	27.958466	0.021971642	1.773541	0.02713325	1.033353545	0.030942898	0.488573139	0.010478908
AQP1	Hs00166067_m1	GCCGCAATGACCTGGCTGATGGTGT	148N	27.065504	0.019479221	0.870628	0.041105995	0.130440545	0.042826953	0.913552443	0.027119153
AQP1	Hs00166067_m1	GCCGCAATGACCTGGCTGATGGTGT	148T	25.475163	0.042696784	-0.276327	0.042909318	-1.016514455	0.045414307	2.023025429	0.063682411
AQP1	Hs00166067_m1	GCCGCAATGACCTGGCTGATGGTGT	154N	25.048306	0.075494321	-0.817546	0.081638982	-1.557733455	0.082982968	2.943909771	0.169331955
AQP1	Hs00166067_m1	GCCGCAATGACCTGGCTGATGGTGT	156N	25.08468	0.00746918	-0.997573	0.033307491	-1.737760455	0.03647792	3.335170357	0.08432834
AQP1	Hs00166067_m1	GCCGCAATGACCTGGCTGATGGTGT	161N	26.86643	0.129914203	0.762499	0.130844168	0.022311545	0.128698311	0.984653787	0.087837884
AQP1	Hs00166067_m1	GCCGCAATGACCTGGCTGATGGTGT	161T	30.017546	0.017590708	3.613506	0.017623105	2.873318545	0.0230613	0.136472432	0.002181495
AQP1	Hs00166067_m1	GCCGCAATGACCTGGCTGATGGTGT	165N	27.288319	0.09705489	1.449239	0.101277787	0.709051545	0.100061001	0.611722164	0.042427215
AQP1	Hs00166067_m1	GCCGCAATGACCTGGCTGATGGTGT	165T	30.432392	0.175324534	4.714788	0.175882576	3.974600545	0.176510425	0.063610091	0.007782549
AQP1	Hs00166067_m1	GCCGCAATGACCTGGCTGATGGTGT	169N	29.146627	0.049123848	2.005386	0.049143224	1.265198545	0.050264608	0.416042107	0.014495228
AQP1	Hs00166067_m1	GCCGCAATGACCTGGCTGATGGTGT	169T	27.332592	0.189889926	0.690575	0.190675654	-0.049612455	0.191254947	1.034986862	0.137205959
AQP1	Hs00166067_m1	GCCGCAATGACCTGGCTGATGGTGT	16N	26.04087	0.114340179	0.705325	0.115799243	-0.034862455	0.114110443	1.024459147	0.081029936
AQP1	Hs00166067_m1	GCCGCAATGACCTGGCTGATGGTGT	16T	27.56896	0.159231472	1.0211	0.161255887	0.280912545	0.161940454	0.823070237	0.092388457
AQP1	Hs00166067_m1	GCCGCAATGACCTGGCTGATGGTGT	188N	28.834965	0.055172746	1.68618	0.055412098	0.945992545	0.056144297	0.519072317	0.020200354
AQP1	Hs00166067_m1	GCCGCAATGACCTGGCTGATGGTGT	188T	29.235622	0.090060291	2.90199	0.095326327	2.161802545	0.096479834	0.223476875	0.014944955
AQP1	Hs00166067_m1	GCCGCAATGACCTGGCTGATGGTGT	202N	28.038929	0.054134093	0.954117	0.054892413	0.213929545	0.055654867	0.862185649	0.033260548
AQP1	Hs00166067_m1	GCCGCAATGACCTGGCTGATGGTGT	202T	25.08068	0.173451032	-1.474732	0.174486874	-2.214919455	0.175119727	4.642556457	0.56353089
AQP1	Hs00166067_m1	GCCGCAATGACCTGGCTGATGGTGT	210N	27.07297	0.064692098	0.864668	0.064843797	0.124480545	0.065075681	0.917334278	0.04137822
AQP1	Hs00166067_m1	GCCGCAATGACCTGGCTGATGGTGT	210T	32.182312	0.048161405	5.329958	0.050548255	4.589770545	0.052691325	0.041528036	0.001516722
AQP1	Hs00166067_m1	GCCGCAATGACCTGGCTGATGGTGT	215N	28.15509	0.04777458	1.494545	0.048041238	0.754357545	0.049237211	0.592810314	0.020231806
AQP1	Hs00166067_m1	GCCGCAATGACCTGGCTGATGGTGT	215T	31.749119	0.066703586	6.166649	0.069263195	5.426461545	0.070842359	0.023252642	0.001141802
AQP1	Hs00166067_m1	GCCGCAATGACCTGGCTGATGGTGT	22N	27.30027	0.015248398	1.313578	0.015466214	0.573390545	0.021203315	0.672035549	0.009876919
AQP1	Hs00166067_m1	GCCGCAATGACCTGGCTGATGGTGT	22T	28.671623	0.12510372	3.655926	0.125112315	2.915738545	0.125993417	0.132518112	0.011573069
AQP1	Hs00166067_m1	GCCGCAATGACCTGGCTGATGGTGT	233N	27.019547	0.049871516	0.795823	0.050044462	0.055635545	0.051106353	0.962170488	0.034084143
AQP1	Hs00166067_m1	GCCGCAATGACCTGGCTGATGGTGT	233T	30.051016	0.153879435	4.011183	0.173862542	3.270995545	0.17449766	0.103593433	0.012529891
AQP1	Hs00166067_m1	GCCGCAATGACCTGGCTGATGGTGT	248T	26.566637	0.030739283	0.941877	0.03701956	0.201689545	0.039896084	0.869531655	0.024045905
AQP1	Hs00166067_m1	GCCGCAATGACCTGGCTGATGGTGT	24N	26.123121	0.04287865	1.073381	0.067444705	0.333193545	0.067552016	0.793777434	0.037167429
AQP1	Hs00166067_m1	GCCGCAATGACCTGGCTGATGGTGT	24T	27.75284	0.123585289	3.144194	0.138932567	2.404006545	0.139726547	0.188939134	0.018298956
AQP1	Hs00166067_m1	GCCGCAATGACCTGGCTGATGGTGT	25N	26.396389	0.019456127	1.375961	0.019522171	0.635773545	0.02418763	0.643595639	0.010790259
AQP1	Hs00166067_m1	GCCGCAATGACCTGGCTGATGGTGT	25T	30.528147	0.158975129	5.441935	0.159372136	4.701747545	0.16006476	0.038426688	0.004263381
AQP1	Hs00166067_m1	GCCGCAATGACCTGGCTGATGGTGT	271N	26.579056	0.039113749	0.371816	0.040186998	-0.368371455	0.041986137	1.290894818	0.03756836
AQP1	Hs00166067_m1	GCCGCAATGACCTGGCTGATGGTGT	271T	32.425373	0.251867323	3.753267	0.253991145	3.013079545	0.254426318	0.123871865	0.021845409
AQP1	Hs00166067_m1	GCCGCAATGACCTGGCTGATGGTGT	273N	27.700615	0.035229913	1.163088	0.039613837	0.422900545	0.041462947	0.745923433	0.021437783
AQP1	Hs00166067_m1	GCCGCAATGACCTGGCTGATGGTGT	273T	31.115335	0.009621542	5.426929	0.016845146	4.686741545	0.022472398	0.038828464	0.000604819
AQP1	Hs00166067_m1	GCCGCAATGACCTGGCTGATGGTGT	276N	27.664526	0.010085732	1.566435	0.010490567	0.826247545	0.018063757	0.563994287	0.007061684
AQP1	Hs00166067_m1	GCCGCAATGACCTGGCTGATGGTGT	276T	27.855482	0.191500156	3.59141	0.192845114	2.851222545	0.193417909	0.138578702	0.018578842
AQP1	Hs00166067_m1	GCCGCAATGACCTGGCTGATGGTGT	282N	25.746002	0.109433279	-0.099217	0.134995064	-0.839404455	0.132727422	1.78931136	0.164615998
AQP1	Hs00166067_m1	GCCGCAATGACCTGGCTGATGGTGT	282T	31.888014	0.169887049	4.53402	0.176588095	3.793832545	0.177213445	0.072101219	0.008856553
AQP1	Hs00166067_m1	GCCGCAATGACCTGGCTGATGGTGT	284N	26.051043	0.026561576	-0.219487	0.030022126	-0.959674455	0.033504892	1.944870983	0.045167336
AQP1	Hs00166067_m1	GCCGCAATGACCTGGCTGATGGTGT	301T	28.832182	0.019611434	3.002842	0.021317385	2.262654545	0.025993857	0.208388195	0.003754649
AQP1	Hs00166067_m1	GCCGCAATGACCTGGCTGATGGTGT	30N	25.220455	0.088300528	0.285938	0.088411468	-0.454249455	0.087650088	1.370069857	0.083237788
AQP1	Hs00166067_m1	GCCGCAATGACCTGGCTGATGGTGT	30T	30.291082	0.007466294	5.535766	0.009933721	4.795578545	0.017886546	0.036007006	0.000446415
AQP1	Hs00166067_m1	GCCGCAATGACCTGGCTGATGGTGT	331N	29.036518	0.231379471	2.158724	0.233200502	1.418536545	0.228323878	0.374091594	0.059204503
AQP1	Hs00166067_m1	GCCGCAATGACCTGGCTGATGGTGT	331T	28.318426	0.020256912	2.518602	0.022858476	1.778414545	0.027271957	0.291503569	0.005510432
AQP1	Hs00166067_m1	GCCGCAATGACCTGGCTGATGGTGT	334N	26.978424	0.060731475	1.027328	0.061885098	0.287140545	0.062265037	0.819524767	0.035369734
AQP1	Hs00166067_m1	GCCGCAATGACCTGGCTGATGGTGT	334T	33.862297	0.049420028	6.95664	0.050297415	6.216452545	0.052450736	0.013448112	0.000488921
AQP1	Hs00166067_m1	GCCGCAATGACCTGGCTGATGGTGT	342N	30.3347	0.194672696	1.862982	0.195398722	1.122794545	0.195964053	0.459203472	0.062374494
AQP1	Hs00166067_m1	GCCGCAATGACCTGGCTGATGGTGT	342T	31.870235	0.030532592	4.756076	0.049193132	4.015888545	0.051392742	0.061815458	0.002202036
AQP1	Hs00166067_m1	GCCGCAATGACCTGGCTGATGGTGT	347T	31.58753	0.04214657	4.177965	0.086560599	3.437777545	0.087829306	0.092283879	0.005618117

Gene	Assay_ID Applied Biosystems	Representative sequence	Sample_ID	Ct value	Ct value error	dCt value	dCt value error	ddCt value	ddCt value error	Expression level	Level Error
AQP1	Hs00166067_m1	GCCGCAATGACCTGGCTGATGGTGT	348N	24.791677	0.02144914	-0.946036	0.04977681	-1.686223455	0.051951713	3.218131892	0.11588552
AQP1	Hs00166067_m1	GCCGCAATGACCTGGCTGATGGTGT	364T	28.442198	0.017812411	2.325091	0.026436421	1.584903545	0.030333712	0.333346955	0.007008862
AQP1	Hs00166067_m1	GCCGCAATGACCTGGCTGATGGTGT	368N	28.052698	0.042841699	0.067498	0.043702198	-0.672689455	0.045214146	1.594041793	0.049957362
AQP1	Hs00166067_m1	GCCGCAATGACCTGGCTGATGGTGT	368T	30.487568	0.012544667	3.654902	0.012995299	2.914714545	0.019751645	0.132612204	0.001815567
AQP1	Hs00166067_m1	GCCGCAATGACCTGGCTGATGGTGT	378N	26.682175	0.062390202	0.027703	0.063364696	-0.712484455	0.063669699	1.63862355	0.072316509
AQP1	Hs00166067_m1	GCCGCAATGACCTGGCTGATGGTGT	378T	29.350958	0.021482049	2.621977	0.021967049	1.881789545	0.026529247	0.271346923	0.00498971
AQP1	Hs00166067_m1	GCCGCAATGACCTGGCTGATGGTGT	37N	25.535906	0.093042305	-0.218429	0.095992051	-0.958616455	0.094957274	1.943445236	0.127916335
AQP1	Hs00166067_m1	GCCGCAATGACCTGGCTGATGGTGT	37T	28.82154	0.118771343	3.62319	0.124560997	2.883002545	0.125445971	0.135559437	0.011787235
AQP1	Hs00166067_m1	GCCGCAATGACCTGGCTGATGGTGT	390N	26.227392	0.007414332	0.273813	0.073624441	-0.466374455	0.073453516	1.381633007	0.070344598
AQP1	Hs00166067_m1	GCCGCAATGACCTGGCTGATGGTGT	390T	28.60772	0.019898377	2.454508	0.022165944	1.714320545	0.026694172	0.304746056	0.005638713
AQP1	Hs00166067_m1	GCCGCAATGACCTGGCTGATGGTGT	397T	29.329706	0.000755174	2.874445	0.046151476	2.134257545	0.048489262	0.227784653	0.007655887
AQP1	Hs00166067_m1	GCCGCAATGACCTGGCTGATGGTGT	400T	29.481102	0.053208023	3.369905	0.058176273	2.629717545	0.060047718	0.161575734	0.00672509
AQP1	Hs00166067_m1	GCCGCAATGACCTGGCTGATGGTGT	402T	23.964138	0.056276063	-2.200079	0.064806035	-2.940266455	0.066491142	7.675530434	0.353750978
AQP1	Hs00166067_m1	GCCGCAATGACCTGGCTGATGGTGT	40N	23.601372	0.06368058	-1.15706	0.063770453	-1.897247455	0.064055224	3.725018149	0.165389679
AQP1	Hs00166067_m1	GCCGCAATGACCTGGCTGATGGTGT	40T	29.714287	0.071357029	3.142231	0.072463151	2.402043545	0.073974035	0.189196388	0.009701025
AQP1	Hs00166067_m1	GCCGCAATGACCTGGCTGATGGTGT	412T	28.276833	0.037235051	2.401282	0.037666914	1.661094545	0.040497483	0.316199163	0.008875937
AQP1	Hs00166067_m1	GCCGCAATGACCTGGCTGATGGTGT	421N	27.153479	0.113089639	0.456019	0.114631288	-0.284168455	0.112979175	1.217708195	0.095360186
AQP1	Hs00166067_m1	GCCGCAATGACCTGGCTGATGGTGT	421T	28.768635	0.025947853	2.290419	0.035041003	1.550231545	0.03806733	0.341455258	0.009009728
AQP1	Hs00166067_m1	GCCGCAATGACCTGGCTGATGGTGT	426N	25.837084	0.283805762	-0.106418	0.284250044	-0.846605455	0.278112741	1.798264771	0.346657007
AQP1	Hs00166067_m1	GCCGCAATGACCTGGCTGATGGTGT	426T	29.370306	0.018611463	3.376211	0.018710205	2.636023545	0.023902332	0.16087103	0.002665285
AQP1	Hs00166067_m1	GCCGCAATGACCTGGCTGATGGTGT	42N	25.826664	0.030014708	-0.306068	0.034248845	-1.046255455	0.036618516	2.065162712	0.052418003
AQP1	Hs00166067_m1	GCCGCAATGACCTGGCTGATGGTGT	42T	26.754225	0.191932591	0.712591	0.200115105	-0.027596455	0.200667149	1.019312525	0.141778084
AQP1	Hs00166067_m1	GCCGCAATGACCTGGCTGATGGTGT	434N	26.468037	0.040173186	-0.469791	0.087999996	-1.209978455	0.087253935	2.31334182	0.139910494
AQP1	Hs00166067_m1	GCCGCAATGACCTGGCTGATGGTGT	434T	31.615444	0.124142432	4.38558	0.139318174	3.645392545	0.140109969	0.079914852	0.007761077
AQP1	Hs00166067_m1	GCCGCAATGACCTGGCTGATGGTGT	445N	26.74648	0.094127146	0.033185	0.094636704	-0.707002455	0.093649665	1.63240887	0.105964559
AQP1	Hs00166067_m1	GCCGCAATGACCTGGCTGATGGTGT	445T	26.617764	0.055437173	-0.208772	0.064445359	-0.948959455	0.066139655	1.930479795	0.088501911
AQP1	Hs00166067_m1	GCCGCAATGACCTGGCTGATGGTGT	489T	31.096722	0.125438584	4.888247	0.126896567	4.148059545	0.127765365	0.056403967	0.004995147
AQP1	Hs00166067_m1	GCCGCAATGACCTGGCTGATGGTGT	492N	26.064713	0.252295717	-0.109613	0.255834387	-0.849800455	0.250394543	1.802251631	0.312799283
AQP1	Hs00166067_m1	GCCGCAATGACCTGGCTGATGGTGT	492T	29.219488	0.005537944	1.975281	0.031375078	1.235093545	0.034722402	0.424814953	0.010224334
AQP1	Hs00166067_m1	GCCGCAATGACCTGGCTGATGGTGT	49N	25.973549	0.093480514	0.565598	0.099163595	-0.174589455	0.098018849	1.128643183	0.076681698
AQP1	Hs00166067_m1	GCCGCAATGACCTGGCTGATGGTGT	49T	23.499792	0.018613773	-1.918085	0.018623698	-2.658272455	0.023834677	6.312766787	0.104292838
AQP1	Hs00166067_m1	GCCGCAATGACCTGGCTGATGGTGT	51N	27.113262	0.008929877	1.017708	0.016090639	0.277520545	0.021642315	0.82500768	0.012376195
AQP1	Hs00166067_m1	GCCGCAATGACCTGGCTGATGGTGT	51T	29.128765	0.050842619	2.75794	0.05120091	2.017752545	0.053317754	0.246942568	0.009126269
AQP1	Hs00166067_m1	GCCGCAATGACCTGGCTGATGGTGT	53N	26.992992	0.030253154	1.124036	0.068811419	0.383848545	0.068855162	0.766390432	0.036577333
AQP1	Hs00166067_m1	GCCGCAATGACCTGGCTGATGGTGT	53T	27.286787	0.098708998	1.355767	0.098712607	0.615579545	0.099826993	0.652667658	0.045161207
AQP1	Hs00166067_m1	GCCGCAATGACCTGGCTGATGGTGT	55N	28.974459	0.028279771	1.459423	0.029083274	0.719235545	0.032072408	0.607419216	0.013503476
AQP1	Hs00166067_m1	GCCGCAATGACCTGGCTGATGGTGT	55T	31.878592	0.102479673	6.318011	0.10262873	5.577823545	0.103701042	0.020936679	0.00150493
AQP1	Hs00166067_m1	GCCGCAATGACCTGGCTGATGGTGT	65N	26.90368	0.055154271	-0.275437	0.073278214	-1.015624455	0.074772631	2.021777808	0.104785587
AQP1	Hs00166067_m1	GCCGCAATGACCTGGCTGATGGTGT	70T	28.766336	0.006966308	3.289299	0.024636377	2.549111545	0.028778478	0.170860221	0.003408272
AQP1	Hs00166067_m1	GCCGCAATGACCTGGCTGATGGTGT	75N	27.330404	0.001592332	-0.15037	0.02549879	-0.890557455	0.029520128	1.853892326	0.037933962
AQP1	Hs00166067_m1	GCCGCAATGACCTGGCTGATGGTGT	77N	26.589216	0.022954292	0.451719	0.023575495	-0.288468455	0.027418787	1.221343028	0.023211935
AQP1	Hs00166067_m1	GCCGCAATGACCTGGCTGATGGTGT	77T	32.604794	0.018784668	3.07779	0.041389908	2.337602545	0.043981521	0.197838821	0.006031248
AQP1	Hs00166067_m1	GCCGCAATGACCTGGCTGATGGTGT	84N	26.253931	0.078977475	-0.183584	0.086081588	-0.923771455	0.085407667	1.897068081	0.112306589
AQP1	Hs00166067_m1	GCCGCAATGACCTGGCTGATGGTGT	84T	29.567928	0.037379388	3.759662	0.044663245	3.019474545	0.047074995	0.123323997	0.00402405
AQP1	Hs00166067_m1	GCCGCAATGACCTGGCTGATGGTGT	88N	25.703705	0.00683525	-0.470739	0.059700313	-1.210926455	0.060194446	2.314862424	0.096584418
AQP1	Hs00166067_m1	GCCGCAATGACCTGGCTGATGGTGT	88T	26.304092	0.016719486	0.472055	0.095255496	-0.268132455	0.096409851	1.204247939	0.080475334
AQP1	Hs00166067_m1	GCCGCAATGACCTGGCTGATGGTGT	89T	28.638195	0.025473271	1.523928	0.027276377	0.783740545	0.03106848	0.580858818	0.012508812
AQP1	Hs00166067_m1	GCCGCAATGACCTGGCTGATGGTGT	95N	28.924604	0.115583792	2.097578	0.115779299	1.357390545	0.114091124	0.390287579	0.030864699
AQP1	Hs00166067_m1	GCCGCAATGACCTGGCTGATGGTGT	95T	30.360828	0.131734588	3.803421	0.132091314	3.063233545	0.132926164	0.119639563	0.011023278
AQP1	Hs00166067_m1	GCCGCAATGACCTGGCTGATGGTGT	97N	24.753626	0.034926227	-1.184481	0.036672269	-1.924668455	0.039574045	3.796495912	0.104140304
AQP1	Hs00166067_m1	GCCGCAATGACCTGGCTGATGGTGT	NTC	NA	NA	NA	NA	NA	NA	NA	NA

Gene	Assay_ID Applied Biosystems	Representative sequence	Sample_ID	Ct value	Ct value error	dCt value	dCt value error	ddCt value	ddCt value error	Expression level	Level Error
AQP3	Hs00185020_m1	GGCCAGGTCTCTGGGGCCCACCTGA	10N	25.679596	0.032728832	0.293043	0.032825759	-0.390957977	0.032685176	1.311263819	0.029707518
AQP3	Hs00185020_m1	GGCCAGGTCTCTGGGGCCCACCTGA	10T	26.331593	0.006386649	-0.098575	0.006822922	-0.782575977	0.009290921	1.720199603	0.011078044
AQP3	Hs00185020_m1	GGCCAGGTCTCTGGGGCCCACCTGA	113N	27.560854	0.000900666	0.789664	0.075285512	0.105663023	0.07382442	0.929377728	0.047557363
AQP3	Hs00185020_m1	GGCCAGGTCTCTGGGGCCCACCTGA	113T	30.08087	0.00696169	1.639572	0.009435365	0.955571023	0.011348791	0.515637459	0.004056201
AQP3	Hs00185020_m1	GGCCAGGTCTCTGGGGCCCACCTGA	136N	28.799833	0.012999041	1.73761	0.028637388	1.053609023	0.028680859	0.481761489	0.009577446
AQP3	Hs00185020_m1	GGCCAGGTCTCTGGGGCCCACCTGA	136T	26.325686	0.028359445	0.173609	0.074070974	-0.510391977	0.074338941	1.42443716	0.073398152
AQP3	Hs00185020_m1	GGCCAGGTCTCTGGGGCCCACCTGA	137N	26.786644	0.013494985	0.551237	0.014987252	-0.132763977	0.015942922	1.096392206	0.012116002
AQP3	Hs00185020_m1	GGCCAGGTCTCTGGGGCCCACCTGA	137T	26.695753	0.039904719	-1.366734	0.045721789	-2.050734977	0.046154642	4.143169885	0.132548124
AQP3	Hs00185020_m1	GGCCAGGTCTCTGGGGCCCACCTGA	147N	27.749052	0.007233622	1.892866	0.008434473	1.208865023	0.010376687	0.432608818	0.00311157
AQP3	Hs00185020_m1	GGCCAGGTCTCTGGGGCCCACCTGA	147T	27.34554	0.009963334	1.160615	0.01878106	0.476614023	0.019811541	0.718662333	0.009868897
AQP3	Hs00185020_m1	GGCCAGGTCTCTGGGGCCCACCTGA	148N	26.11253	0.0160411	-0.082346	0.039592672	-0.766346977	0.039193048	1.700957362	0.046209144
AQP3	Hs00185020_m1	GGCCAGGTCTCTGGGGCCCACCTGA	148T	24.298502	0.002748765	-1.452988	0.005074435	-2.136988977	0.008094371	4.398430991	0.024677793
AQP3	Hs00185020_m1	GGCCAGGTCTCTGGGGCCCACCTGA	154N	26.429314	0.020622374	0.563462	0.037293607	-0.120538977	0.037823036	1.087140932	0.028501499
AQP3	Hs00185020_m1	GGCCAGGTCTCTGGGGCCCACCTGA	156N	26.829172	0.007679913	0.746919	0.03335538	0.062918023	0.033946285	0.957325854	0.022525659
AQP3	Hs00185020_m1	GGCCAGGTCTCTGGGGCCCACCTGA	161N	25.511814	0.01388181	-0.592117	0.020861469	-1.276117977	0.021335135	2.421864209	0.03581547
AQP3	Hs00185020_m1	GGCCAGGTCTCTGGGGCCCACCTGA	161T	26.625051	0.011738108	0.221011	0.011786603	-0.462989977	0.013367609	1.378395577	0.012771828
AQP3	Hs00185020_m1	GGCCAGGTCTCTGGGGCCCACCTGA	165N	27.08618	0.024846846	1.2471	0.038143209	0.563099023	0.037796049	0.676846681	0.017732181
AQP3	Hs00185020_m1	GGCCAGGTCTCTGGGGCCCACCTGA	165T	29.735466	0.039864881	4.017862	0.042251595	3.333861023	0.042719623	0.099176284	0.002936708
AQP3	Hs00185020_m1	GGCCAGGTCTCTGGGGCCCACCTGA	169N	27.979807	0.006170142	0.838566	0.006322554	0.154565023	0.008827605	0.898403204	0.005497176
AQP3	Hs00185020_m1	GGCCAGGTCTCTGGGGCCCACCTGA	169T	26.478489	0.022622893	-0.163528	0.028474832	-0.847528977	0.029164791	1.799416276	0.036376086
AQP3	Hs00185020_m1	GGCCAGGTCTCTGGGGCCCACCTGA	16N	26.559757	0.010580521	1.224212	0.021159761	0.540211023	0.021613718	0.687670316	0.010302324
AQP3	Hs00185020_m1	GGCCAGGTCTCTGGGGCCCACCTGA	16T	27.979977	0.025867024	1.432117	0.036302923	0.748116023	0.03684659	0.595380542	0.015206084
AQP3	Hs00185020_m1	GGCCAGGTCTCTGGGGCCCACCTGA	188N	28.358772	0.050791813	1.209987	0.051051708	0.525986023	0.05027503	0.694484296	0.024201386
AQP3	Hs00185020_m1	GGCCAGGTCTCTGGGGCCCACCTGA	188T	29.213108	0.004751593	2.879476	0.031604276	2.195475023	0.032227306	0.218321328	0.00487692
AQP3	Hs00185020_m1	GGCCAGGTCTCTGGGGCCCACCTGA	202N	25.683023	0.049392316	-1.401789	0.050222284	-2.085789977	0.049471181	4.245074807	0.145567053
AQP3	Hs00185020_m1	GGCCAGGTCTCTGGGGCCCACCTGA	202T	23.671185	0.008946042	-2.884227	0.02098667	-3.568227977	0.021913678	11.86161032	0.180170789
AQP3	Hs00185020_m1	GGCCAGGTCTCTGGGGCCCACCTGA	210N	25.64922	0.002021303	-0.559082	0.004871984	-1.243082977	0.007901029	2.367038181	0.012963264
AQP3	Hs00185020_m1	GGCCAGGTCTCTGGGGCCCACCTGA	210T	29.1333	0.005825464	2.280946	0.016417709	1.596945023	0.017587214	0.330576248	0.004029899
AQP3	Hs00185020_m1	GGCCAGGTCTCTGGGGCCCACCTGA	215N	28.067947	0.012725955	1.407402	0.013693061	0.723401023	0.014790069	0.605667951	0.006209123
AQP3	Hs00185020_m1	GGCCAGGTCTCTGGGGCCCACCTGA	215T	25.804293	0.00264946	0.221823	0.018842543	-0.462177977	0.019869836	1.377619985	0.018973575
AQP3	Hs00185020_m1	GGCCAGGTCTCTGGGGCCCACCTGA	22N	27.522738	0.022994129	1.536046	0.023139147	0.852045023	0.023470232	0.553998885	0.009012634
AQP3	Hs00185020_m1	GGCCAGGTCTCTGGGGCCCACCTGA	22T	27.955511	0.001967032	2.939814	0.002453518	2.255813023	0.006766734	0.209378756	0.000982058
AQP3	Hs00185020_m1	GGCCAGGTCTCTGGGGCCCACCTGA	233N	26.770727	0.015359249	0.547003	0.015911836	-0.136997977	0.016776382	1.099614608	0.012786871
AQP3	Hs00185020_m1	GGCCAGGTCTCTGGGGCCCACCTGA	233T	28.283745	0.087282082	2.243912	0.11902716	1.559911023	0.119194101	0.339171999	0.02802207
AQP3	Hs00185020_m1	GGCCAGGTCTCTGGGGCCCACCTGA	248T	27.009481	0.005256774	1.384721	0.021287977	0.700720023	0.022202408	0.615265062	0.009468644
AQP3	Hs00185020_m1	GGCCAGGTCTCTGGGGCCCACCTGA	24N	24.638865	0.010913075	-0.410875	0.05319121	-1.094875977	0.052349489	2.135947189	0.077504768
AQP3	Hs00185020_m1	GGCCAGGTCTCTGGGGCCCACCTGA	24T	21.849472	0.003744116	-2.759174	0.063584219	-3.443174977	0.063896181	10.87674504	0.481725136
AQP3	Hs00185020_m1	GGCCAGGTCTCTGGGGCCCACCTGA	25N	26.359932	0.00526428	1.339504	0.005503355	0.655503023	0.008287291	0.634854099	0.0036468
AQP3	Hs00185020_m1	GGCCAGGTCTCTGGGGCCCACCTGA	25T	30.113876	0.022491834	5.027664	0.025144957	4.343663023	0.025923692	0.049252371	0.000885013
AQP3	Hs00185020_m1	GGCCAGGTCTCTGGGGCCCACCTGA	271N	27.935167	0.034321164	1.727927	0.03553944	1.043926023	0.035290357	0.485005824	0.011863927
AQP3	Hs00185020_m1	GGCCAGGTCTCTGGGGCCCACCTGA	271T	27.698738	0.005017174	-0.973368	0.033159092	-1.657368977	0.033753434	3.15440735	0.073800822
AQP3	Hs00185020_m1	GGCCAGGTCTCTGGGGCCCACCTGA	273N	27.072752	0.043741211	0.535225	0.047343456	-0.148775977	0.046682865	1.108628482	0.035873107
AQP3	Hs00185020_m1	GGCCAGGTCTCTGGGGCCCACCTGA	273T	22.502596	0.000887387	-3.18581	0.013855408	-3.869810977	0.015223051	14.61938764	0.154261068
AQP3	Hs00185020_m1	GGCCAGGTCTCTGGGGCCCACCTGA	276N	28.159279	0.049891146	2.061188	0.049974558	1.377187023	0.049231135	0.384968679	0.013136834
AQP3	Hs00185020_m1	GGCCAGGTCTCTGGGGCCCACCTGA	276T	27.23221	0.02268236	2.968138	0.032115691	2.284137023	0.032728986	0.205308175	0.004657622
AQP3	Hs00185020_m1	GGCCAGGTCTCTGGGGCCCACCTGA	282N	26.932394	0.080876958	1.087175	0.113088934	0.403174023	0.110668663	0.756192779	0.0580073
AQP3	Hs00185020_m1	GGCCAGGTCTCTGGGGCCCACCTGA	282T	28.214518	0.003994687	0.860524	0.048349803	0.176523023	0.048759331	0.884832926	0.029905046
AQP3	Hs00185020_m1	GGCCAGGTCTCTGGGGCCCACCTGA	284N	27.243263	0.011415947	0.972733	0.018059196	0.288732023	0.019128605	0.818621224	0.010854048
AQP3	Hs00185020_m1	GGCCAGGTCTCTGGGGCCCACCTGA	301T	30.335909	0.017381707	4.506569	0.01928591	3.822568023	0.020290768	0.07067932	0.000994068
AQP3	Hs00185020_m1	GGCCAGGTCTCTGGGGCCCACCTGA	30N	25.998587	0.01057417	1.06407	0.011463751	0.380069023	0.012853521	0.768400827	0.006845977
AQP3	Hs00185020_m1	GGCCAGGTCTCTGGGGCCCACCTGA	30T	25.403727	0.010966768	0.648411	0.012775104	-0.035589977	0.014246832	1.024975892	0.010121792
AQP3	Hs00185020_m1	GGCCAGGTCTCTGGGGCCCACCTGA	331N	27.00814	0.013878346	0.130346	0.032227675	-0.553654977	0.03211202	1.467799568	0.032670806
AQP3	Hs00185020_m1	GGCCAGGTCTCTGGGGCCCACCTGA	331T	25.743526	0.01189688	-0.056298	0.015928063	-0.740298977	0.017131028	1.670521994	0.01983632
AQP3	Hs00185020_m1	GGCCAGGTCTCTGGGGCCCACCTGA	334N	26.891314	0.044331263	0.940218	0.045898957	0.256217023	0.045284914	0.837280527	0.026281491
AQP3	Hs00185020_m1	GGCCAGGTCTCTGGGGCCCACCTGA	334T	27.941072	0.02473484	1.035415	0.02644434	0.351414023	0.02718588	0.783815483	0.014770075
AQP3	Hs00185020_m1	GGCCAGGTCTCTGGGGCCCACCTGA	342N	29.042725	0.027613509	0.571007	0.032337406	-0.112993977	0.032946575	1.081470249	0.024697347
AQP3	Hs00185020_m1	GGCCAGGTCTCTGGGGCCCACCTGA	342T	31.326231	0.001292687	4.212072	0.038592695	3.528071023	0.03910454	0.086685168	0.002349619
AQP3	Hs00185020_m1	GGCCAGGTCTCTGGGGCCCACCTGA	347T	31.819992	0.005392452	4.410427	0.075798961	3.726426023	0.076060841	0.075549917	0.003983094
AQP3	Hs00185020_m1	GGCCAGGTCTCTGGGGCCCACCTGA	348N	26.25392	0.016192943	0.516207	0.047748053	-0.167793977	0.048162699	1.123339474	0.037501384
AQP3	Hs00185020_m1	GGCCAGGTCTCTGGGGCCCACCTGA	364T	26.05568	0.00454952	-0.061427	0.020057431	-0.745427977	0.021025449	1.676471523	0.024432444
AQP3	Hs00185020_m1	GGCCAGGTCTCTGGGGCCCACCTGA	368N	29.201563	0.009250306	1.216363	0.012650656	0.532362023	0.013875646	0.691421791	0.006650001
AQP3	Hs00185020_m1	GGCCAGGTCTCTGGGGCCCACCTGA	368T	23.584898	0.011510055	-3.247768	0.011999604	-3.931768977	0.01355579	15.26090884	0.143393903

Gene	Assay_ID Applied Biosystems	Representative sequence	Sample_ID	Ct value	Ct value error	dCt value	dCt value error	ddCt value	ddCt value error	Expression level	Level Error
AQP3	Hs00185020_m1	GGCCAGGTCTCTGGGGCCCACCTGA	378N	27.350563	0.031817196	0.696091	0.033688001	0.012090023	0.033512159	0.991654851	0.02303501
AQP3	Hs00185020_m1	GGCCAGGTCTCTGGGGCCCACCTGA	378T	24.603838	0.014345422	-2.125143	0.015062003	-2.809143977	0.016328897	7.008685935	0.079326613
AQP3	Hs00185020_m1	GGCCAGGTCTCTGGGGCCCACCTGA	37N	26.184093	0.013840241	0.429758	0.027370707	-0.254242977	0.027474934	1.19270973	0.022714171
AQP3	Hs00185020_m1	GGCCAGGTCTCTGGGGCCCACCTGA	37T	26.061157	0.024592235	0.862807	0.044873021	0.178806023	0.045313982	0.883433825	0.027748002
AQP3	Hs00185020_m1	GGCCAGGTCTCTGGGGCCCACCTGA	390N	27.021656	0.020607941	1.068077	0.076093845	0.384076023	0.074611313	0.766269601	0.039628874
AQP3	Hs00185020_m1	GGCCAGGTCTCTGGGGCCCACCTGA	390T	24.567455	0.009324784	-1.585757	0.013503158	-2.269757977	0.014903162	4.822422246	0.04981603
AQP3	Hs00185020_m1	GGCCAGGTCTCTGGGGCCCACCTGA	397T	27.572765	0.000806558	1.117504	0.046152346	0.433503023	0.046581198	0.740461678	0.02390775
AQP3	Hs00185020_m1	GGCCAGGTCTCTGGGGCCCACCTGA	400T	26.720148	0.029794161	0.608951	0.03796152	-0.075049977	0.038481761	1.053397527	0.028097825
AQP3	Hs00185020_m1	GGCCAGGTCTCTGGGGCCCACCTGA	402T	25.730202	0.018644372	-0.434015	0.037154267	-1.118015977	0.037685654	2.170482781	0.056696711
AQP3	Hs00185020_m1	GGCCAGGTCTCTGGGGCCCACCTGA	40N	24.991577	0.010064947	0.233145	0.010618734	-0.450855977	0.012140886	1.366850994	0.011502627
AQP3	Hs00185020_m1	GGCCAGGTCTCTGGGGCCCACCTGA	40T	25.983736	0.003755086	-0.58832	0.01315991	-1.272320977	0.014592881	2.415498534	0.024432803
AQP3	Hs00185020_m1	GGCCAGGTCTCTGGGGCCCACCTGA	412T	28.592485	0.052437838	2.716934	0.052745372	2.032933023	0.053121024	0.244357786	0.008997422
AQP3	Hs00185020_m1	GGCCAGGTCTCTGGGGCCCACCTGA	421N	26.880487	0.035346538	0.183027	0.040005543	-0.500973977	0.039591224	1.415168634	0.038835829
AQP3	Hs00185020_m1	GGCCAGGTCTCTGGGGCCCACCTGA	421T	28.644611	0.019777711	2.166395	0.030752865	1.482394023	0.031392796	0.357894425	0.007787721
AQP3	Hs00185020_m1	GGCCAGGTCTCTGGGGCCCACCTGA	426N	26.748709	0.007610054	0.805207	0.017615041	0.121206023	0.018329063	0.91941874	0.011680974
AQP3	Hs00185020_m1	GGCCAGGTCTCTGGGGCCCACCTGA	426T	24.850327	0.008061542	-1.143768	0.008286958	-1.827768977	0.010413578	3.549876852	0.025623516
AQP3	Hs00185020_m1	GGCCAGGTCTCTGGGGCCCACCTGA	42N	26.658842	0.029685041	0.52611	0.033960306	-0.157890977	0.033773488	1.11565501	0.026117481
AQP3	Hs00185020_m1	GGCCAGGTCTCTGGGGCCCACCTGA	42T	24.548618	0.027045396	-1.493016	0.062764551	-2.177016977	0.063080566	4.522175484	0.197728127
AQP3	Hs00185020_m1	GGCCAGGTCTCTGGGGCCCACCTGA	434N	27.486965	0.037657671	0.549137	0.086880462	-0.134863977	0.085116878	1.097989286	0.064779747
AQP3	Hs00185020_m1	GGCCAGGTCTCTGGGGCCCACCTGA	434T	27.339798	0.013474201	0.109934	0.064651096	-0.574066977	0.064957934	1.488714367	0.067029973
AQP3	Hs00185020_m1	GGCCAGGTCTCTGGGGCCCACCTGA	445N	26.557766	0.023246431	-0.155529	0.025230589	-0.839529977	0.025444371	1.789467048	0.031560282
AQP3	Hs00185020_m1	GGCCAGGTCTCTGGGGCCCACCTGA	445T	24.784311	0.01660979	-2.042225	0.036821316	-2.726225977	0.037357439	6.617223359	0.171347729
AQP3	Hs00185020_m1	GGCCAGGTCTCTGGGGCCCACCTGA	489T	27.133127	0.003148291	0.924652	0.019437391	0.240651023	0.020434801	0.846363301	0.011988165
AQP3	Hs00185020_m1	GGCCAGGTCTCTGGGGCCCACCTGA	492N	26.840853	0.003114227	0.666527	0.042518271	-0.017473977	0.042016657	1.012185686	0.02947862
AQP3	Hs00185020_m1	GGCCAGGTCTCTGGGGCCCACCTGA	492T	25.38747	0.010516435	-1.856737	0.03262395	-2.540737977	0.033227866	5.818865814	0.134018962
AQP3	Hs00185020_m1	GGCCAGGTCTCTGGGGCCCACCTGA	49N	26.065088	0.001305966	0.657137	0.033113707	-0.026863977	0.032961262	1.018795136	0.023276419
AQP3	Hs00185020_m1	GGCCAGGTCTCTGGGGCCCACCTGA	49T	22.825624	0.001682976	-2.592253	0.001789416	-3.276253977	0.006555224	9.688370097	0.044021384
AQP3	Hs00185020_m1	GGCCAGGTCTCTGGGGCCCACCTGA	51N	26.487083	0.007960506	0.391529	0.015573555	-0.292471977	0.016470586	1.224736999	0.013982259
AQP3	Hs00185020_m1	GGCCAGGTCTCTGGGGCCCACCTGA	51T	25.18048	0.009778004	-1.190345	0.011496548	-1.874345977	0.013112572	3.666353713	0.033323278
AQP3	Hs00185020_m1	GGCCAGGTCTCTGGGGCCCACCTGA	53N	25.809145	0.011959811	-0.059811	0.062950736	-0.743811977	0.061825861	1.674594715	0.071763787
AQP3	Hs00185020_m1	GGCCAGGTCTCTGGGGCCCACCTGA	53T	23.731792	0.000209001	-2.199228	0.000869576	-2.883228977	0.006365933	7.377995849	0.032555619
AQP3	Hs00185020_m1	GGCCAGGTCTCTGGGGCCCACCTGA	55N	28.298817	0.014524401	0.783781	0.016032766	0.099780023	0.016885925	0.933175268	0.010922286
AQP3	Hs00185020_m1	GGCCAGGTCTCTGGGGCCCACCTGA	55T	31.074942	0.01882797	5.514361	0.019623084	4.830360023	0.02061151	0.035149305	0.000502171
AQP3	Hs00185020_m1	GGCCAGGTCTCTGGGGCCCACCTGA	65N	27.997084	0.008101956	0.817967	0.048921823	0.133966023	0.049326603	0.911322743	0.031158667
AQP3	Hs00185020_m1	GGCCAGGTCTCTGGGGCCCACCTGA	70T	25.890272	0.023200243	0.413235	0.033116052	-0.270765977	0.033711153	1.206448203	0.028190823
AQP3	Hs00185020_m1	GGCCAGGTCTCTGGGGCCCACCTGA	75N	29.011839	0.026040229	1.531065	0.036410799	0.847064023	0.036952878	0.555914908	0.014239084
AQP3	Hs00185020_m1	GGCCAGGTCTCTGGGGCCCACCTGA	77N	26.545424	0.015121958	0.407927	0.016049239	-0.276073977	0.016900856	1.210895174	0.014185371
AQP3	Hs00185020_m1	GGCCAGGTCTCTGGGGCCCACCTGA	77T	33.542465	0.06102015	4.015461	0.071300206	3.331460023	0.071578547	0.099341475	0.004928774
AQP3	Hs00185020_m1	GGCCAGGTCTCTGGGGCCCACCTGA	84N	26.961483	0.048560931	0.523968	0.059420218	-0.160032977	0.058395565	1.117312677	0.045225154
AQP3	Hs00185020_m1	GGCCAGGTCTCTGGGGCCCACCTGA	84T	29.452267	0.006731904	3.644001	0.025355577	2.960000023	0.026128036	0.128514226	0.002327466
AQP3	Hs00185020_m1	GGCCAGGTCTCTGGGGCCCACCTGA	88N	27.404505	0.004060504	1.230061	0.059446568	0.546060023	0.058421159	0.684887995	0.027734171
AQP3	Hs00185020_m1	GGCCAGGTCTCTGGGGCCCACCTGA	88T	24.727549	0.005184028	-1.104488	0.093919874	-1.788488977	0.094131353	3.45452889	0.225397238
AQP3	Hs00185020_m1	GGCCAGGTCTCTGGGGCCCACCTGA	89T	28.948338	0.034127752	1.834071	0.035493896	1.150070023	0.036049766	0.45060336	0.011259584
AQP3	Hs00185020_m1	GGCCAGGTCTCTGGGGCCCACCTGA	95N	27.656384	0.014146236	0.829358	0.015663622	0.145357023	0.016551912	0.904155599	0.010373297
AQP3	Hs00185020_m1	GGCCAGGTCTCTGGGGCCCACCTGA	95T	28.618586	0.015017458	2.061179	0.017878413	1.377178023	0.018958022	0.38497108	0.005058789
AQP3	Hs00185020_m1	GGCCAGGTCTCTGGGGCCCACCTGA	97N	25.962002	0.003075545	0.023895	0.011596248	-0.660105977	0.013200072	1.580198697	0.014458175
AQP3	Hs00185020_m1	GGCCAGGTCTCTGGGGCCCACCTGA	NTC	NA	NA	NA	NA	NA	NA	NA	NA

Gene	Assay_ID Applied Biosystems	Representative sequence	Sample_ID	Ct value	Ct value error	dCt value	dCt value error	ddCt value	ddCt value error	Expression level	Level Error
AQP4	Hs00242342_m1	GCCTGGGAGTCACCATGGTTCATGG	10N	26.806513	0.003039172	1.41996	0.003948487	-0.106690341	0.007927349	1.076755237	0.005916576
AQP4	Hs00242342_m1	GCCTGGGAGTCACCATGGTTCATGG	10T	31.12748	0.008375043	4.697312	0.008712309	3.170661659	0.011129479	0.111054391	0.000856714
AQP4	Hs00242342_m1	GCCTGGGAGTCACCATGGTTCATGG	113N	29.063183	0.030141148	2.291993	0.081089987	0.765342659	0.07952771	0.58831362	0.03243044
AQP4	Hs00242342_m1	GCCTGGGAGTCACCATGGTTCATGG	113T	34.297512	0.045032166	5.856214	0.045480292	4.329563659	0.046004543	0.049736071	0.00158598
AQP4	Hs00242342_m1	GCCTGGGAGTCACCATGGTTCATGG	136N	29.351986	0.03133049	2.289763	0.040406986	0.763112659	0.040080803	0.58922369	0.016369751
AQP4	Hs00242342_m1	GCCTGGGAGTCACCATGGTTCATGG	136T	28.541697	0.007689728	2.38962	0.068857701	0.862969659	0.069205087	0.549819638	0.026374469
AQP4	Hs00242342_m1	GCCTGGGAGTCACCATGGTTCATGG	137N	28.613369	0.004897085	2.377962	0.008153805	0.851311659	0.010555732	0.55428057	0.004055491
AQP4	Hs00242342_m1	GCCTGGGAGTCACCATGGTTCATGG	137T	31.87471	0.065184	3.812223	0.068898834	2.285572659	0.069246013	0.205103973	0.009844515
AQP4	Hs00242342_m1	GCCTGGGAGTCACCATGGTTCATGG	147N	27.713202	0.006814465	1.857016	0.008077871	0.330365659	0.010499855	0.795334876	0.005788404
AQP4	Hs00242342_m1	GCCTGGGAGTCACCATGGTTCATGG	147T	27.74498	0.007100831	1.560055	0.017432212	0.033404659	0.018757478	0.97711166	0.012704106
AQP4	Hs00242342_m1	GCCTGGGAGTCACCATGGTTCATGG	148N	27.243397	0.015006488	1.048521	0.039184914	-0.478129341	0.038905333	1.392936355	0.037563485
AQP4	Hs00242342_m1	GCCTGGGAGTCACCATGGTTCATGG	148T	26.478771	0.000899512	0.727281	0.004359278	-0.799369341	0.00818317	1.740340189	0.009871455
AQP4	Hs00242342_m1	GCCTGGGAGTCACCATGGTTCATGG	154N	27.10528	0.001465892	1.239428	0.03110755	-0.287222341	0.031869117	1.220288561	0.02695616
AQP4	Hs00242342_m1	GCCTGGGAGTCACCATGGTTCATGG	156N	27.559685	0.007894688	1.477432	0.033405484	-0.049218341	0.034115793	1.034704164	0.024467924
AQP4	Hs00242342_m1	GCCTGGGAGTCACCATGGTTCATGG	161N	27.278381	0.003338817	1.17445	0.015926203	-0.352200341	0.017031608	1.276506019	0.015069678
AQP4	Hs00242342_m1	GCCTGGGAGTCACCATGGTTCATGG	161T	30.982227	0.002755115	4.578187	0.00295491	3.051536659	0.00752944	0.120613504	0.000629483
AQP4	Hs00242342_m1	GCCTGGGAGTCACCATGGTTCATGG	165N	27.521318	0.01214745	1.682238	0.031386289	0.155587659	0.031436967	0.897766607	0.019562734
AQP4	Hs00242342_m1	GCCTGGGAGTCACCATGGTTCATGG	165T	31.979195	0.013196495	6.261591	0.019238918	4.734940659	0.020447419	0.037552671	0.000532237
AQP4	Hs00242342_m1	GCCTGGGAGTCACCATGGTTCATGG	169N	29.38583	0.044624557	2.244589	0.044645886	0.717938659	0.044165751	0.607965491	0.01861187
AQP4	Hs00242342_m1	GCCTGGGAGTCACCATGGTTCATGG	169T	26.674225	0.01328483	0.032208	0.021806134	-1.494442341	0.022879433	2.817552204	0.044683038
AQP4	Hs00242342_m1	GCCTGGGAGTCACCATGGTTCATGG	16N	26.951103	0.013224208	1.615558	0.022597958	0.088907659	0.023139069	0.940234381	0.015080213
AQP4	Hs00242342_m1	GCCTGGGAGTCACCATGGTTCATGG	16T	29.872393	0.024356676	3.324533	0.035242687	1.797882659	0.03591668	0.287596364	0.007159868
AQP4	Hs00242342_m1	GCCTGGGAGTCACCATGGTTCATGG	188N	28.963305	0.042003387	1.81452	0.042317291	0.287869659	0.041920356	0.819110697	0.02380088
AQP4	Hs00242342_m1	GCCTGGGAGTCACCATGGTTCATGG	188T	29.593786	0.008568455	3.260154	0.032398628	1.733503659	0.03313053	0.300720754	0.006905852
AQP4	Hs00242342_m1	GCCTGGGAGTCACCATGGTTCATGG	202N	29.85949	0.018793329	2.774678	0.020877409	1.248027659	0.021541009	0.421023405	0.006286338
AQP4	Hs00242342_m1	GCCTGGGAGTCACCATGGTTCATGG	202T	27.908007	0.042350374	1.352595	0.046410805	-0.174055341	0.046924661	1.128225415	0.036696317
AQP4	Hs00242342_m1	GCCTGGGAGTCACCATGGTTCATGG	210N	28.217997	0.00046188	2.009695	0.004456893	0.483044659	0.008180584	0.715466112	0.004056943
AQP4	Hs00242342_m1	GCCTGGGAGTCACCATGGTTCATGG	210T	34.60424	0.036762778	7.751886	0.039838512	6.225235659	0.040435974	0.013366489	0.000374637
AQP4	Hs00242342_m1	GCCTGGGAGTCACCATGGTTCATGG	215N	28.752512	0.016643854	2.091967	0.017394478	0.565316659	0.018351451	0.675807066	0.008596439
AQP4	Hs00242342_m1	GCCTGGGAGTCACCATGGTTCATGG	215T	34.96064	0.219834198	9.37817	0.220624333	7.851519659	0.220733	0.004329693	0.000662445
AQP4	Hs00242342_m1	GCCTGGGAGTCACCATGGTTCATGG	22N	28.043854	0.013300418	2.057162	0.013549585	0.530511659	0.014940119	0.69230916	0.007169347
AQP4	Hs00242342_m1	GCCTGGGAGTCACCATGGTTCATGG	22T	30.153023	0.013469582	5.137326	0.013549176	3.610675659	0.015216476	0.081861241	0.000863412
AQP4	Hs00242342_m1	GCCTGGGAGTCACCATGGTTCATGG	233N	27.217651	0.03111687	0.993927	0.031393305	-0.532723341	0.031443653	1.44665744	0.031530014
AQP4	Hs00242342_m1	GCCTGGGAGTCACCATGGTTCATGG	233T	33.60225	0.000517306	7.562417	0.080929418	6.035766659	0.081225191	0.015242394	0.000858162
AQP4	Hs00242342_m1	GCCTGGGAGTCACCATGGTTCATGG	248T	28.26695	0.012687272	2.64219	0.024217993	1.115539659	0.02518873	0.461518486	0.008057881
AQP4	Hs00242342_m1	GCCTGGGAGTCACCATGGTTCATGG	24N	25.993048	0.01592332	0.943308	0.054440442	-0.583342341	0.053637732	1.498316432	0.055705671
AQP4	Hs00242342_m1	GCCTGGGAGTCACCATGGTTCATGG	24T	29.501408	0.023528178	4.892762	0.067694237	3.366111659	0.068047562	0.09698385	0.004574435
AQP4	Hs00242342_m1	GCCTGGGAGTCACCATGGTTCATGG	25N	25.901434	0.023410399	0.881006	0.023465316	-0.645644341	0.023948983	1.564437844	0.025969934
AQP4	Hs00242342_m1	GCCTGGGAGTCACCATGGTTCATGG	25T	38.044304	0.677259804	12.958092	0.677353105	11.43144166	0.677388507	0.000362071	0.000170003
AQP4	Hs00242342_m1	GCCTGGGAGTCACCATGGTTCATGG	271N	27.592678	0.010285495	1.385438	0.013816689	-0.141212341	0.015171834	1.10283147	0.011597722
AQP4	Hs00242342_m1	GCCTGGGAGTCACCATGGTTCATGG	271T	35.317974	0.039154741	6.645868	0.051063167	5.119217659	0.051530651	0.028771462	0.001027668
AQP4	Hs00242342_m1	GCCTGGGAGTCACCATGGTTCATGG	273N	28.06133	0.035297463	1.523803	0.039673924	-0.002847341	0.039375561	1.001975575	0.027346979
AQP4	Hs00242342_m1	GCCTGGGAGTCACCATGGTTCATGG	273T	29.640196	0.023789718	3.95179	0.027516096	2.425139659	0.028374223	0.186191658	0.003661927
AQP4	Hs00242342_m1	GCCTGGGAGTCACCATGGTTCATGG	276N	27.49596	0.007824828	1.397869	0.00834014	-0.128781341	0.010693792	1.093369731	0.008104463
AQP4	Hs00242342_m1	GCCTGGGAGTCACCATGGTTCATGG	276T	27.541346	0.022311124	3.277274	0.031854582	1.750623659	0.032598702	0.297173287	0.006714838
AQP4	Hs00242342_m1	GCCTGGGAGTCACCATGGTTCATGG	282N	27.23326	0.032557936	1.388041	0.085487098	-0.138609341	0.08380824	1.100843467	0.063949588
AQP4	Hs00242342_m1	GCCTGGGAGTCACCATGGTTCATGG	282T	34.3377	0.277605598	6.983706	0.281756302	5.457055659	0.2818414	0.022764733	0.004447263
AQP4	Hs00242342_m1	GCCTGGGAGTCACCATGGTTCATGG	284N	27.775017	0.006535605	1.504487	0.01544425	-0.022163341	0.016925892	1.015481066	0.01191376
AQP4	Hs00242342_m1	GCCTGGGAGTCACCATGGTTCATGG	301T	32.876762	0.018826238	7.047422	0.020597325	5.520771659	0.021730411	0.021781216	0.000328077
AQP4	Hs00242342_m1	GCCTGGGAGTCACCATGGTTCATGG	30N	26.222733	0.017903054	1.288216	0.018442448	-0.238434341	0.019303489	1.179711705	0.01578473
AQP4	Hs00242342_m1	GCCTGGGAGTCACCATGGTTCATGG	30T	32.51741	0.114184295	7.762094	0.11437214	6.235443659	0.114581619	0.013272246	0.001054107
AQP4	Hs00242342_m1	GCCTGGGAGTCACCATGGTTCATGG	331N	28.375301	0.068940818	1.497507	0.07482547	-0.029143341	0.073432409	1.020406038	0.051938124
AQP4	Hs00242342_m1	GCCTGGGAGTCACCATGGTTCATGG	331T	27.400932	0.004447906	1.601108	0.011487006	0.074457659	0.013413139	0.949699067	0.008829617
AQP4	Hs00242342_m1	GCCTGGGAGTCACCATGGTTCATGG	334N	27.217848	0.1047833	1.266752	0.105456121	-0.259898341	0.103264005	1.197394328	0.085706078
AQP4	Hs00242342_m1	GCCTGGGAGTCACCATGGTTCATGG	334T	36.838512	0.327876063	9.932855	0.328009457	8.406204659	0.328082558	0.002947679	0.00067033
AQP4	Hs00242342_m1	GCCTGGGAGTCACCATGGTTCATGG	342N	30.95733	0.040889101	2.485612	0.044216745	0.958961659	0.044755798	0.514427025	0.015958738
AQP4	Hs00242342_m1	GCCTGGGAGTCACCATGGTTCATGG	342T	35.098446	0.119386798	7.984287	0.125462874	6.457636659	0.125653864	0.011377783	0.000990967
AQP4	Hs00242342_m1	GCCTGGGAGTCACCATGGTTCATGG	347T	39.27759	0.017981725	11.868025	0.077715806	10.34137466	0.078023762	0.00077079	4.17E-05
AQP4	Hs00242342_m1	GCCTGGGAGTCACCATGGTTCATGG	348N	26.734875	0.073675091	0.997162	0.086288378	-0.529488341	0.086565843	1.44341719	0.086609174
AQP4	Hs00242342_m1	GCCTGGGAGTCACCATGGTTCATGG	364T	30.830324	0.007332926	4.713217	0.020865623	3.186566659	0.021984885	0.109836795	0.001673777
AQP4	Hs00242342_m1	GCCTGGGAGTCACCATGGTTCATGG	368N	29.96939	0.024268919	1.98419	0.025757549	0.457539659	0.02610087	0.728227103	0.013174899
AQP4	Hs00242342_m1	GCCTGGGAGTCACCATGGTTCATGG	368T	34.9635	0.00749112	8.130834	0.008223503	6.604183659	0.010751138	0.010278805	7.66E-05

Gene	Assay_ID Applied Biosystems	Representative sequence	Sample_ID	Ct value	Ct value error	dCt value	dCt value error	ddCt value	ddCt value error	Expression level	Level Error
AQP4	Hs00242342_m1	GCCTGGGAGTCACCATGGTTCATGG	378N	28.301027	0.004110734	1.646555	0.011808707	0.119904659	0.013456154	0.920248463	0.008583245
AQP4	Hs00242342_m1	GCCTGGGAGTCACCATGGTTCATGG	378T	31.552042	0.016541085	4.823061	0.017166255	3.296410659	0.018510572	0.101784469	0.001305951
AQP4	Hs00242342_m1	GCCTGGGAGTCACCATGGTTCATGG	37N	27.489601	0.040557702	1.735266	0.046931125	0.208615659	0.046372152	0.865367198	0.027815261
AQP4	Hs00242342_m1	GCCTGGGAGTCACCATGGTTCATGG	37T	34.29058	0.264047104	9.09223	0.266701487	7.565579659	0.266791387	0.00527878	0.000976182
AQP4	Hs00242342_m1	GCCTGGGAGTCACCATGGTTCATGG	390N	27.702608	0.018405927	1.749029	0.075527241	0.222378659	0.074115018	0.857151034	0.044034091
AQP4	Hs00242342_m1	GCCTGGGAGTCACCATGGTTCATGG	390T	28.723026	0.005387255	2.569814	0.011153753	1.043163659	0.013128868	0.485262184	0.004416001
AQP4	Hs00242342_m1	GCCTGGGAGTCACCATGGTTCATGG	397T	34.35399	0.291784166	7.898729	0.295410541	6.372078659	0.295491707	0.012072944	0.002472771
AQP4	Hs00242342_m1	GCCTGGGAGTCACCATGGTTCATGG	400T	32.61252	0.046923566	6.501323	0.052490057	4.974672659	0.052944943	0.031803456	0.001167144
AQP4	Hs00242342_m1	GCCTGGGAGTCACCATGGTTCATGG	402T	27.925491	0.028043635	1.761274	0.04265293	0.234623659	0.043211496	0.849906677	0.025456342
AQP4	Hs00242342_m1	GCCTGGGAGTCACCATGGTTCATGG	40N	26.1286	0.012869138	1.370168	0.013306729	-0.156482341	0.014730289	1.114566225	0.011380009
AQP4	Hs00242342_m1	GCCTGGGAGTCACCATGGTTCATGG	40T	31.540892	0.014688946	4.968836	0.019360984	3.442185659	0.020562312	0.092002338	0.001311282
AQP4	Hs00242342_m1	GCCTGGGAGTCACCATGGTTCATGG	412T	30.875507	0.007028085	4.999956	0.009041094	3.473305659	0.011388694	0.090039031	0.000710772
AQP4	Hs00242342_m1	GCCTGGGAGTCACCATGGTTCATGG	421N	28.018988	0.008556331	1.321528	0.020597974	-0.205122341	0.021282674	1.152784099	0.0170059
AQP4	Hs00242342_m1	GCCTGGGAGTCACCATGGTTCATGG	421T	32.430992	0.105798282	5.952776	0.108387533	4.426125659	0.108608555	0.046516112	0.003501813
AQP4	Hs00242342_m1	GCCTGGGAGTCACCATGGTTCATGG	426N	26.015339	0.002308246	0.071837	0.016053185	-1.454813341	0.017145025	2.741210923	0.03257662
AQP4	Hs00242342_m1	GCCTGGGAGTCACCATGGTTCATGG	426T	37.4167	0	11.422605	0.00191969	9.895954659	0.007186527	0.001049593	5.23E-06
AQP4	Hs00242342_m1	GCCTGGGAGTCACCATGGTTCATGG	42N	27.282635	0.015149671	1.149903	0.022396723	-0.376747341	0.022951549	1.298411192	0.020656166
AQP4	Hs00242342_m1	GCCTGGGAGTCACCATGGTTCATGG	42T	26.973215	0.010882475	0.931581	0.057674636	-0.595069341	0.058088937	1.510545186	0.060820868
AQP4	Hs00242342_m1	GCCTGGGAGTCACCATGGTTCATGG	434N	28.3001	0.070232928	1.362272	0.105179744	-0.164378341	0.102994592	1.12068307	0.080006025
AQP4	Hs00242342_m1	GCCTGGGAGTCACCATGGTTCATGG	434T	NA	NA	NA	NA	NA	NA	NA	NA
AQP4	Hs00242342_m1	GCCTGGGAGTCACCATGGTTCATGG	445N	28.189108	0.008921216	1.475813	0.013257985	-0.050837341	0.014688274	1.035865967	0.010546292
AQP4	Hs00242342_m1	GCCTGGGAGTCACCATGGTTCATGG	445T	28.708595	0.021367733	1.882059	0.039198268	0.355408659	0.039805341	0.781648201	0.021566424
AQP4	Hs00242342_m1	GCCTGGGAGTCACCATGGTTCATGG	489T	32.616	0.131672812	6.407525	0.133062503	4.880874659	0.133242601	0.033939882	0.003134577
AQP4	Hs00242342_m1	GCCTGGGAGTCACCATGGTTCATGG	492N	27.338114	0.010426369	1.163788	0.043667083	-0.362862341	0.043221542	1.285974772	0.038526377
AQP4	Hs00242342_m1	GCCTGGGAGTCACCATGGTTCATGG	492T	32.523094	0.040967043	5.278887	0.051303268	3.752236659	0.051768584	0.074210305	0.002662907
AQP4	Hs00242342_m1	GCCTGGGAGTCACCATGGTTCATGG	49N	27.526693	0.053137009	2.118742	0.062596755	0.592091659	0.061548418	0.663380422	0.02830121
AQP4	Hs00242342_m1	GCCTGGGAGTCACCATGGTTCATGG	49T	28.965605	0.084875686	3.547728	0.084877863	2.021077659	0.085159924	0.246374072	0.014543058
AQP4	Hs00242342_m1	GCCTGGGAGTCACCATGGTTCATGG	51N	27.47801	0.041238975	1.382456	0.04335688	-0.144194341	0.042922414	1.105113341	0.032878835
AQP4	Hs00242342_m1	GCCTGGGAGTCACCATGGTTCATGG	51T	29.469355	0.022827275	3.09853	0.023614524	1.571879659	0.024609077	0.336369859	0.0057377
AQP4	Hs00242342_m1	GCCTGGGAGTCACCATGGTTCATGG	53N	27.0045	0.053556743	1.135544	0.0817807	-0.391106341	0.0802	1.311398674	0.072901182
AQP4	Hs00242342_m1	GCCTGGGAGTCACCATGGTTCATGG	53T	28.878378	0.022562849	2.947358	0.022578632	1.420707659	0.02361685	0.373529047	0.006114653
AQP4	Hs00242342_m1	GCCTGGGAGTCACCATGGTTCATGG	55N	28.055899	0.00165815	0.540863	0.006988621	-0.985787341	0.009725315	1.980393804	0.013349983
AQP4	Hs00242342_m1	GCCTGGGAGTCACCATGGTTCATGG	55T	34.84282	0.095080929	9.282239	0.095241567	7.755588659	0.095493021	0.00462738	0.00030629
AQP4	Hs00242342_m1	GCCTGGGAGTCACCATGGTTCATGG	65N	29.00371	0.014882935	1.824593	0.050489651	0.297942659	0.050962396	0.813411527	0.028733308
AQP4	Hs00242342_m1	GCCTGGGAGTCACCATGGTTCATGG	70T	31.2552	0.046193218	5.778163	0.051886752	4.251512659	0.052346881	0.05250095	0.001904949
AQP4	Hs00242342_m1	GCCTGGGAGTCACCATGGTTCATGG	75N	29.774368	0.010784903	2.293594	0.027639951	0.766943659	0.028494348	0.587661113	0.011606763
AQP4	Hs00242342_m1	GCCTGGGAGTCACCATGGTTCATGG	77N	27.594494	0.045300634	1.456997	0.045618548	-0.069653341	0.045104541	1.049464482	0.032810547
AQP4	Hs00242342_m1	GCCTGGGAGTCACCATGGTTCATGG	77T	34.978413	0.476854372	5.451409	0.478278531	3.924758659	0.478328667	0.065846079	0.02183141
AQP4	Hs00242342_m1	GCCTGGGAGTCACCATGGTTCATGG	84N	27.329597	0.034691823	0.892082	0.04874547	-0.634568341	0.04812563	1.552473175	0.051787625
AQP4	Hs00242342_m1	GCCTGGGAGTCACCATGGTTCATGG	84T	31.415209	0.03559018	5.606943	0.043176934	4.080292659	0.043728808	0.05911661	0.001791854
AQP4	Hs00242342_m1	GCCTGGGAGTCACCATGGTTCATGG	88N	27.51691	0.039533482	1.342466	0.071276244	-0.184184341	0.069981005	1.136174431	0.055112568
AQP4	Hs00242342_m1	GCCTGGGAGTCACCATGGTTCATGG	88T	28.65343	0.080971066	2.821393	0.123896659	1.294742659	0.12409006	0.407608867	0.035059529
AQP4	Hs00242342_m1	GCCTGGGAGTCACCATGGTTCATGG	89T	30.535559	0.049370376	3.421292	0.05032442	1.894641659	0.050798703	0.26894039	0.009469654
AQP4	Hs00242342_m1	GCCTGGGAGTCACCATGGTTCATGG	95N	28.784203	0.021213004	1.957177	0.022253642	0.430526659	0.022818312	0.74199087	0.01173566
AQP4	Hs00242342_m1	GCCTGGGAGTCACCATGGTTCATGG	95T	37.93532	0.007040209	11.377913	0.011986582	9.851262659	0.013843378	0.001082616	1.04E-05
AQP4	Hs00242342_m1	GCCTGGGAGTCACCATGGTTCATGG	97N	27.074854	0.004528158	1.136747	0.012063093	-0.389903341	0.013909679	1.310305612	0.012633252
AQP4	Hs00242342_m1	GCCTGGGAGTCACCATGGTTCATGG	NTC	NA	NA	NA	NA	NA	NA	NA	NA

Gene	Assay_ID Applied Biosystems	Representative sequence	Sample_ID	Ct value	Ct value error	dCt value	dCt value error	ddCt value	ddCt value error	Expression level	Level Error
AQP5	Hs00387048_m1	TCAACGCGCTCAACAACAACACAAC	10N	32.153057	0.055935426	6.766504	0.055992195	1.657427614	0.057552403	0.317003876	0.012646009
AQP5	Hs00387048_m1	TCAACGCGCTCAACAACAACACAAC	10T	27.371899	0.007500357	0.941731	0.007875173	-4.167345386	0.019536562	17.9678438	0.243315377
AQP5	Hs00387048_m1	TCAACGCGCTCAACAACAACACAAC	113N	32.192425	0.022235491	5.421235	0.078495313	0.312158614	0.07874709	0.805435734	0.043963359
AQP5	Hs00387048_m1	TCAACGCGCTCAACAACAACACAAC	113T	37.2260325	0.023969152	8.7847345	0.024800831	3.675658114	0.03057352	0.078255821	0.001658393
AQP5	Hs00387048_m1	TCAACGCGCTCAACAACAACACAAC	136N	35.736412	0.553689878	8.674189	0.554277553	3.565112614	0.541828899	0.084487832	0.031730856
AQP5	Hs00387048_m1	TCAACGCGCTCAACAACAACACAAC	136T	31.780155	0.066914896	5.628078	0.095707129	0.519001614	0.097362792	0.697854602	0.047095936
AQP5	Hs00387048_m1	TCAACGCGCTCAACAACAACACAAC	137N	31.463526	0.024714056	5.228119	0.025559492	0.119042614	0.030712402	0.920798499	0.019602156
AQP5	Hs00387048_m1	TCAACGCGCTCAACAACAACACAAC	137T	32.984882	0.077538719	4.922395	0.080686729	-0.186681386	0.082643858	1.138142647	0.06519777
AQP5	Hs00387048_m1	TCAACGCGCTCAACAACAACACAAC	147N	32.914165	0.104218652	7.057979	0.10430888	1.948902614	0.103467098	0.259013174	0.018575888
AQP5	Hs00387048_m1	TCAACGCGCTCAACAACAACACAAC	147T	33.748047	0.014617354	7.563122	0.021613127	2.454045614	0.028049708	0.182498231	0.003548236
AQP5	Hs00387048_m1	TCAACGCGCTCAACAACAACACAAC	148N	28.542145	0.022849792	2.347269	0.042806259	-2.761807386	0.045483457	6.782454134	0.2138286
AQP5	Hs00387048_m1	TCAACGCGCTCAACAACAACACAAC	148T	28.868269	0.010063793	3.116779	0.010930421	-1.992297386	0.0209555	3.97870073	0.057791607
AQP5	Hs00387048_m1	TCAACGCGCTCAACAACAACACAAC	154N	30.77262	0.042071514	4.906768	0.05230242	-0.202308386	0.055273882	1.150537803	0.04408048
AQP5	Hs00387048_m1	TCAACGCGCTCAACAACAACACAAC	156N	29.964981	0.035769159	3.882728	0.04830148	-1.226348386	0.05150429	2.339740267	0.083528852
AQP5	Hs00387048_m1	TCAACGCGCTCAACAACAACACAAC	161N	30.472462	0.092971291	4.368531	0.094266416	-0.740545386	0.093818441	1.67080734	0.108652581
AQP5	Hs00387048_m1	TCAACGCGCTCAACAACAACACAAC	161T	30.271406	0.082686374	3.867366	0.082693272	-1.241710386	0.084603996	2.364787234	0.138678268
AQP5	Hs00387048_m1	TCAACGCGCTCAACAACAACACAAC	165N	31.967188	0.030434442	6.128108	0.041997546	1.019031614	0.04475802	0.49344746	0.015308662
AQP5	Hs00387048_m1	TCAACGCGCTCAACAACAACACAAC	165T	36.365242	0.10303797	10.647638	0.103984671	5.538561614	0.105510524	0.02151428	0.001573432
AQP5	Hs00387048_m1	TCAACGCGCTCAACAACAACACAAC	169N	33.608627	0.000845818	6.467386	0.001618469	1.358309614	0.017948796	0.390039026	0.004852537
AQP5	Hs00387048_m1	TCAACGCGCTCAACAACAACACAAC	169T	34.243855	0.054615603	7.601838	0.057287738	2.492761614	0.060012864	0.17766586	0.0073905
AQP5	Hs00387048_m1	TCAACGCGCTCAACAACAACACAAC	16N	30.85568	0.031980586	5.520135	0.036858458	0.411058614	0.040205133	0.75207132	0.02095878
AQP5	Hs00387048_m1	TCAACGCGCTCAACAACAACACAAC	16T	34.239532	0.065331802	7.691672	0.070121635	2.582595614	0.072365064	0.166940325	0.008373667
AQP5	Hs00387048_m1	TCAACGCGCTCAACAACAACACAAC	188N	31.252579	0.074866741	4.103794	0.075043305	-1.005282386	0.075466412	2.007336365	0.105002422
AQP5	Hs00387048_m1	TCAACGCGCTCAACAACAACACAAC	188T	28.78338	0.001614271	2.449748	0.031286715	-2.659328386	0.036034947	6.317388895	0.15779272
AQP5	Hs00387048_m1	TCAACGCGCTCAACAACAACACAAC	202N	28.548588	0.075457948	1.463776	0.076003809	-3.645300386	0.07637844	12.51251928	0.662431541
AQP5	Hs00387048_m1	TCAACGCGCTCAACAACAACACAAC	202T	27.5747	0.054696432	1.019288	0.057897395	-4.089788386	0.060595109	17.02742516	0.715174488
AQP5	Hs00387048_m1	TCAACGCGCTCAACAACAACACAAC	210N	30.959574	0.009391179	4.751272	0.010384836	-0.357804386	0.020557278	1.281474159	0.018260006
AQP5	Hs00387048_m1	TCAACGCGCTCAACAACAACACAAC	210T	37.14032	0.047444744	10.287966	0.049865909	5.178889614	0.052974218	0.027605708	0.001013652
AQP5	Hs00387048_m1	TCAACGCGCTCAACAACAACACAAC	215N	33.683437	0.163736536	7.022892	0.163814539	1.913815614	0.161043722	0.265389719	0.029624659
AQP5	Hs00387048_m1	TCAACGCGCTCAACAACAACACAAC	215T	30.373232	0.010362283	4.790762	0.021340072	-0.318314386	0.027839856	1.246872878	0.024061053
AQP5	Hs00387048_m1	TCAACGCGCTCAACAACAACACAAC	22N	31.44063	0.028200674	5.453938	0.028319042	0.344861614	0.032941978	0.7873835	0.017978831
AQP5	Hs00387048_m1	TCAACGCGCTCAACAACAACACAAC	22T	31.8458	0.071576422	6.830103	0.071591443	1.721026614	0.0737902	0.303332794	0.015514705
AQP5	Hs00387048_m1	TCAACGCGCTCAACAACAACACAAC	233N	31.847757	0.076387482	5.624033	0.076500506	0.514956614	0.076850318	0.699813978	0.037278098
AQP5	Hs00387048_m1	TCAACGCGCTCAACAACAACACAAC	233T	NA	NA	NA	NA	NA	NA	NA	NA
AQP5	Hs00387048_m1	TCAACGCGCTCAACAACAACACAAC	248T	31.38978	0.014849449	5.76502	0.025417522	0.655943614	0.031075864	0.634660248	0.013670675
AQP5	Hs00387048_m1	TCAACGCGCTCAACAACAACACAAC	24N	29.92759	0.089937316	4.87785	0.103917902	-0.231226386	0.103090876	1.173832361	0.083878715
AQP5	Hs00387048_m1	TCAACGCGCTCAACAACAACACAAC	24T	34.100426	0.393131655	9.49178	0.398222843	4.382703614	0.398623997	0.04793743	0.013245356
AQP5	Hs00387048_m1	TCAACGCGCTCAACAACAACACAAC	25N	29.104189	0.027380259	4.083761	0.027427229	-1.025315386	0.032213638	2.035404294	0.045448119
AQP5	Hs00387048_m1	TCAACGCGCTCAACAACAACACAAC	25T	35.984386	0.560140612	10.898174	0.560253418	5.789097614	0.560538626	0.018084561	0.007026499
AQP5	Hs00387048_m1	TCAACGCGCTCAACAACAACACAAC	271N	32.126175	0.076963678	5.918935	0.077514625	0.809858614	0.077814277	0.570437759	0.030767557
AQP5	Hs00387048_m1	TCAACGCGCTCAACAACAACACAAC	271T	30.4554	0.020596971	1.783294	0.038711607	-3.325782386	0.042640913	10.02675163	0.296354966
AQP5	Hs00387048_m1	TCAACGCGCTCAACAACAACACAAC	273N	30.806477	0.143459418	4.26895	0.144598457	-0.840126386	0.14240076	1.790206965	0.176701816
AQP5	Hs00387048_m1	TCAACGCGCTCAACAACAACACAAC	273T	32.02934	0.009147538	6.340934	0.016578972	1.231857614	0.024382806	0.425768873	0.007195866
AQP5	Hs00387048_m1	TCAACGCGCTCAACAACAACACAAC	276N	31.346512	0.069017606	5.248421	0.069077926	0.139344614	0.069817767	0.907931516	0.043938427
AQP5	Hs00387048_m1	TCAACGCGCTCAACAACAACACAAC	276T	31.024345	0.035974118	6.760273	0.042556613	1.651196614	0.046159768	0.318375977	0.010186603
AQP5	Hs00387048_m1	TCAACGCGCTCAACAACAACACAAC	282N	31.931093	0.00417251	6.085874	0.079154398	0.976797614	0.079374436	0.508106345	0.027955079
AQP5	Hs00387048_m1	TCAACGCGCTCAACAACAACACAAC	282T	39.3174035	0.082673864	11.9634095	0.095690719	6.854333114	0.097346662	0.008642515	0.000583159
AQP5	Hs00387048_m1	TCAACGCGCTCAACAACAACACAAC	284N	30.147408	0.090656694	3.876878	0.091730294	-1.232198386	0.093456438	2.349246967	0.152182025
AQP5	Hs00387048_m1	TCAACGCGCTCAACAACAACACAAC	301T	32.95991	0.08024014	7.13057	0.080674052	2.021493614	0.082631481	0.246303048	0.014107199
AQP5	Hs00387048_m1	TCAACGCGCTCAACAACAACACAAC	30N	30.822426	0.005505612	5.887909	0.007065146	0.778832614	0.019165236	0.582838218	0.007742615
AQP5	Hs00387048_m1	TCAACGCGCTCAACAACAACACAAC	30T	30.121885	0.072728236	5.366569	0.073022802	0.257492614	0.075179708	0.836540554	0.043592632
AQP5	Hs00387048_m1	TCAACGCGCTCAACAACAACACAAC	331N	30.348717	0.044490034	3.470923	0.053154282	-1.638153386	0.054923677	3.112671626	0.118500007
AQP5	Hs00387048_m1	TCAACGCGCTCAACAACAACACAAC	331T	27.834711	0.036666938	2.034887	0.038165846	-3.074189386	0.042146064	8.422154763	0.246039989
AQP5	Hs00387048_m1	TCAACGCGCTCAACAACAACACAAC	334N	33.68752	0.149799301	7.736424	0.150270702	2.627347614	0.147900374	0.161841375	0.016591448
AQP5	Hs00387048_m1	TCAACGCGCTCAACAACAACACAAC	334T	36.628437	0.096496592	9.72278	0.096948868	4.613703614	0.09858368	0.040844804	0.002791048
AQP5	Hs00387048_m1	TCAACGCGCTCAACAACAACACAAC	342N	32.65493	0.052086809	4.183212	0.0547379	-0.925864386	0.057583822	1.899822174	0.075829624
AQP5	Hs00387048_m1	TCAACGCGCTCAACAACAACACAAC	342T	31.927288	0.0475523	4.813129	0.06122864	-0.295947386	0.063785619	1.22769091	0.05427968
AQP5	Hs00387048_m1	TCAACGCGCTCAACAACAACACAAC	347T	34.69612	0.012414185	7.286555	0.076619293	2.177478614	0.078677665	0.221061759	0.012055648
AQP5	Hs00387048_m1	TCAACGCGCTCAACAACAACACAAC	348N	31.717539	0.034001889	5.979826	0.056336433	0.870749614	0.059105436	0.546862631	0.022404287
AQP5	Hs00387048_m1	TCAACGCGCTCAACAACAACACAAC	364T	29.971663	0.063436361	3.854556	0.066376007	-1.254520386	0.068741786	2.385878186	0.113682742
AQP5	Hs00387048_m1	TCAACGCGCTCAACAACAACACAAC	368N	32.625187	0.090728285	4.639987	0.091137768	-0.469089386	0.090819612	1.384235478	0.087139503
AQP5	Hs00387048_m1	TCAACGCGCTCAACAACAACACAAC	368T	26.21294	0.040165681	-0.619726	0.040308697	-5.728802386	0.044095917	53.03240926	1.620933514

Gene	Assay_ID Applied Biosystems	Representative sequence	Sample_ID	Ct value	Ct value error	dCt value	dCt value error	ddCt value	ddCt value error	Expression level	Level Error
AQP5	Hs00387048_m1	TCAACGCGCTCAACAACAACACAAC	378N	29.441887	0.046217466	2.787415	0.047524748	-2.321661386	0.04975537	4.999075744	0.172407095
AQP5	Hs00387048_m1	TCAACGCGCTCAACAACAACACAAC	378T	28.993462	0.002400045	2.264481	0.00518006	-2.844595386	0.018614293	7.183044167	0.092678831
AQP5	Hs00387048_m1	TCAACGCGCTCAACAACAACACAAC	37N	29.249084	0.002929475	3.494749	0.023794646	-1.614327386	0.029327604	3.06168824	0.062239058
AQP5	Hs00387048_m1	TCAACGCGCTCAACAACAACACAAC	37T	32.906113	0.105168393	7.707763	0.111665576	2.598686614	0.113087841	0.165088712	0.012940729
AQP5	Hs00387048_m1	TCAACGCGCTCAACAACAACACAAC	390N	32.16317	0.031557966	6.209591	0.079758957	1.100514614	0.079949931	0.466350118	0.025843757
AQP5	Hs00387048_m1	TCAACGCGCTCAACAACAACACAAC	390T	35.001266	0.00892468	8.848054	0.013230027	3.738977614	0.022241684	0.074895476	0.001154646
AQP5	Hs00387048_m1	TCAACGCGCTCAACAACAACACAAC	397T	27.548853	0.027216292	1.093592	0.053573454	-4.015484386	0.056478084	16.17265222	0.633120914
AQP5	Hs00387048_m1	TCAACGCGCTCAACAACAACACAAC	400T	26.845652	0.023909229	0.734455	0.03354156	-4.374621386	0.038009146	20.74398821	0.546519705
AQP5	Hs00387048_m1	TCAACGCGCTCAACAACAACACAAC	402T	35.86537	0.336633889	9.701153	0.338164461	4.592076614	0.338636769	0.041461709	0.009732105
AQP5	Hs00387048_m1	TCAACGCGCTCAACAACAACACAAC	40N	28.567123	0.022999325	3.808691	0.023247007	-1.300385386	0.02890532	2.462946664	0.049346715
AQP5	Hs00387048_m1	TCAACGCGCTCAACAACAACACAAC	40T	33.601665	0.062578996	7.029609	0.063837397	1.920532614	0.066293832	0.264156971	0.012138378
AQP5	Hs00387048_m1	TCAACGCGCTCAACAACAACACAAC	412T	36.80951	0.425997896	10.933959	0.426035861	5.824882614	0.426410851	0.017641504	0.00521422
AQP5	Hs00387048_m1	TCAACGCGCTCAACAACAACACAAC	421N	30.48426	0.065656273	3.7868	0.068277462	-1.322276386	0.069062075	2.500603616	0.119704352
AQP5	Hs00387048_m1	TCAACGCGCTCAACAACAACACAAC	421T	37.105297	0.212352893	10.627081	0.213654703	5.518004614	0.214401471	0.021823032	0.00324316
AQP5	Hs00387048_m1	TCAACGCGCTCAACAACAACACAAC	426N	33.19401	0.034447026	7.250508	0.037933816	2.141431614	0.04114882	0.226654763	0.00646469
AQP5	Hs00387048_m1	TCAACGCGCTCAACAACAACACAAC	426T	28.924755	0.010414244	2.93066	0.010589697	-2.178416386	0.020779812	4.526564106	0.065198221
AQP5	Hs00387048_m1	TCAACGCGCTCAACAACAACACAAC	42N	31.450653	0.00696169	5.317921	0.017904351	0.208844614	0.025013067	0.865229876	0.015001128
AQP5	Hs00387048_m1	TCAACGCGCTCAACAACAACACAAC	42T	35.8211	0.359192696	9.779466	0.363630759	4.670389614	0.364070031	0.039271061	0.009910214
AQP5	Hs00387048_m1	TCAACGCGCTCAACAACAACACAAC	434N	30.458836	0.004764294	3.521008	0.078439868	-1.588068386	0.078694336	3.006465465	0.163992942
AQP5	Hs00387048_m1	TCAACGCGCTCAACAACAACACAAC	434T	37.20752	0	9.977656	0.063231401	4.868579614	0.065710494	0.034230363	0.001559092
AQP5	Hs00387048_m1	TCAACGCGCTCAACAACAACACAAC	445N	30.217226	0.005278136	3.503931	0.011137539	-1.605145386	0.020930009	3.042264073	0.044135879
AQP5	Hs00387048_m1	TCAACGCGCTCAACAACAACACAAC	445T	33.110092	0.110259468	6.283556	0.115052485	1.174479614	0.116433386	0.443043538	0.035756038
AQP5	Hs00387048_m1	TCAACGCGCTCAACAACAACACAAC	489T	35.1042885	0.201941565	8.8958135	0.202850428	3.786737114	0.203636821	0.072456699	0.010227284
AQP5	Hs00387048_m1	TCAACGCGCTCAACAACAACACAAC	492N	32.244377	0.17948492	6.070051	0.184425978	0.960974614	0.181070587	0.513709759	0.064474976
AQP5	Hs00387048_m1	TCAACGCGCTCAACAACAACACAAC	492T	33.16329	0.138769024	5.919083	0.14216388	0.810006614	0.143283731	0.570379243	0.056648192
AQP5	Hs00387048_m1	TCAACGCGCTCAACAACAACACAAC	49N	31.472784	0.002624057	6.064833	0.033191832	0.955756614	0.03703079	0.51557113	0.01323357
AQP5	Hs00387048_m1	TCAACGCGCTCAACAACAACACAAC	49T	35.71629	0.020833107	10.298413	0.020841976	5.189336614	0.027459913	0.027406528	0.000521649
AQP5	Hs00387048_m1	TCAACGCGCTCAACAACAACACAAC	51N	31.313587	0.164384901	5.218033	0.164928959	0.108956614	0.162125833	0.927258432	0.10420258
AQP5	Hs00387048_m1	TCAACGCGCTCAACAACAACACAAC	51T	32.681454	0.059143762	6.310629	0.059452046	1.201552614	0.062082241	0.434807095	0.018710676
AQP5	Hs00387048_m1	TCAACGCGCTCAACAACAACACAAC	53N	30.211147	0.042317465	4.342191	0.074903444	-0.766885386	0.075333664	1.701592272	0.088852583
AQP5	Hs00387048_m1	TCAACGCGCTCAACAACAACACAAC	53T	25.660477	0.002732599	-0.270543	0.002859996	-5.379619386	0.01810631	41.63195453	0.522495096
AQP5	Hs00387048_m1	TCAACGCGCTCAACAACAACACAAC	55N	31.815298	0.05837935	4.300262	0.058772782	-0.808814386	0.060140567	1.751771237	0.073024799
AQP5	Hs00387048_m1	TCAACGCGCTCAACAACAACACAAC	55T	30.383722	0.039545029	4.823141	0.039929717	-0.285935386	0.043749757	1.219200489	0.03697228
AQP5	Hs00387048_m1	TCAACGCGCTCAACAACAACACAAC	65N	32.864235	0.018824506	5.685118	0.051788658	0.576041614	0.054787991	0.670801763	0.025474463
AQP5	Hs00387048_m1	TCAACGCGCTCAACAACAACACAAC	70T	32.342243	0.017782388	6.865206	0.029574228	1.756129614	0.034558557	0.296041305	0.007091423
AQP5	Hs00387048_m1	TCAACGCGCTCAACAACAACACAAC	75N	32.734325	0.280629759	5.253551	0.281781323	0.144474614	0.282347964	0.904708785	0.177059376
AQP5	Hs00387048_m1	TCAACGCGCTCAACAACAACACAAC	77N	30.815754	0.037328582	4.678257	0.037713757	-0.430819386	0.040955282	1.347998963	0.038267046
AQP5	Hs00387048_m1	TCAACGCGCTCAACAACAACACAAC	77T	37.575535	0	8.048531	0.036881713	2.939454614	0.040986822	0.13035749	0.003703443
AQP5	Hs00387048_m1	TCAACGCGCTCAACAACAACACAAC	84N	30.334814	0.08002017	3.897299	0.08703922	-1.211777386	0.086897232	2.316228182	0.13951238
AQP5	Hs00387048_m1	TCAACGCGCTCAACAACAACACAAC	84T	NA	NA	NA	NA	NA	NA	NA	NA
AQP5	Hs00387048_m1	TCAACGCGCTCAACAACAACACAAC	88N	30.704294	0.022629821	4.52985	0.063478465	-0.579226386	0.064544672	1.494047883	0.066842144
AQP5	Hs00387048_m1	TCAACGCGCTCAACAACAACACAAC	88T	29.469368	0.064847982	3.637331	0.114014601	-1.471745386	0.115407921	2.773572402	0.221871023
AQP5	Hs00387048_m1	TCAACGCGCTCAACAACAACACAAC	89T	35.241596	0.143410343	8.127329	0.143741572	3.018252614	0.144849227	0.123428494	0.012392447
AQP5	Hs00387048_m1	TCAACGCGCTCAACAACAACACAAC	95N	34.226803	0.204474371	7.399777	0.20458495	2.290700614	0.200679248	0.20437624	0.028428787
AQP5	Hs00387048_m1	TCAACGCGCTCAACAACAACACAAC	95T	30.018232	0.024836454	3.460825	0.02666389	-1.648251386	0.032103301	3.134534886	0.06975065
AQP5	Hs00387048_m1	TCAACGCGCTCAACAACAACACAAC	97N	32.046844	0.003504516	6.108737	0.011717321	0.999660614	0.021376494	0.500117636	0.007410271
AQP5	Hs00387048_m1	TCAACGCGCTCAACAACAACACAAC	NTC	NA	NA	NA	NA	NA	NA	NA	NA

Gene	Assay_ID Applied Biosystems	Representative sequence	Sample_ID	Ct value	Ct value error	dCt value	dCt value error	ddCt value	ddCt value error	Expression level	Level Error
AQP9	Hs00175573_m1	ATCTTGATTGTCCTTGGATGTGGCT	10N	28.658203	0.002071533	3.27165	0.003262703	-1.512001409	0.010049004	2.85205422	0.019865809
AQP9	Hs00175573_m1	ATCTTGATTGTCCTTGGATGTGGCT	10T	31.226519	0.029185633	4.796351	0.029284197	0.012699591	0.030795865	0.991235945	0.021158989
AQP9	Hs00175573_m1	ATCTTGATTGTCCTTGGATGTGGCT	113N	32.35105	0.021910443	5.57986	0.078403856	0.796208591	0.077191769	0.575860555	0.030811567
AQP9	Hs00175573_m1	ATCTTGATTGTCCTTGGATGTGGCT	113T	31.735687	0.042798398	3.294389	0.043269665	-1.489262409	0.044306715	2.807454048	0.086219931
AQP9	Hs00175573_m1	ATCTTGATTGTCCTTGGATGTGGCT	136N	32.76584	0.135176751	5.703617	0.13756409	0.919965591	0.134738724	0.528521626	0.049360625
AQP9	Hs00175573_m1	ATCTTGATTGTCCTTGGATGTGGCT	136T	32.91601	0.041294401	6.763933	0.079921703	1.980281591	0.080487886	0.253440398	0.014139427
AQP9	Hs00175573_m1	ATCTTGATTGTCCTTGGATGTGGCT	137N	31.638868	0.006902222	5.403461	0.009494407	0.619809591	0.013299152	0.65075681	0.005998852
AQP9	Hs00175573_m1	ATCTTGATTGTCCTTGGATGTGGCT	137T	31.436037	0.024020658	3.37355	0.032788526	-1.410101409	0.034145403	2.657558425	0.062898535
AQP9	Hs00175573_m1	ATCTTGATTGTCCTTGGATGTGGCT	147N	32.932556	0.16223889	7.07637	0.162296865	2.292718591	0.158851529	0.204090567	0.0224719
AQP9	Hs00175573_m1	ATCTTGATTGTCCTTGGATGTGGCT	147T	31.879848	0.103862427	5.694923	0.105075515	0.911271591	0.105506801	0.53171623	0.038885334
AQP9	Hs00175573_m1	ATCTTGATTGTCCTTGGATGTGGCT	148N	32.00041	0.005675353	5.805534	0.036639766	1.021882591	0.037044191	0.492473298	0.012645275
AQP9	Hs00175573_m1	ATCTTGATTGTCCTTGGATGTGGCT	148T	30.618237	0.025373967	4.866747	0.02572999	0.083095591	0.027438176	0.944029867	0.017954216
AQP9	Hs00175573_m1	ATCTTGATTGTCCTTGGATGTGGCT	154N	29.205465	0.0099183	3.339613	0.032617533	-1.444038409	0.033981239	2.72081414	0.064086056
AQP9	Hs00175573_m1	ATCTTGATTGTCCTTGGATGTGGCT	156N	29.309631	0.021897164	3.227378	0.039154643	-1.556273409	0.040297732	2.940931964	0.082146874
AQP9	Hs00175573_m1	ATCTTGATTGTCCTTGGATGTGGCT	161N	30.546108	0.065808116	4.442177	0.067625471	-0.341474409	0.06675442	1.267050836	0.05862725
AQP9	Hs00175573_m1	ATCTTGATTGTCCTTGGATGTGGCT	161T	28.894125	0.013251921	2.490085	0.013294895	-2.293566409	0.016357731	4.90266576	0.055587969
AQP9	Hs00175573_m1	ATCTTGATTGTCCTTGGATGTGGCT	165N	29.257923	0.031739831	3.418843	0.042952945	-1.364808409	0.043033882	2.575421219	0.076821761
AQP9	Hs00175573_m1	ATCTTGATTGTCCTTGGATGTGGCT	165T	29.441841	0.03202331	3.724237	0.034949691	-1.059414409	0.036225709	2.084085418	0.05233086
AQP9	Hs00175573_m1	ATCTTGATTGTCCTTGGATGTGGCT	169N	30.68019	0.009151579	3.538949	0.009255022	-1.244702409	0.01313709	2.369696684	0.021578308
AQP9	Hs00175573_m1	ATCTTGATTGTCCTTGGATGTGGCT	169T	31.098543	0.072013476	4.456526	0.074060527	-0.327125409	0.074671164	1.254511251	0.064931127
AQP9	Hs00175573_m1	ATCTTGATTGTCCTTGGATGTGGCT	16N	30.61514	0.008925835	5.279595	0.020382801	0.495943591	0.022077029	0.709097743	0.010851061
AQP9	Hs00175573_m1	ATCTTGATTGTCCTTGGATGTGGCT	16T	30.96854	0.13191934	4.42068	0.134355914	-0.362971409	0.134693477	1.286071996	0.120070773
AQP9	Hs00175573_m1	ATCTTGATTGTCCTTGGATGTGGCT	188N	31.620504	0.057501777	4.471719	0.057731474	-0.311932409	0.057203563	1.241369331	0.049220901
AQP9	Hs00175573_m1	ATCTTGATTGTCCTTGGATGTGGCT	188T	29.884892	0.012566029	3.55126	0.033677258	-1.232391409	0.034999698	2.349561302	0.05700022
AQP9	Hs00175573_m1	ATCTTGATTGTCCTTGGATGTGGCT	202N	32.41037	0.021269584	5.325558	0.023131628	0.541906591	0.024526962	0.686862587	0.01167721
AQP9	Hs00175573_m1	ATCTTGATTGTCCTTGGATGTGGCT	202T	31.971937	0.007905657	5.416525	0.020564728	0.632873591	0.022665595	0.644890628	0.010131614
AQP9	Hs00175573_m1	ATCTTGATTGTCCTTGGATGTGGCT	210N	30.373867	0.021967023	4.165565	0.022409834	-0.618086409	0.023878745	1.534838021	0.025403849
AQP9	Hs00175573_m1	ATCTTGATTGTCCTTGGATGTGGCT	210T	31.08461	0.079856202	4.232256	0.081318007	-0.551395409	0.081874534	1.46550248	0.083168881
AQP9	Hs00175573_m1	ATCTTGATTGTCCTTGGATGTGGCT	215N	31.661911	0.033245561	5.001366	0.033627627	0.217714591	0.034208736	0.859926589	0.020390312
AQP9	Hs00175573_m1	ATCTTGATTGTCCTTGGATGTGGCT	215T	27.860588	0.000379896	2.278118	0.01865921	-2.505533409	0.020952022	5.678592588	0.082469263
AQP9	Hs00175573_m1	ATCTTGATTGTCCTTGGATGTGGCT	22N	30.642536	0.026338719	4.655844	0.026465416	-0.127807409	0.027557253	1.09263187	0.020870615
AQP9	Hs00175573_m1	ATCTTGATTGTCCTTGGATGTGGCT	22T	31.32106	0.010998523	6.305363	0.011095857	1.521711591	0.014626659	0.348272486	0.003530935
AQP9	Hs00175573_m1	ATCTTGATTGTCCTTGGATGTGGCT	233N	30.83072	0.078825055	4.606996	0.078934589	-0.176655409	0.077706359	1.13026057	0.060878031
AQP9	Hs00175573_m1	ATCTTGATTGTCCTTGGATGTGGCT	233T	29.629366	0.072895668	3.589533	0.108917774	-1.194118409	0.109333904	2.28804973	0.173398676
AQP9	Hs00175573_m1	ATCTTGATTGTCCTTGGATGTGGCT	248T	29.642397	0.027199549	4.017637	0.034137366	-0.766014409	0.035442642	1.700565305	0.041777732
AQP9	Hs00175573_m1	ATCTTGATTGTCCTTGGATGTGGCT	24N	27.947992	0.001288646	2.898252	0.05207562	-1.885399409	0.051763158	3.694551935	0.132558628
AQP9	Hs00175573_m1	ATCTTGATTGTCCTTGGATGTGGCT	24T	29.767485	0.034257078	5.158839	0.072128233	0.375187591	0.07275509	0.771005154	0.038881779
AQP9	Hs00175573_m1	ATCTTGATTGTCCTTGGATGTGGCT	25N	29.996878	0.03783665	4.97645	0.037870653	0.192798591	0.038207554	0.874906899	0.02317056
AQP9	Hs00175573_m1	ATCTTGATTGTCCTTGGATGTGGCT	25T	31.932726	0.013238642	6.846514	0.017368014	2.062862591	0.019810831	0.239340659	0.003286583
AQP9	Hs00175573_m1	ATCTTGATTGTCCTTGGATGTGGCT	271N	32.423893	0.07379287	6.216653	0.074367312	1.433001591	0.07327982	0.370359541	0.018811932
AQP9	Hs00175573_m1	ATCTTGATTGTCCTTGGATGTGGCT	271T	34.577557	0.080494175	5.905451	0.086911826	1.121799591	0.087432755	0.459520271	0.02784866
AQP9	Hs00175573_m1	ATCTTGATTGTCCTTGGATGTGGCT	273N	32.039944	0.134010503	5.502417	0.135229154	0.718765591	0.132463284	0.607617114	0.055789308
AQP9	Hs00175573_m1	ATCTTGATTGTCCTTGGATGTGGCT	273T	32.293682	0.100437008	6.605276	0.101384305	1.821624591	0.101831225	0.282902221	0.019968378
AQP9	Hs00175573_m1	ATCTTGATTGTCCTTGGATGTGGCT	276N	31.074991	0.015064223	4.9769	0.015338214	0.193248591	0.017759174	0.874634044	0.010766502
AQP9	Hs00175573_m1	ATCTTGATTGTCCTTGGATGTGGCT	276T	29.920906	0.00325741	5.656834	0.022968214	0.873182591	0.024866845	0.545941173	0.009410051
AQP9	Hs00175573_m1	ATCTTGATTGTCCTTGGATGTGGCT	282N	31.180384	0.06888597	5.335165	0.104848947	0.551513591	0.102880646	0.68230392	0.048656068
AQP9	Hs00175573_m1	ATCTTGATTGTCCTTGGATGTGGCT	282T	31.944645	0.043074949	4.590651	0.06463124	-0.193000409	0.065330072	1.14313866	0.051765154
AQP9	Hs00175573_m1	ATCTTGATTGTCCTTGGATGTGGCT	284N	30.944847	0.013953401	4.674317	0.019761279	-0.109334409	0.021939217	1.078730447	0.016404369
AQP9	Hs00175573_m1	ATCTTGATTGTCCTTGGATGTGGCT	301T	31.419983	0.038818723	5.590643	0.039707881	0.806991591	0.040835486	0.571572499	0.016178361
AQP9	Hs00175573_m1	ATCTTGATTGTCCTTGGATGTGGCT	30N	30.356758	0.041323846	5.422241	0.041560375	0.638589591	0.041708198	0.642340608	0.018570016
AQP9	Hs00175573_m1	ATCTTGATTGTCCTTGGATGTGGCT	30T	28.679178	0.015510515	3.923862	0.016837736	-0.859789409	0.019347622	1.814773388	0.024337471
AQP9	Hs00175573_m1	ATCTTGATTGTCCTTGGATGTGGCT	331N	32.87142	0.020360257	5.993626	0.03550429	1.209974591	0.035973293	0.432276229	0.010778716
AQP9	Hs00175573_m1	ATCTTGATTGTCCTTGGATGTGGCT	331T	31.287954	0.041647739	5.48813	0.042973266	0.704478591	0.0440173	0.613664235	0.018723183
AQP9	Hs00175573_m1	ATCTTGATTGTCCTTGGATGTGGCT	334N	31.795155	0.022381561	5.844059	0.025345366	1.060407591	0.026533173	0.479496573	0.00881861
AQP9	Hs00175573_m1	ATCTTGATTGTCCTTGGATGTGGCT	334T	32.364403	0.034073481	5.458746	0.035334019	0.675094591	0.036596639	0.626291155	0.015887038
AQP9	Hs00175573_m1	ATCTTGATTGTCCTTGGATGTGGCT	342N	34.00061	0.038047383	5.528892	0.041602948	0.745240591	0.042680516	0.596568377	0.017648807
AQP9	Hs00175573_m1	ATCTTGATTGTCCTTGGATGTGGCT	342T	35.03743	0.115354584	7.923271	0.121632253	3.139619591	0.122005025	0.11346981	0.009595851
AQP9	Hs00175573_m1	ATCTTGATTGTCCTTGGATGTGGCT	347T	33.71848	0.220617085	6.308915	0.233212997	1.525263591	0.233407633	0.347416074	0.056207002
AQP9	Hs00175573_m1	ATCTTGATTGTCCTTGGATGTGGCT	348N	31.96863	0.031995597	6.230917	0.055148739	1.447265591	0.055966102	0.36671582	0.014225914
AQP9	Hs00175573_m1	ATCTTGATTGTCCTTGGATGTGGCT	364T	30.10074	0.046836963	3.983633	0.050747449	-0.800018409	0.05163453	1.741123344	0.062315377
AQP9	Hs00175573_m1	ATCTTGATTGTCCTTGGATGTGGCT	368N	34.02602	0.105887771	6.04082	0.10623884	1.257168591	0.10423282	0.418364229	0.030226266
AQP9	Hs00175573_m1	ATCTTGATTGTCCTTGGATGTGGCT	368T	34.758926	0.08488781	7.92626	0.084955573	3.142608591	0.085488423	0.113234965	0.006709858

Gene	Assay_ID Applied Biosystems	Representative sequence	Sample_ID	Ct value	Ct value error	dCt value	dCt value error	ddCt value	ddCt value error	Expression level	Level Error
AQP9	Hs00175573_m1	ATCTTGATTGTCCTTGGATGTGGCT	378N	30.946999	0.01094252	4.292527	0.015565544	-0.491124409	0.017946985	1.4055399	0.017484779
AQP9	Hs00175573_m1	ATCTTGATTGTCCTTGGATGTGGCT	378T	35.130966	0.085130297	8.401985	0.085253975	3.618333591	0.085784972	0.081427866	0.004841832
AQP9	Hs00175573_m1	ATCTTGATTGTCCTTGGATGTGGCT	37N	30.573654	0.004502755	4.819319	0.024039096	0.035667591	0.025346244	0.975580218	0.017139654
AQP9	Hs00175573_m1	ATCTTGATTGTCCTTGGATGTGGCT	37T	28.397326	0.034507648	3.198976	0.050986153	-1.584675409	0.051869152	2.999403069	0.107837408
AQP9	Hs00175573_m1	ATCTTGATTGTCCTTGGATGTGGCT	390N	30.85912	0.032071807	4.905541	0.07996366	0.121889591	0.078704277	0.91898321	0.050133886
AQP9	Hs00175573_m1	ATCTTGATTGTCCTTGGATGTGGCT	390T	28.94202	0.00587223	2.788808	0.01139591	-1.994843409	0.014855568	3.985728415	0.041041423
AQP9	Hs00175573_m1	ATCTTGATTGTCCTTGGATGTGGCT	397T	30.757442	0.030088609	4.302181	0.055088228	-0.481470409	0.055906476	1.396165928	0.054103407
AQP9	Hs00175573_m1	ATCTTGATTGTCCTTGGATGTGGCT	400T	29.64391	0.018725201	3.532713	0.030066895	-1.250938409	0.031541073	2.379961786	0.052032167
AQP9	Hs00175573_m1	ATCTTGATTGTCCTTGGATGTGGCT	402T	30.129457	0.047312123	3.96524	0.057194964	-0.818411409	0.057983489	1.763463126	0.070875509
AQP9	Hs00175573_m1	ATCTTGATTGTCCTTGGATGTGGCT	40N	28.603718	0.000156462	3.845286	0.003388042	-0.938365409	0.010088518	1.916355754	0.013400745
AQP9	Hs00175573_m1	ATCTTGATTGTCCTTGGATGTGGCT	40T	32.58782	0.006778092	6.015764	0.014318698	1.232112591	0.017200181	0.425693631	0.005075229
AQP9	Hs00175573_m1	ATCTTGATTGTCCTTGGATGTGGCT	412T	30.060505	0.004585316	4.184954	0.00730565	-0.598697409	0.012008066	1.514348663	0.012604464
AQP9	Hs00175573_m1	ATCTTGATTGTCCTTGGATGTGGCT	421N	32.280434	0.083189823	5.582974	0.08527375	0.799322591	0.083856459	0.574618923	0.033399649
AQP9	Hs00175573_m1	ATCTTGATTGTCCTTGGATGTGGCT	421T	30.605629	0.079423767	4.127413	0.082841509	-0.656238409	0.083387869	1.575968183	0.091091066
AQP9	Hs00175573_m1	ATCTTGATTGTCCTTGGATGTGGCT	426N	29.509262	0.071877799	3.56576	0.073612463	-1.217891409	0.072548653	2.326064995	0.116970584
AQP9	Hs00175573_m1	ATCTTGATTGTCCTTGGATGTGGCT	426T	30.686277	0.07386042	4.692182	0.073885363	-0.091469409	0.074497436	1.065454814	0.055017623
AQP9	Hs00175573_m1	ATCTTGATTGTCCTTGGATGTGGCT	42N	29.313862	0.038532357	3.18113	0.041914714	-1.602521409	0.042045307	3.036735824	0.088501371
AQP9	Hs00175573_m1	ATCTTGATTGTCCTTGGATGTGGCT	42T	30.040646	0.042248183	3.999012	0.070660062	-0.784639409	0.071299828	1.722661699	0.085136137
AQP9	Hs00175573_m1	ATCTTGATTGTCCTTGGATGTGGCT	434N	32.275433	0.02201783	5.337605	0.081332031	0.553953591	0.080031514	0.681150928	0.037785906
AQP9	Hs00175573_m1	ATCTTGATTGTCCTTGGATGTGGCT	434T	32.794674	0.009910795	5.56481	0.06400339	0.781158591	0.064709003	0.581899297	0.02609985
AQP9	Hs00175573_m1	ATCTTGATTGTCCTTGGATGTGGCT	445N	31.168589	0.043175408	4.455294	0.044275297	-0.328357409	0.044294677	1.255583007	0.038549826
AQP9	Hs00175573_m1	ATCTTGATTGTCCTTGGATGTGGCT	445T	31.957218	0.002349816	5.130682	0.032946105	0.347030591	0.034296748	0.786200621	0.018690107
AQP9	Hs00175573_m1	ATCTTGATTGTCCTTGGATGTGGCT	489T	30.641869	0.023225069	4.433394	0.030121492	-0.350257409	0.031593123	1.274788058	0.027916181
AQP9	Hs00175573_m1	ATCTTGATTGTCCTTGGATGTGGCT	492N	30.552237	0.003235471	4.377911	0.042527323	-0.405740409	0.04262847	1.324768624	0.039144004
AQP9	Hs00175573_m1	ATCTTGATTGTCCTTGGATGTGGCT	492T	31.38311	0.023348622	4.138903	0.038715434	-0.644748409	0.039871117	1.56346661	0.043208827
AQP9	Hs00175573_m1	ATCTTGATTGTCCTTGGATGTGGCT	49N	29.7056	0.036870166	4.297649	0.049540096	-0.486002409	0.049330384	1.40055866	0.047889605
AQP9	Hs00175573_m1	ATCTTGATTGTCCTTGGATGTGGCT	49T	29.0042	0.004124013	3.586323	0.004168583	-1.197328409	0.010401837	2.293146314	0.016533594
AQP9	Hs00175573_m1	ATCTTGATTGTCCTTGGATGTGGCT	51N	31.757023	0.013392794	5.661469	0.018934965	0.877817591	0.020810023	0.544190022	0.007849619
AQP9	Hs00175573_m1	ATCTTGATTGTCCTTGGATGTGGCT	51T	30.938898	0.010214481	4.568073	0.011869998	-0.215578409	0.015222286	1.16116936	0.012251828
AQP9	Hs00175573_m1	ATCTTGATTGTCCTTGGATGTGGCT	53N	30.715279	0.012572957	4.846323	0.063070099	0.062671591	0.062352608	0.957489392	0.041382246
AQP9	Hs00175573_m1	ATCTTGATTGTCCTTGGATGTGGCT	53T	28.454994	0.045356637	2.523974	0.045364491	-2.259677409	0.046354699	4.788843899	0.153868567
AQP9	Hs00175573_m1	ATCTTGATTGTCCTTGGATGTGGCT	55N	32.379818	0.004589935	4.864782	0.008195051	0.081130591	0.01244698	0.945316544	0.008155803
AQP9	Hs00175573_m1	ATCTTGATTGTCCTTGGATGTGGCT	55T	30.778982	0.004364191	5.218401	0.007044085	0.434749591	0.011850749	0.739822155	0.006077131
AQP9	Hs00175573_m1	ATCTTGATTGTCCTTGGATGTGGCT	65N	32.858788	0.002110215	5.679671	0.048292402	0.896019591	0.049223746	0.537367289	0.018334596
AQP9	Hs00175573_m1	ATCTTGATTGTCCTTGGATGTGGCT	70T	31.095818	0.015022654	5.618781	0.028001817	0.835129591	0.029579095	0.560532687	0.011492415
AQP9	Hs00175573_m1	ATCTTGATTGTCCTTGGATGTGGCT	75N	32.11779	0.089421164	4.637016	0.092972025	-0.146635409	0.093459181	1.106984797	0.071711547
AQP9	Hs00175573_m1	ATCTTGATTGTCCTTGGATGTGGCT	77N	29.872936	0.027881399	3.735439	0.028395015	-1.048212409	0.029333412	2.067965918	0.042046652
AQP9	Hs00175573_m1	ATCTTGATTGTCCTTGGATGTGGCT	77T	32.78689	0.017358613	3.259886	0.040762509	-1.523765409	0.041861716	2.875405469	0.083433716
AQP9	Hs00175573_m1	ATCTTGATTGTCCTTGGATGTGGCT	84N	30.845545	0.015682565	4.40803	0.037663525	-0.375621409	0.038011618	1.29739826	0.03418339
AQP9	Hs00175573_m1	ATCTTGATTGTCCTTGGATGTGGCT	84T	31.139772	0.005177677	5.331506	0.024987899	0.547854591	0.026743527	0.684036592	0.012680123
AQP9	Hs00175573_m1	ATCTTGATTGTCCTTGGATGTGGCT	88N	30.771372	0.061793222	4.596928	0.085649337	-0.186723409	0.084221044	1.138175799	0.066443948
AQP9	Hs00175573_m1	ATCTTGATTGTCCTTGGATGTGGCT	88T	31.094984	0.042316311	5.262947	0.102882159	0.479295591	0.1033226	0.71732778	0.051373415
AQP9	Hs00175573_m1	ATCTTGATTGTCCTTGGATGTGGCT	89T	33.192905	0.098617199	6.078638	0.099098261	1.294986591	0.099555444	0.407539954	0.028122936
AQP9	Hs00175573_m1	ATCTTGATTGTCCTTGGATGTGGCT	95N	29.580034	0.03153314	2.753008	0.032242394	-2.030643409	0.03291109	4.085870302	0.093207809
AQP9	Hs00175573_m1	ATCTTGATTGTCCTTGGATGTGGCT	95T	32.756786	0.059636819	6.199379	0.060420723	1.415727591	0.061167678	0.374820667	0.015891723
AQP9	Hs00175573_m1	ATCTTGATTGTCCTTGGATGTGGCT	97N	29.907944	0.103891872	3.969837	0.104491794	-0.813814409	0.104925479	1.757852974	0.127846537
AQP9	Hs00175573_m1	ATCTTGATTGTCCTTGGATGTGGCT	NTC	NA	NA	NA	NA	NA	NA	NA	NA

Gene	Assay_ID Applied Biosystems	Representative sequence	Sample_ID	Ct value	Ct value error	dCt value	dCt value error	ddCt value	ddCt value error	Expression level	Level Error
ESD	Hs00382661_m1	CTCCGCCACCGTAGAATCGCCTACC	10N	25.386553	0.002520711	0	0	0	0	1	0
ESD	Hs00382661_m1	CTCCGCCACCGTAGAATCGCCTACC	10T	26.430168	0.002400622	0	0	0	0	1	0
ESD	Hs00382661_m1	CTCCGCCACCGTAGAATCGCCTACC	113N	26.77119	0.075280124	0	0	0	0	1	0
ESD	Hs00382661_m1	CTCCGCCACCGTAGAATCGCCTACC	113T	28.441298	0.006368751	0	0	0	0	1	0
ESD	Hs00382661_m1	CTCCGCCACCGTAGAATCGCCTACC	136N	27.062223	0.02551715	0	0	0	0	1	0
ESD	Hs00382661_m1	CTCCGCCACCGTAGAATCGCCTACC	136T	26.152077	0.068426977	0	0	0	0	1	0
ESD	Hs00382661_m1	CTCCGCCACCGTAGAATCGCCTACC	137N	26.235407	0.006519439	0	0	0	0	1	0
ESD	Hs00382661_m1	CTCCGCCACCGTAGAATCGCCTACC	137T	28.062487	0.022318052	0	0	0	0	1	0
ESD	Hs00382661_m1	CTCCGCCACCGTAGAATCGCCTACC	147N	25.856186	0.004337633	0	0	0	0	1	0
ESD	Hs00382661_m1	CTCCGCCACCGTAGAATCGCCTACC	147T	26.184925	0.015920434	0	0	0	0	1	0
ESD	Hs00382661_m1	CTCCGCCACCGTAGAATCGCCTACC	148N	26.194876	0.036197552	0	0	0	0	1	0
ESD	Hs00382661_m1	CTCCGCCACCGTAGAATCGCCTACC	148T	25.75149	0.004265464	0	0	0	0	1	0
ESD	Hs00382661_m1	CTCCGCCACCGTAGAATCGCCTACC	154N	25.865852	0.031072991	0	0	0	0	1	0
ESD	Hs00382661_m1	CTCCGCCACCGTAGAATCGCCTACC	156N	26.082253	0.032459209	0	0	0	0	1	0
ESD	Hs00382661_m1	CTCCGCCACCGTAGAATCGCCTACC	161N	26.103931	0.015572291	0	0	0	0	1	0
ESD	Hs00382661_m1	CTCCGCCACCGTAGAATCGCCTACC	161T	26.40404	0.001068098	0	0	0	0	1	0
ESD	Hs00382661_m1	CTCCGCCACCGTAGAATCGCCTACC	165N	25.83908	0.02894026	0	0	0	0	1	0
ESD	Hs00382661_m1	CTCCGCCACCGTAGAATCGCCTACC	165T	25.717604	0.013999589	0	0	0	0	1	0
ESD	Hs00382661_m1	CTCCGCCACCGTAGAATCGCCTACC	169N	27.141241	0.001379867	0	0	0	0	1	0
ESD	Hs00382661_m1	CTCCGCCACCGTAGAATCGCCTACC	169T	26.642017	0.017292218	0	0	0	0	1	0
ESD	Hs00382661_m1	CTCCGCCACCGTAGAATCGCCTACC	16N	25.335545	0.01832452	0	0	0	0	1	0
ESD	Hs00382661_m1	CTCCGCCACCGTAGAATCGCCTACC	16T	26.54786	0.025471539	0	0	0	0	1	0
ESD	Hs00382661_m1	CTCCGCCACCGTAGAATCGCCTACC	188N	27.148785	0.005144768	0	0	0	0	1	0
ESD	Hs00382661_m1	CTCCGCCACCGTAGAATCGCCTACC	188T	26.333632	0.031245042	0	0	0	0	1	0
ESD	Hs00382661_m1	CTCCGCCACCGTAGAATCGCCTACC	202N	27.084812	0.009092689	0	0	0	0	1	0
ESD	Hs00382661_m1	CTCCGCCACCGTAGAATCGCCTACC	202T	26.555412	0.018984432	0	0	0	0	1	0
ESD	Hs00382661_m1	CTCCGCCACCGTAGAATCGCCTACC	210N	26.208302	0.004432895	0	0	0	0	1	0
ESD	Hs00382661_m1	CTCCGCCACCGTAGAATCGCCTACC	210T	26.852354	0.015349434	0	0	0	0	1	0
ESD	Hs00382661_m1	CTCCGCCACCGTAGAATCGCCTACC	215N	26.660545	0.005054702	0	0	0	0	1	0
ESD	Hs00382661_m1	CTCCGCCACCGTAGAATCGCCTACC	215T	25.58247	0.018655342	0	0	0	0	1	0
ESD	Hs00382661_m1	CTCCGCCACCGTAGAATCGCCTACC	22N	25.986692	0.002586529	0	0	0	0	1	0
ESD	Hs00382661_m1	CTCCGCCACCGTAGAATCGCCTACC	22T	25.015697	0.00146647	0	0	0	0	1	0
ESD	Hs00382661_m1	CTCCGCCACCGTAGAATCGCCTACC	233N	26.223724	0.004156922	0	0	0	0	1	0
ESD	Hs00382661_m1	CTCCGCCACCGTAGAATCGCCTACC	233T	26.039833	0.080927765	0	0	0	0	1	0
ESD	Hs00382661_m1	CTCCGCCACCGTAGAATCGCCTACC	248T	25.62476	0.020628725	0	0	0	0	1	0
ESD	Hs00382661_m1	CTCCGCCACCGTAGAATCGCCTACC	24N	25.04974	0.052059674	0	0	0	0	1	0
ESD	Hs00382661_m1	CTCCGCCACCGTAGAATCGCCTACC	24T	24.608646	0.063473889	0	0	0	0	1	0
ESD	Hs00382661_m1	CTCCGCCACCGTAGAATCGCCTACC	25N	25.020428	0.001604456	0	0	0	0	1	0
ESD	Hs00382661_m1	CTCCGCCACCGTAGAATCGCCTACC	25T	25.086212	0.011242164	0	0	0	0	1	0
ESD	Hs00382661_m1	CTCCGCCACCGTAGAATCGCCTACC	271N	26.20724	0.00922548	0	0	0	0	1	0
ESD	Hs00382661_m1	CTCCGCCACCGTAGAATCGCCTACC	271T	28.672106	0.032777329	0	0	0	0	1	0
ESD	Hs00382661_m1	CTCCGCCACCGTAGAATCGCCTACC	273N	26.537527	0.018113787	0	0	0	0	1	0
ESD	Hs00382661_m1	CTCCGCCACCGTAGAATCGCCTACC	273T	25.688406	0.013826962	0	0	0	0	1	0
ESD	Hs00382661_m1	CTCCGCCACCGTAGAATCGCCTACC	276N	26.098091	0.002886174	0	0	0	0	1	0
ESD	Hs00382661_m1	CTCCGCCACCGTAGAATCGCCTACC	276T	24.264072	0.022736054	0	0	0	0	1	0
ESD	Hs00382661_m1	CTCCGCCACCGTAGAATCGCCTACC	282N	25.845219	0.079044448	0	0	0	0	1	0
ESD	Hs00382661_m1	CTCCGCCACCGTAGAATCGCCTACC	282T	27.353994	0.048184499	0	0	0	0	1	0
ESD	Hs00382661_m1	CTCCGCCACCGTAGAATCGCCTACC	284N	26.27053	0.013993238	0	0	0	0	1	0
ESD	Hs00382661_m1	CTCCGCCACCGTAGAATCGCCTACC	301T	25.82934	0.00835599	0	0	0	0	1	0
ESD	Hs00382661_m1	CTCCGCCACCGTAGAATCGCCTACC	30N	24.934517	0.004427699	0	0	0	0	1	0
ESD	Hs00382661_m1	CTCCGCCACCGTAGAATCGCCTACC	30T	24.755316	0.006552348	0	0	0	0	1	0
ESD	Hs00382661_m1	CTCCGCCACCGTAGAATCGCCTACC	331N	26.877794	0.029086329	0	0	0	0	1	0
ESD	Hs00382661_m1	CTCCGCCACCGTAGAATCGCCTACC	331T	25.799824	0.010590913	0	0	0	0	1	0
ESD	Hs00382661_m1	CTCCGCCACCGTAGAATCGCCTACC	334N	25.951096	0.011893416	0	0	0	0	1	0
ESD	Hs00382661_m1	CTCCGCCACCGTAGAATCGCCTACC	334T	26.905657	0.009353652	0	0	0	0	1	0
ESD	Hs00382661_m1	CTCCGCCACCGTAGAATCGCCTACC	342N	28.471718	0.016828606	0	0	0	0	1	0
ESD	Hs00382661_m1	CTCCGCCACCGTAGAATCGCCTACC	342T	27.114159	0.038571039	0	0	0	0	1	0
ESD	Hs00382661_m1	CTCCGCCACCGTAGAATCGCCTACC	347T	27.409565	0.075606905	0	0	0	0	1	0
ESD	Hs00382661_m1	CTCCGCCACCGTAGAATCGCCTACC	348N	25.737713	0.044918428	0	0	0	0	1	0
ESD	Hs00382661_m1	CTCCGCCACCGTAGAATCGCCTACC	364T	26.117107	0.019534646	0	0	0	0	1	0
ESD	Hs00382661_m1	CTCCGCCACCGTAGAATCGCCTACC	368N	27.9852	0.008629654	0	0	0	0	1	0
ESD	Hs00382661_m1	CTCCGCCACCGTAGAATCGCCTACC	368T	26.832666	0.00339251	0	0	0	0	1	0

Gene	Assay_ID Applied Biosystems	Representative sequence	Sample_ID	Ct value	Ct value error	dCt value	dCt value error	ddCt value	ddCt value error	Expression level	Level Error
ESD	Hs00382661_m1	CTCCGCCACCGTAGAATCGCCTACC	378N	26.654472	0.011070114	0	0	0	0	1	0
ESD	Hs00382661_m1	CTCCGCCACCGTAGAATCGCCTACC	378T	26.728981	0.004590512	0	0	0	0	1	0
ESD	Hs00382661_m1	CTCCGCCACCGTAGAATCGCCTACC	37N	25.754335	0.023613626	0	0	0	0	1	0
ESD	Hs00382661_m1	CTCCGCCACCGTAGAATCGCCTACC	37T	25.19835	0.037534118	0	0	0	0	1	0
ESD	Hs00382661_m1	CTCCGCCACCGTAGAATCGCCTACC	390N	25.953579	0.073250161	0	0	0	0	1	0
ESD	Hs00382661_m1	CTCCGCCACCGTAGAATCGCCTACC	390T	26.153212	0.009766457	0	0	0	0	1	0
ESD	Hs00382661_m1	CTCCGCCACCGTAGAATCGCCTACC	397T	26.455261	0.046145298	0	0	0	0	1	0
ESD	Hs00382661_m1	CTCCGCCACCGTAGAATCGCCTACC	400T	26.111197	0.023524137	0	0	0	0	1	0
ESD	Hs00382661_m1	CTCCGCCACCGTAGAATCGCCTACC	402T	26.164217	0.032137625	0	0	0	0	1	0
ESD	Hs00382661_m1	CTCCGCCACCGTAGAATCGCCTACC	40N	24.758432	0.003384427	0	0	0	0	1	0
ESD	Hs00382661_m1	CTCCGCCACCGTAGAATCGCCTACC	40T	26.572056	0.012612794	0	0	0	0	1	0
ESD	Hs00382661_m1	CTCCGCCACCGTAGAATCGCCTACC	412T	25.875551	0.005687478	0	0	0	0	1	0
ESD	Hs00382661_m1	CTCCGCCACCGTAGAATCGCCTACC	421N	26.69746	0.018736748	0	0	0	0	1	0
ESD	Hs00382661_m1	CTCCGCCACCGTAGAATCGCCTACC	421T	26.478216	0.02354954	0	0	0	0	1	0
ESD	Hs00382661_m1	CTCCGCCACCGTAGAATCGCCTACC	426N	25.943502	0.01588637	0	0	0	0	1	0
ESD	Hs00382661_m1	CTCCGCCACCGTAGAATCGCCTACC	426T	25.994095	0.00191969	0	0	0	0	1	0
ESD	Hs00382661_m1	CTCCGCCACCGTAGAATCGCCTACC	42N	26.132732	0.016495475	0	0	0	0	1	0
ESD	Hs00382661_m1	CTCCGCCACCGTAGAATCGCCTACC	42T	26.041634	0.056638639	0	0	0	0	1	0
ESD	Hs00382661_m1	CTCCGCCACCGTAGAATCGCCTACC	434N	26.937828	0.078295047	0	0	0	0	1	0
ESD	Hs00382661_m1	CTCCGCCACCGTAGAATCGCCTACC	434T	27.229864	0.063231401	0	0	0	0	1	0
ESD	Hs00382661_m1	CTCCGCCACCGTAGAATCGCCTACC	445N	26.713295	0.009807449	0	0	0	0	1	0
ESD	Hs00382661_m1	CTCCGCCACCGTAGAATCGCCTACC	445T	26.826536	0.0328622	0	0	0	0	1	0
ESD	Hs00382661_m1	CTCCGCCACCGTAGAATCGCCTACC	489T	26.208475	0.019180731	0	0	0	0	1	0
ESD	Hs00382661_m1	CTCCGCCACCGTAGAATCGCCTACC	492N	26.174326	0.042404068	0	0	0	0	1	0
ESD	Hs00382661_m1	CTCCGCCACCGTAGAATCGCCTACC	492T	27.244207	0.030882466	0	0	0	0	1	0
ESD	Hs00382661_m1	CTCCGCCACCGTAGAATCGCCTACC	49N	25.407951	0.033087944	0	0	0	0	1	0
ESD	Hs00382661_m1	CTCCGCCACCGTAGAATCGCCTACC	49T	25.417877	0.00060795	0	0	0	0	1	0
ESD	Hs00382661_m1	CTCCGCCACCGTAGAATCGCCTACC	51N	26.095554	0.013385289	0	0	0	0	1	0
ESD	Hs00382661_m1	CTCCGCCACCGTAGAATCGCCTACC	51T	26.370825	0.006046589	0	0	0	0	1	0
ESD	Hs00382661_m1	CTCCGCCACCGTAGAATCGCCTACC	53N	25.868956	0.061804192	0	0	0	0	1	0
ESD	Hs00382661_m1	CTCCGCCACCGTAGAATCGCCTACC	53T	25.93102	0.000844086	0	0	0	0	1	0
ESD	Hs00382661_m1	CTCCGCCACCGTAGAATCGCCTACC	55N	27.515036	0.006789062	0	0	0	0	1	0
ESD	Hs00382661_m1	CTCCGCCACCGTAGAATCGCCTACC	55T	25.560581	0.005529284	0	0	0	0	1	0
ESD	Hs00382661_m1	CTCCGCCACCGTAGAATCGCCTACC	65N	27.179117	0.048246275	0	0	0	0	1	0
ESD	Hs00382661_m1	CTCCGCCACCGTAGAATCGCCTACC	70T	25.477037	0.023630947	0	0	0	0	1	0
ESD	Hs00382661_m1	CTCCGCCACCGTAGAATCGCCTACC	75N	27.480774	0.025449023	0	0	0	0	1	0
ESD	Hs00382661_m1	CTCCGCCACCGTAGAATCGCCTACC	77N	26.137497	0.005376286	0	0	0	0	1	0
ESD	Hs00382661_m1	CTCCGCCACCGTAGAATCGCCTACC	77T	29.527004	0.036881713	0	0	0	0	1	0
ESD	Hs00382661_m1	CTCCGCCACCGTAGAATCGCCTACC	84N	26.437515	0.034243222	0	0	0	0	1	0
ESD	Hs00382661_m1	CTCCGCCACCGTAGAATCGCCTACC	84T	25.808266	0.024445588	0	0	0	0	1	0
ESD	Hs00382661_m1	CTCCGCCACCGTAGAATCGCCTACC	88N	26.174444	0.059307729	0	0	0	0	1	0
ESD	Hs00382661_m1	CTCCGCCACCGTAGAATCGCCTACC	88T	25.832037	0.093776695	0	0	0	0	1	0
ESD	Hs00382661_m1	CTCCGCCACCGTAGAATCGCCTACC	89T	27.114267	0.009752601	0	0	0	0	1	0
ESD	Hs00382661_m1	CTCCGCCACCGTAGAATCGCCTACC	95N	26.827026	0.006725553	0	0	0	0	1	0
ESD	Hs00382661_m1	CTCCGCCACCGTAGAATCGCCTACC	95T	26.557407	0.009701217	0	0	0	0	1	0
ESD	Hs00382661_m1	CTCCGCCACCGTAGAATCGCCTACC	97N	25.938107	0.011180965	0	0	0	0	1	0
ESD	Hs00382661_m1	CTCCGCCACCGTAGAATCGCCTACC	NTC	NA	NA	NA	0	NA	0	NA	NA
