# Supplementary material for: Loss of aquaporin-4 expression and putative function in non-small cell lung cancer
Source: BMC Cancer. 2011 May 6;11:161. doi: 10.1186/1471-2407-11-161 (PMC3098822; doi:10.1186/1471-2407-11-161)
Supplement: Additional file 5 — Table S5. Clinicopathologic data of the tissue microarray specimens. Patient data including survival status, follow-up, AQP4 immunoreactivity score (IRS) and tumor staging. [file 1471-2407-11-161-S5.PDF]

Supplemental Table 5: Patient data including survival status, follow-up, AQP4 immunoreactivity score (IRS) and tumor staging.

Sex	Status	Age/years	Follow-up/days	Follow-up/months	IRS AQP4	Histology	T	N	M	R
m	alive	46.8	1617	53.9	12	AC	1	1	0	0
m	alive	75.9	1582	52.7	4	AC	2	0	0	0
m	alive	55.4	1623	54.1	4	AC	2	0	0	0
m	alive	60.4	1113	37.1	0	AC	2	2	0	0
m	alive	75.4	1516	50.5	0	AC	2	0	0	0
f	alive	73.4	1876	62.5	8	AC	2	0	0	0
m	alive	65.1	1044	34.8	3	AC	2	0	0	0
f	alive	67.1	1005	33.5	9	AC	1	0	0	0
m	alive	61.2	1938	64.6	3	AC	2	0	0	0
f	alive	67.4	1677	55.9	12	AC	1	0	0	0
m	dead	62.4	1252	41.7	4	AC	1	0	0	0
f	dead	75.2	377	12.6	0	AC	2	0	0	0
m	alive	52.8	1843	61.4	3	AC	2	0	0	0
f	alive	63.0	1849	61.6	8	AC	2	0	0	0
f	dead	63.0	873	29.1	8	AC	2	0	0	0
m	alive	66.9	1503	50.1	12	AC	2	0	0	0
f	alive	72.7	920	30.7	12	AC	1	0	0	0
f	alive	53.1	1023	34.1	2	AC	1	0	0	0
m	alive	73.5	1112	37.1	1	AC	2	0	0	0
m	alive	42.3	1183	39.4	12	AC	2	0	0	0
m	dead	61.7	984	32.8	0	AC	2	0	0	0
f	dead	76.2	1846	61.5	4	AC	1	0	0	0
f	alive	51.2	1363	45.4	4	AC	1	0	0	0
f	alive	62.1	1457	48.6	8	AC	1	0	0	0
m	alive	55.0	2103	70.1	3	AC	2	1	0	0
m	alive	60.3	1184	39.5	8	AC	2	0	0	0
f	alive	53.0	1794	59.8	6	AC	1	1	0	0
m	alive	61.3	1321	44.0	2	AC	2	0	0	0
f	alive	67.5	1191	39.7	0	AC	2	0	0	0
f	dead	79.1	1048	34.9	0	AC	2	1	0	0
f	alive	55.1	1326	44.2	2	AC	2	0	0	0
f	alive	55.8	1345	44.8	1	AC	2	0	0	0
m	alive	66.4	1216	40.5	0	AC	2	0	0	0
m	alive	76.1	1274	42.5	0	AC	2	0	0	0
m	alive	57.9	808	26.9	0	AC	1	0	0	0
m	alive	61.8	1336	44.5	2	AC	2	1	0	0
f	alive	64.0	604	20.1	4	AC	2	0	0	0
m	dead	62.2	440	14.7	3	AC	2	0	0	0
m	dead	60.2	168	5.6	0	AC	2	1	0	0
m	dead	64.2	325	10.8	2	AC	2	0	0	0
f	dead	53.1	460	15.3	0	AC	2	0	0	0
f	dead	62.9	1009	33.6	2	AC	2	0	0	0
f	dead	59.4	658	21.9	8	AC	2	0	0	0
m	dead	64.0	815	27.2	2	AC	2	0	0	0
m	dead	59.0	442	14.7	4	AC	2	0	0	0
m	dead	72.3	472	15.7	3	AC	2	0	0	0
